# Imagery ability assessments: a cross-disciplinary systematic review and quality evaluation of psychometric properties

**DOI:** 10.1186/s12916-022-02295-3

**Published:** 2022-05-02

**Authors:** Zorica Suica, Frank Behrendt, Szabina Gäumann, Ulrich Gerth, Arno Schmidt-Trucksäss, Thierry Ettlin, Corina Schuster-Amft

**Affiliations:** 1 Research Department, Reha Rheinfelden, Salinenstrasse 98, CH-4310 Rheinfelden, Switzerland; 2 Institute for Rehabilitation and Performance Technology, Bern University of Applied Sciences, 3401 Burgdorf, Switzerland; 3 Department for Sport, Exercise and Health, University of Basel, 4052 Basel, Switzerland

**Keywords:** Motor imagery, Mental imagery, Assessment, Psychometric properties, Validity, Reliability, Responsiveness

## Abstract

**Background:**

Over the last two centuries, researchers developed several assessments to evaluate the multidimensional construct of imagery. However, no comprehensive systematic review (SR) exists for imagery ability evaluation methods and an in-depth quality evaluation of their psychometric properties.

**Methods:**

We performed a comprehensive systematic search in six databases in the disciplines of sport, psychology, medicine, education: SPORTDiscus, PsycINFO, Cochrane Library, Scopus, Web of Science, and ERIC. Two reviewers independently identified and screened articles for selection. COSMIN checklist was used to evaluate the methodological quality of the studies. All included assessments were evaluated for quality using criteria for good measurement properties. The evidence synthesis was summarised by using the GRADE approach.

**Results:**

In total, 121 articles reporting 155 studies and describing 65 assessments were included. We categorised assessments based on their construct on: (1) motor imagery (*n* = 15), (2) mental imagery (*n* = 48) and (3) mental chronometry (*n* = 2). Methodological quality of studies was mainly doubtful or inadequate. The psychometric properties of most assessments were insufficient or indeterminate. The best rated assessments with sufficient psychometric properties were MIQ, MIQ-R, MIQ-3, and VMIQ-2 for evaluation of motor imagery ability. Regarding mental imagery evaluation, only SIAQ and VVIQ showed sufficient psychometric properties.

**Conclusion:**

Various assessments exist to evaluate an individual’s imagery ability within different dimensions or modalities of imagery in different disciplines. However, the psychometric properties of most assessments are insufficient or indeterminate. Several assessments should be revised and further validated. Moreover, most studies were only evaluated with students. Further cross-disciplinary validation studies are needed including older populations with a larger age range. Our findings allow clinicians, coaches, teachers, and researchers to select a suitable imagery ability assessment for their setting and goals based on information about the focus and quality of the assessments.

**Systematic reviews register:**

PROSPERO CRD42017077004.

**Supplementary Information:**

The online version contains supplementary material available at 10.1186/s12916-022-02295-3.

## Background

Imagery, defined as the representation and the accompanying experience of any sensory information without a direct external stimulus [1], or ‘seeing with the mind’s eye’, ‘hearing with the mind’s ear’ [2], is a fundamental cognitive process. For example, imagery can be helpful in decision-making or problem solving processes [3], in emotion regulation [4], for motor learning and performance [5]. In sports, a strong imagery ability in athletes is associated with more successful and better performance [6, 7]. At the same time, several psychological disorders, such as posttraumatic stress disorder, depression, or social phobia, are associated with dysfunctions in imagery ability [8, 9]. In this context, the application of different imagery techniques showed positive effects in the treatment of psychological disorders [8], for pain treatment (guided imagery) [10], and to enhance motor rehabilitation in patients with neurological and orthopaedic disorders [11–18] as well as to enhance psychomotor skills or various aspects of performance in athletes (motor imagery) [19]. The benefits of imagery depend on the individual capability to imagine [20] and it is deemed essential to assess imagery abilities prior to interventions [21].

Imagery is a multidimensional construct [22] with wide individual differences regarding preference of imagery (verbal and visual style), imagery control or imagery vividness [23, 24]. The pioneering work from Betts in 1909 [25] already described and measured vividness of imagery in seven sensory modalities: visual, auditory, cutaneous, kinaesthetic, gustatory, olfactory and organic (e.g. feeling or emotion). Further research focused on additional dimensions of imagery clarity [26, 27], controllability [28], the ease and accuracy with which an image can be manipulated mentally [29, 30] and imagery perspective [7, 31]. Moreover, studies in cognitive and neuroscience [32, 33] assert that imagery is not unitary, and distinguished two types: spatial imagery and object imagery [34]. Object imagery is defined as representations of the visual appearances of objects or scenes in terms of their precise form, size, shape and colour, whereas spatial imagery refers to rather abstract representations of the spatial relations among objects, parts of objects, locations of objects in space, movements of objects, object parts and other complex spatial transformations [34, 35].

**Fig. 1:**
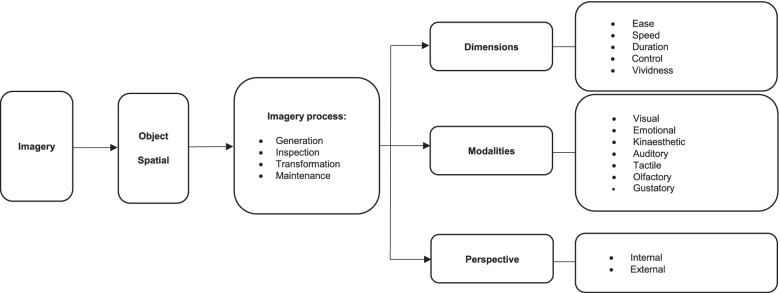
Proposed model for multidimensional and multimodal structure of imagery ability

Watt [36] and Cumming et al. [37] proposed a hierarchical model to explain the imagery process and components of imagery ability in sports. However, types of imagery are missing in their model. Now, we have revised this model and expanded it with the object and spatial type of imagery (Fig. 1).

The measurement of this multidimensional and multimodal construct has proven to be complex [38] and each type of assessments evaluates a different aspect of imagery ability [39]. Over the past century, various assessments have been developed to evaluate an individual’s imagery ability considering different dimensions, sensory modalities, different perspectives, image manipulation, or the temporal coupling between real and imagined movements [7, 26, 27, 34, 40–44]. Most of those assessments are self-reported questionnaires (subjective assessments) and focus on object imagery. In contrast, the objective assessments focus more on spatial imagery [39]. However, the literature lacks a systematic literature review of imagery evaluation methods and the evaluation of their measurement properties. Two previous narrative [45, 46] and one systematic [47] reviews mainly focused on assessments of a single imagery technique: motor imagery. In addition, these reviews only included assessments of motor imagery in the field of neurology or sports. Further, only two reviews reported the assessments’ psychometric properties [45, 47]). White et al. [48] evaluated self-report assessments of imagery, but all other assessments, developed or modified after that are missing in his review.

The aim of the present extensive and comprehensive systematic literature review was therefore to evaluate all available imagery ability assessments across four disciplines, regardless of the imagery technique used to answer the question: What imagery ability assessments exist in the fields of sports, psychology, medicine, and education, and what are their psychometric properties? For the interested clinician, coach, teacher, and researcher, our review provides (1) a systematic classification of the imagery ability assessments based on its construct, (2) a summary of the current level of evidence for the psychometric properties of the selected imagery ability assessments, and (3) all specific characteristics of the imagery ability assessment: version, subscales, scoring, equipment needed, etc.

In order to provide a comprehensive overview, we included all assessments that cover any aspect of imagery process and ability to vividly generate, transform, inspect, and maintain a mental image. Moreover, we included also assessments, which evaluated the frequency of use of imagery, the preference to think in words or images, and the temporal coupling of mental and physical practice.

This systematic review provides interested readers with a quick overview to select an appropriate imagery ability assessment for their current setting and goals based on information provided regarding the focus and quality of the imagery ability assessments.

## Methods

### Study design and registration

The protocol for this review was registered with the International Prospective Register of Systematic Reviews (PROSPERO; https://www.crd.york.ac.uk/prospero/, registration number CRD42017077004) and published [49]. The present systematic review was written and reported using the Preferred Reporting Items for Systematic review and Meta-Analysis (PRISMA) guidelines, the PRISMA checklist, and the PRISMA abstract checklist [50, 51]. Additionally, we followed the recommendations for systematic reviews on measurement properties [52, 53].

### Search strategy

We searched in four fields of interest: sports, psychology, medicine, and education. One author (ZS) and a librarian from the medical library of the University of Zurich independently performed the electronic search between September and October, 2017, in SPORTDiscus (1892 to current date of search), PsycINFO (1887 to current date of search), Cochrane Library (current issue), Scopus (1996 to current date of search), Web of Science (1900 to current date of search) and ERIC (1966 to current date of search). The search strategy included (1) construct: motor imagery, mental imagery, mental rehearsal, movement imagery, mental practice, mental training; (2) instrument: measure, questionnaire, scale, assessment; and (3) the filter for measurement properties by Terwee et al. [54] adapted for each database (Additional file 1: AF_1_Example search strategy_ Web of Science). An update of the search in all databases was performed in January 2021.

### Selection criteria

There was no limitation on a specific population (e.g. healthy individuals, adults, children, and patients). Additionally, there was no restriction on age, gender, or health status. We included all original articles published in English and German, which either developed mental or motor imagery assessments or validated their psychometric properties.

Articles were excluded if the authors only used neurophysiological methods to evaluate imagery ability (e.g. functional magnetic resonance imaging, electroencephalography, or brain-computer interface technology).

### Selection process

**Fig. 2:**
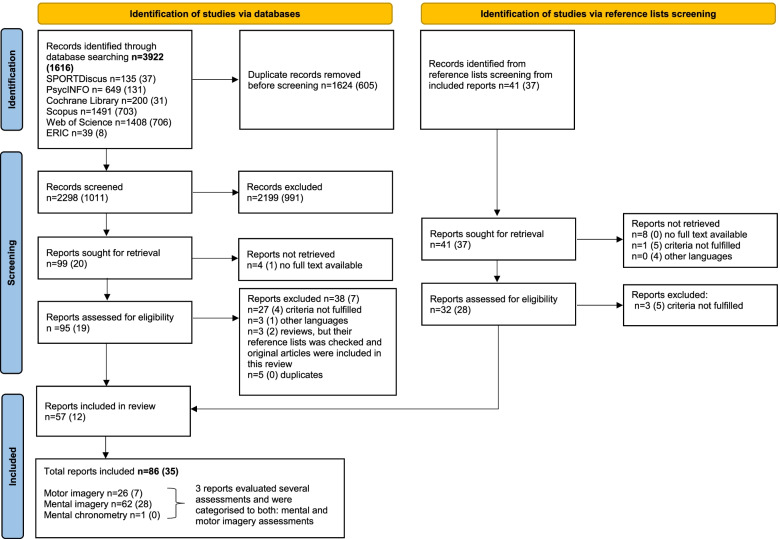
The literature search and study selection process. *n* = number of references. Numbers in brackets indicate references retrieved from the search in January 2021

Figure 2 provides an overview of all databases and identified references. All citations were imported into the reference management software package EndNote (version X7; Thomson Reuters, New York, USA). De-duplication was performed by the librarian, who performed the original search. To examine the agreement and disagreement regarding studies’ eligibility between the two reviewers (ZS and CSA) in the preselection phase, 10% of all articles were randomly selected and screened by both reviewers. After preselection, titles, abstracts, and full texts from all identified articles were independently screened. Full texts were ordered if no decision could be made based on the available information. If no full text was available, the corresponding authors of the articles were contacted to obtain the missing papers. Disagreement of selected full texts was discussed by both reviewers, and if both reviewers were not able to agree on a decision a third reviewer would have been consulted to decide on in- or exclusion (which was not the case in this review). The Kappa statistic was calculated and interpreted in accordance with Landis and Koch’s benchmarks for assessing the inter-reviewer agreement: poor (0), slight (0.0 to 0.20), fair (0.21 to 0.40), moderate (0.41 to 0.60), substantial (0.61 to 0.80), and almost perfect (0.81 to 1.0) [55]. The percentage agreement between the raters was also calculated [56].

### Data extraction

Four researchers (ZS, SG, LM, and VZ) performed the data extraction into Microsoft Excel (Version 14.0, 2010, Microsoft Corp., Redmond, California, USA). ZS checked all data for accuracy. The following data were extracted: (1) characteristics of included articles: first author, year of publication, country of origin, study design, and number and main characteristics of participants (e.g. age, gender, and target population); (2) general characteristics of the assessment instrument: name, language, version, construct of evaluation, number of items, subscales, scoring, assessment format, time and equipment needed, examiner qualifications, and costs; and (3) data on the psychometric properties of the assessments: validity, reliability, and responsiveness.

### Studies’ methodological quality: risk of bias rating

Two researches (ZS and CSA) carried out the COnsensus-based Standards for the selection of health Measurement INstruments (COSMIN) evaluation independently. One study was evaluated by ZS and FB, because CSA was the first author. The COSMIN Risk of Bias checklist was applied to assess the methodological quality of studies on measurement properties [57]. The COSMIN Risk of Bias checklist contains ten boxes with standards for Patient-Reported Outcome Measures (PROM) development, and for nine measurement properties: content validity, structural validity, internal consistency, cross-cultural validity, reliability, measurement error, criterion validity, hypotheses testing for construct validity and responsiveness. A 4-point rating system as ‘very good’, ‘adequate’, ‘doubtful’ and ‘inadequate’ was used for study evaluation (Additional file 2: AF_2_COSMIN_RoB_checklist). The overall rating of quality of each study was determined according to the lowest rating of any standard in the box (‘the worst score counts’ principle) [58].

### Quality assessment of included instruments and GRADE approach

Based on the quality criteria for measurement properties proposed by Terwee et al. [59] and updated by Prinsen et al. [60] (Table 1), the measurement properties reported in the included studies were rated as positive, negative, or indeterminate. However, no criteria are defined to assess the quality of structural validity when authors only performed an explorative factor analysis (EFA). In this case, we followed the recommendation of de Vet et al. [52], Izquierdo et al. [61] and Watkins [62] and considered (1) number of extracted factors; (2) factor loading, that should be > 0.40; (3) items with loading ≥ 0.30 on at least two factors should be candidates for deletion; (4) correlation between factors and (5) the variance explained by the factors which should be > 50%. Guidelines for judging psychometric properties of imagery instruments by McKelvie [63] were also taken into account if there were any uncertainties.

**Table 1:** Updated criteria for good measurement properties by Prinsen et al. [60]

Measurement property	Rating	Criteria
Structural validity	+	**CTT** CFA: CFI or TLI or comparable measure > 0.95 OR RMSEA < 0.06 OR SRMR < 0.08^a^ **IRT/Rasch** No violation of unidimensionality^b^: CFI or TLI or comparable measure > 0.95 OR RMSEA < 0.06 OR SRMR < 0.08 AND No violation of local independence: residual correlations among the items after controlling for the dominant factor < 0.20 OR Q3’s < 0.37 AND No violation of monotonicity: adequate looking graphs OR item scalability > 0.30 AND Adequate model fit IRT: *χ*^2^ > 0.001 Rasch: infit and outfit mean squares ≥ 0.5 and ≤ 1.5 OR Z-standardised values > -2 and < 2
	?	CTT: not all information for ‘+’ reported IRT/Rasch: model fit not reported
	−	Criteria for ‘+’ not met
Internal consistency	+	At least low evidence^c^ for sufficient structural validity^d^ AND Cronbach’s alpha(s) ≥ 0.70 for each unidimensional scale or subscale^e^
	?	Criteria for “At least low evidence^c^ for sufficient structural validity^d^” not met
	−	At least low evidence^c^ for sufficient structural validity^d^ AND Cronbach’s alpha(s) < 0.70 for each unidimensional scale or subscale^e^
Reliability	+	ICC or weighted Kappa ≥ 0.70
	?	ICC or weighted Kappa not reported
	−	ICC or weighted Kappa < 0.70
Measurement error	+	SDC or LoA < MIC^d^
	?	MIC not defined
	−	SDC or LoA > MIC^d^
Hypotheses testing for construct validity	+	The result is in accordance with the hypothesis^f^
	?	No hypothesis defined (by the review team)
	−	The result is not in accordance with the hypothesis^f^
Cross-cultural validity\measurement invariance	+	No important differences found between group factors (such as age, gender, language) in multiple group factor analysis OR no important DIF for group factors (McFadden’s *R*^2^ < 0.02)
	?	No multiple group factor analysis OR DIF analysis performed
	−	Important differences between group factors OR DIF was found
Criterion validity	+	Correlation with gold standard ≥ 0.70 OR AUC ≥ 0.70
	?	Not all information for ‘+’ reported
	−	Correlation with gold standard < 0.70 OR AUC < 0.70
Responsiveness	+	The result is in accordance with the hypothesis^f^ OR AUC ≥ 0.70
	?	No hypothesis defined (by the review team)
	−	The result is not in accordance with the hypothesis^f^ OR AUC < 0.70

The criteria are based on Terwee et al.  [59]

*AUC* Area under the curve, *CFA* Confirmatory factor analysis, *CFI* Comparative fit index, *CTT* Classical test theory, *DIF* Differential item functioning, *ICC* Intraclass correlation coefficient, *IRT* Item response theory, *LoA* Limits of agreement, *MIC* Minimal important change, *RMSEA* Root mean square error of approximation, *SDC* Smallest detectable change, *SRMR* Standardised root mean residuals, *TLI* Tucker–Lewis index

‘+’ sufficient, ‘-‘ insufficient, ʻ?ʼ indeterminate

^a^To rate the quality of the summary score, the factor structures should be equal across studies

^b^Unidimensionality refers to a factor analysis per subscale, while structural validity refers to a factor analysis of a (multidimensional) Patient-Reported Outcome Measure

^c^As defined by grading the evidence according to the GRADE approach

^d^This evidence may come from different studies

^e^The criteria ‘Cronbach alpha < 0.95’ was deleted, as this is relevant in the development phase of a PROM and not when evaluating an existing PROM

^f^The results of all studies should be taken together and it should then be decided if 75% of the results are in accordance with the hypotheses

Regarding the testing for construct validity, some hypotheses about expected differences between instruments were formulated by the reviewer team:

1. Strong correlation (at least 0.50) was expected if a related construct was measured with the comparator instrument.
2. Correlation between different modalities or dimensions of imagery, e.g. between vividness and auditory imagery, should be very low (< 0.30).
3. Correlation between subjective and objective assessments of imagery ability should be very low (< 0.30).
4. Regarding known-group validity based on previous evidence, no any sex differences regarding imagery ability were expected.

Just recently, a modified Grading of Recommendations Assessment, Development, and Evaluation (GRADE) approach for grading the quality of the evidence in systematic reviews of PROMs was introduced [53]. Four of the five GRADE factors have been adopted for evaluating measurement properties in systematic reviews of PROMs: risk of bias (e.g. the methodological quality of the studies), inconsistency (e.g. unexplained inconsistency of results across studies), imprecision (e.g. total sample size of the available studies) and indirectness (e.g. evidence from different populations than the population of interest in the review). The GRADE approach was applied if studies evaluated the same instrument regarding language and version and the same population. Studies reporting psychometric properties of assessments tested with athletes and students were not pooled. Using the modified GRADE approach, the quality of the evidence is graded as high, moderate, low or very low (Table 2) [53, 64].

**Table 2:** Modified GRADE

Quality of evidence	Lower if
High	**Risk of bias**
Moderate	− 1 Serious
Low	− 2 Very serious
Very low	− 3 Extremely serious
	**Inconsistency**
	− 1 Serious
	− 2 Very serious
	**Imprecision**
	− 1 total *n* = 50–100
	− 2 total *n* < 50
	**Indirectness**
	− 1 Serious
	− 2 Very serious

The starting point is the assumption that the evidence is of high quality. The quality of evidence is subsequently downgraded with one or two levels for each factor (e.g. risk of bias, inconsistency, imprecision, indirectness) to moderate, low or very low when there is risk of bias (low study quality), (unexplained) inconsistency in results, or indirect results. *N* sample size

## Results

In total, 3922 references were retrieved in October, 2017. The search update in January 2021 resulted in 1616 additional references. We identified 78 additional references through reference list screening. The kappa statistic after screening of titles and abstracts was 0.83 (almost perfect), and the percentage agreement between the raters was 98%. After selecting the full texts, the kappa was 0.76 (substantial) and 85% percentage agreement was established. All distinguish between reviews have been discussed and the reviews agree on a decision.

Finally, 121 articles reporting 155 studies and describing 65 assessments from four disciplines were included in the present review. We categorised assessments based on their construct:

1. Motor imagery = movement imagery without engaging in its physical execution
2. Mental imagery in four sub-categories:
  - General mental imagery in any sensorial modality,
  - Spatial imagery or mental rotation = ability to rotate or manipulate mental images),
  - Distinguish between use of different cognitive style (e.g. verbal versus visual), and
  - Use of mental imagery (frequency of use in daily life).
3. Mental chronometry as temporal coupling between real and imagined movements.

Most studies were carried out in the fields of psychology and sport. We identified many assessments, which have been evaluated only with psychology students. Therefore, it was unclear whether those assessments should accordingly only be applied in the field of psychology. We defined such assessments as ‘not discipline specific’. Moreover, most studies evaluated different psychometric properties and according to COSMIN, each evaluation of a measurement property was separately assessed on its methodological quality. The overall rating of the quality of each study should be determined by taking the lowest rating of any standard in the box (e.g. ‘the worst score counts’ principle) [58]. Furthermore, it was difficult to define a reasonable ‘gold standard’ for assessing criterion validity. If the authors correlated the score of a new instrument with an already established, widely used and well-known instrument, we considered the comparison as test for construct validity. Only if a shortened version was compared with the original version, we considered the comparison as test for criterion validity (proposed by COSMIN [64]).

### Motor imagery assessments

In total, 33 out of the 121 articles focused on 15 motor imagery assessments: Florida Praxis Imagery Questionnaire (FPIQ), Imaprax, Kinesthetic and Visual Imagery Questionnaire (KVIQ-20) and short version KVIQ-10, Movement Imagery Questionnaire (MIQ), Revised Movement Imagery Questionnaire (MIQ-R), Movement Imagery Questionnaire-Revised second version (MIQ-RS), Movement Imagery Questionnaire-3 (MIQ-3), Movement Imagery Questionnaire for Children (MIQ-C), Test of Ability in Movement Imagery (TAMI), Test of Ability in Movement Imagery with Hands (TAMI-H), Vividness of Movement Imagery Questionnaire (VMIQ), Vividness of Haptic Movement Imagery Questionnaire (VHMIQ), Revised Vividness of Movement Imagery Questionnaire-2 (VMIQ-2) and the Wheelchair Imagery Ability Questionnaire (WIAQ). The characteristics of the included studies, their ‘risk of bias assessment/rating’, and their psychometric properties are presented in Tables 3 and 4. The general characteristics of included instruments are presented in the Additional file 3: Table 1S.

**Table 3:** Motor imagery assessments: The characteristics of the included studies - Reliability

Tool	Disciplines	Study	Country	Language	Study population				Reliability		COSMIN	Quality criteria	Comments
					Participants	***N***	Age mean (years)	Sex	Design	Results			
Florida Praxis Imagery Questionnaire (FPIQ)	Med	Ochipa et al. 1997 [65]	USA	E	Apraxia patient	1	61.0	1♀	NR	NR	NA	NA	Case report, first mention of FPIQ, no psychometric properties evaluated, no information about FPIQ development.
Imaprax	NR	Fournier 2000 [66]	FR	F	NR	10	NR	NR	Development	NR	Inadequate	NA	Development study, no psychometric properties evaluated.
	Med	Schuster et al. 2012 [67]	CH	G	Subacute group^a^	17	65.0	8♀, 9♂	Test-retest	Visual ICC=0.84 (95% CI 0.62–0.94)^a^ ICC=0.34 (95% CI 0.005–0.60)^b^ ICC=0.77 (95% CI 0.19–0.95)^c^ ICC=0.37 (95% CI - 0.40–0.85)^d^ ICC=0.74 (95% CI 0.14–0.95)^e^	Doubtful	?	Small sample size in four of five groups. The smallest ICC was by group with largest sample size.
					Chronic group^b^	34	62.5	9♀, 25♂					
					Left parietal lobe^c^	7	61.6	3♀, 4♂					
					MS^d^	7	48.0	5♀, 2♂	Internal consistency	*α*=0.70	Very good	?	*Insufficient information for quality criteria rating.
					PD^e^	8	73.4	3♀, 5♂					
Kinaesthetic and Visual Imagery Questionnaire (KVIQ)	Med	Malouin et al. 2007 [43]	CA	E	Stroke^a^	19	58.6	5♀, 14♂	Test-retest	**KVIQ-20 / KVIQ-10** kinaesthetic ICC=0.89 (CI_LL_=0.75)^a^**/**0.88 (CI_LL_=0.71)^a^ ICC=0.79 (CI_LL_=0.65)^b^**/**0.81 (CI_LL_=0.68)^b^ ICC=0.73 (CI_LL_=0.43)^c^**/** 0.74 (CI_LL_=0.45)^c^ visual ICC=0.81 (CI_LL_=0.57)^a^ /0.82 (CI_LL_=0.59)^a^ ICC=0.73 (CI_LL_=0.57)^b^ /0.72 (CI_LL_=0.54)^b^ ICC=0.80 (CI_LL_=0.55)^c^ /0.78 (CI_LL_=0.52)^c^	Doubtful	+	CI_LL_=confidence interval lower limit. Sample size calculation not mentioned. Small sample size in stroke and age-matched groups.
					Healthy^b^	46	43.4	33♀, 13♂					
					Age-matched healthy^c^	19	59.7	11♀, 8♂					
					Stroke	33	60.1	7♀, 26♂	Internal consistency	**KVIQ-20 / KVIQ-10** Kinaesthetic *α*=0.92/ *α*=0.87 Visual *α*=0.94/ *α*=0.89	Very good	+	Very good sample size for this analysis.
					Healthy	70	42.9	49♀, 21♂					
					LL amputation	13	35.0	13♂					
					Acquired blindness	10	40.8	4♀, 6♂					
					LL immobilisation	5	50.1	5♂					
	Med	Randhawa et al. 2010 [68]	CA	E	PD	11	61.7	7♀, 4♂	Test-retest	Kinaesthetic ICC=0.95 (CI_LL_=0.83) Visual ICC=0.82 (0.49)	Inadequate	+	Low sample size considered as very important flaws- axial movements were not reliable, but only 1 patient had deficits in axial movement.
Kinaesthetic and Visual Imagery Questionnaire (KVIQ)	Med	Schuster et al. 2012 [67]	CH	G	Subacute stroke^a^	17	65.0	8♀, 9♂	Test-retest	**KVIQ-G-20/ KVIQ-G-10** Kinaesthetic (95% CI) ICC=0.80 (0.54–0.92)^a^/0.79 (0.51–0.92)^a^ ICC=0.75 (0.56–0.87)^b^/0.80 (0.64–0.89)^b^ ICC=0.91 (0.61–0.98)^c^/0.88 (- 0.52–0.98)^c^ ICC=0.95 (0.75–0.99)^d^/0.92 (0.66–0.99)^d^ ICC=0.82 (0.39–0.96)^e^/0.84 (0.44–0.97)^e^ Visual (95% CI) ICC=0.83 (0.60–0.94)^a^/0.86 (0.66–0.95)^a^ ICC=0.84 (0.71–0.92)^b^/0.82 (0.67–0.90)^b^ ICC=0.77 (0.20–0.96)^c^/0.62 (- 0.10–0.90)^c^ ICC=0.43 (- 0.35–0.87)^d^/0.51 (- 0.67–0.94)^d^ ICC=0.68 (0.08–0.93)^e^/0.69 (0.10–0.89)^e^	Doubtful	+	Sample size calculation not mentioned. Small sample size in MS and PD groups. MS group showed lowest ICCs in the visual subscale.
					Chronic stroke^b^	34	62.5	9♀, 25♂					
					Left parietal lobe^c^	7	61.6	3♀, 4♂					
					MS^d^	7	48.0	5♀, 2♂					
					PD^e^	8	73.4	3♀, 5♂					
									Internal consistency	**KVIQ-G-20/ KVIQ-G-10** Kinaesthetic *α*=0.96/ *α*=0.92 Visual *α*=0.94/ *α*=0.88	Very good	?	Adequate sample size for this analysis. Structural validity indeterminate.
	Med	Tabrizi et al, 2013 [69]	IR	NR	MS	15	31.7	12♀, 3♂	Test-retest	Kinaesthetic ICC=0.93 (*p*<0.001) Visual ICC=0.85 (*p*<0.001)	Inadequate	+	Language version of KVIQ not mentioned. Sample size insufficient for this analysis.
									Internal consistency	*α*=0.84	Inadequate	?	Cronbach’s alpha was calculated for total score and not for each subscales.
	Med	Demanboro et al. 2018 [70]	BR	P	Stroke^a^	33^a^	54.8^a^	NR	Internal consistency	Kinaesthetic *α*=0.94^a^, Visual *α*=0.95^a^ Kinaesthetic *α*=0.95^b^, Visual *α*=0.97^b^	Inadequate	?	Test procedure not described. *No information about structural validity of the KVIQ reported. Sample size calculation not mentioned. No information if patients were “stable”. Videorating used for inter-rater reliability could be inappropriate.
					Healthy^b^	24^b^	55.2^b^						
									Inter-rater	Kinaesthetic ICC=0.99 (range 0.99–0.99)^a^ Visual ICC=0.99 (range 0.99–1.00)^a^ Kinaesthetic ICC=0.99 (range 0.99–0.99)^b^ Visual ICC=0.99 (range 0.99–0.99)^b^	Inadequate	+	
									Intra-rater	Kinaesthetic ICC=0.75 (range 0.57–0.86)^a^ Visual ICC=0.87 (range 0.77–0.92)^a^ Kinaesthetic ICC=0.82 (range 0.67–0.91)^b^ Visual ICC=0.90 (range 0.81–0.95)^b^	Inadequate	+	
	n.d.s.	Nakano et al. 2018 [71]	JP	J	Students	28	20.6	13♀, 15♂	Internal consistency	**KVIQ-20/ KVIQ-10** Kinaesthetic *α*=0.91/ *α*=0.77 Visual *α*=0.88/ *α*=0.78	Doubtful	?	Sample size calculation not mentioned and may be insufficient for this analysis. Structural validity of the KVIQ not reported.
Movement Imagery Questionnaire (MIQ)	Sport	Hall et al. 1985 [72]	CA	E	Students	32	NR	NR	Test-retest	Kinaesthetic ICC=0.83 Visual ICC=0.83	Doubtful	+	#, Doubtful sample size.
						80	NR	NR	Internal consistency	Kinaesthetic *α*=0.91 Visual *α*=0.87	Very good	?	Adequate sample size for this analysis but lack of evidence for sufficient structural validity.
	n.d.s.	Atienza & Balaguer 1994 [73]	ES	E	Students	110	20.1	47♀, 63♂	Internal consistency	Kinaesthetic *α*=0.88 Visual *α*=0.89	Very good	?	Very good sample size for this analysis but lack of evidence for sufficient structural validity.
Revised Movement Imagery Questionnaire (MIQ-R)	Sport	Monsma et al. 2009 [74]	USA	E	Athletes and dancers	86	NR	NR	Test-retest	Kinaesthetic 0.81 Visual 0.80	Doubtful	?	Adequate sample size for this analysis. Doubtful how test-retest coefficient was calculated.
						325	20.2	189♀, 136♂	Internal consistency	Kinaesthetic *α*=0.88 Visual *α*=0.84	Very good	+	Very good sample size for this analysis.
Revised Movement Imagery Questionnaire (MIQ-R)	Sport	Williams et al. 2012^1^ [31]	CA	E	Athletes and dancers	400	20.8	219♀, 181♂	Internal consistency	CR=0.82 kinaesthetic and 0.88 visual AVE=0.53 kinaesthetic and 0.65 visual	Very good	+	Williams et al. reported in their article the results of three separate studies. 2012^1^= study 1.
Movement Imagery Questionnaire- Revised second version (MIQ-RS)	Sport	Gregg et al. 2010 [75]	UK	E	Athletes	87	NR	NR	Test-retest	Kinaesthetic *r*=0.73, ICC=0.54–0.73 Visual *r*=0.83, ICC=0.54-0.72	Doubtful	?	MIQ-RS developed for patients with movement limitation and validated in healthy participants.
						321	23.3	174♀, 146♂	Internal consistency	Kinaesthetic *α*=0.90 Visual *α*=0.87	Very good	?	Very good sample size for this analysis but lack of evidence for sufficient structural validity.
	Med	Butler et al. 2012 [76]	USA	E	Stroke^a^	23	59.2	7♀, 16♂	Test-retest	Kinaesthetic (95% CI) ICC=0.92 (0.83–0.97)^a^/ 0.94 (0.86-0.97)^b^ Visual (95% CI) ICC=0.83 (0.64–0.92)^a^/ 0.99 (0.98-0.99)^b^	Doubtful	+	Doubtful sample size and no information if patients were “stable”.
					Healthy^b^	23	51.0	11♀, 12♂					
									Internal consistency	Kinaesthetic T1 *α*=0.97; T2 *α*=0.98 both groups Visual T1 *α*=0.95^a^/ *α*=0.98^b^; T2 *α*=0.95^a^/ 0.98^b^	Doubtful	?	Sample size calculation mentioned based on date from healthy participants, but may be inadequate for this analysis. Lack of evidence for sufficient structural velidity.
	n.d.s.	Loison et al. 2013 [77]	FR	F	Healthy	113	NR	NR	Test-retest	Kinaesthetic ICC=0.78 Visual ICC=0.68	Very good	−	ICC for visual <0.70.
						153	37.9	118♀, 35♂	Internal consistency	*α*=0.90	Inadequate	?	Cronbach’s alpha was reported for total score, not for each subscales
Movement Imagery Questionnaire-3 (MIQ-3)	Sport	Williams et al. 2012^2^ [31]	CA	E	Athletes	370	20.3	185♀, 185♂	Internal consistency	CR=0.83 external, 0.79 internal and 0.85 kinaesthetic AVE=0.55 external, 0.52 internal and 0.59 kinaesthetic	Very good	+	Williams et al. 2012^2^ [31] = results of study 2.
	Sport	Williams et al. 2012^3^ [31]	CA	E	Athletes	97	19.5	58♀, 39♂	Internal consistency	CR=0.89 external, 0.81 internal and 0.89 kinaesthetic AVE=0.66 external, 0.51 internal and 0.67 kinaesthetic	Very good	+	Williams et al. 2012^3^ [31] = results of study 3.
	Sport	Budnik-Przybylska et al. 2016 [78]	PL	PO	Athletes	47	NR	NR	Test-retest	External *r*=0.70 Internal *r*=0.62 Kinaesthetic *r*=0.65	Doubtful	−	Small sample size for this analysis. No information if the participants were stable. 3-weeks interval for the test-retest could explain r <0.70.
						276	21.3	102♀, 174♂	Internal consistency	External *α*=0.75 Internal *α*=0.78 Kinaesthetic *α*=0.81	Very good	+	*Information for sufficient structural validity reported.
	n.d.s.	Paravlic et al. 2018 [79]	Sl	SL	Healthy	80	34.8	40♀, 40♂	Test-retest	External ICC=0.89 (95% CI 0.83-0.93) Internal ICC=0.89 (95% CI 0.82-0.93) Kinaesthetic ICC=0.92 (95% CI 0.87–0.95)	Very good	+	Adequate sample size for this analysis. ICC for each subscales >0.70.
						86	35.3	41♀, 45♂	Internal consistency	External *α*=0.89 Internal *α*=0.89 Kinaesthetic *α*=0.91	Very good	+	Adequate sample size for this analysis.
	n.d.s.	Dilek et al. 2020 [80]	TR	Tu	Healthy	86	NR	NR	Test-retest	External (four items) ICC=range 0.86–0.90 Internal (four items) ICC=range 0.85–0.88 Kinaesthetic (four items) ICC=range 0.86–0.95	Adequate	+	Sample size adequate but test conditions by retest not mentioned.
						181	21.6	53♀, 132♂	Internal consistency	T1: external *α*=0.74, internal *α*=0.74 Kinaesthetic *α*=0.79 T2: external *α*=0.72, internal *α*=0.68 Kinaesthetic *α*=0.74	Very good	+	T1=first test, T2=retest Internal scale at the T2 was <0.70 but that may be considered as sufficient.
Movement Imagery Questionnaire-3 (MIQ-3)	Sport	Robin et al. 2020 [81]	FR	F	Students	172	20.2	115♀	Test-retest	Bravais-Pearson intraclass correlation coefficient External *r*=0.86 Internal *r*=0.87 Kinaesthetic *r*=0.88	Adequate	+	Bravais-Person and not ICC calculated.
							19.9	57♂					
						100	20.4	72♀	Internal consistency	External *α*=0.88 Internal *α*=0.92 Kinaesthetic *α*=0.92	Very good	?	Very good sample size for this analysis. Cronbach's alpha for each scale calculated.
							19.9	28♂					
	n.d.s.	Trapero-Asenjo et al. 2021 [82]	ES	S	Students	62	NR	NR	Test-retest	External ICC=0.81 Internal ICC=0.88 Kinaesthetic ICC=0.82	Adequate	+	Sample size adequate but test conditions for retest not mentioned.
	n.d.s.	Trapero-Asenjo et al. 2021 [82]	ES	S	Students	140	21.5	47♀, 93♂	Internal consistency	External *α*=0.84 Internal *α*=0.85 Kinaesthetic *α*=0.86	Very good	?	Very good sample size, Cronbach's alpha for each scale calculated.
									Measurement error	External SEM=1.47, MDC=4.07 Internal SEM=1.38, MDC=3.82 Kinaesthetic SEM=1.98, MDC=5.48	Adequate	+	Test conditions by retest not mentioned.
Movement Imagery Questionnaire for Children (MIQ-C)	n.d.s.	Martini et al. 2016^1^ [83]	CA	E	Healthy children	20	NR	NR	Development	MIQ-C was developed through adaptions of the MIQ-3. The MIQ-C measures as MIQ-3 external visual, internal visual and kinaesthetic imagery. Cognitive interviews were carried out with children. The interviews were transcribed, reviewed and systematically coded. 12-item MIQ-C was further evaluated.	Doubtful	NA	*Insufficient information about data analysis.
		Martini et al. 2016^2^ [83]	CA	E	Healthy children	23	NR	15♀, 8♂	Test-retest	External ICC=0.43 Internal ICC=0.72 Kinaesthetic ICC=0.82	Doubtful	−	Small sample size for this analysis. ICC external >0.70.
Test of Ability in Movement Imagery (TAMI)	Psy	Madan & Singhal, 2013^2^ [84]	CA	E	Students	24	NR	NR	Test-retest	Pearson’s corr. coefficient *r*=0.71, *p*<0.001	Doubtful	−	Madan & Singhal reported in their article the results of two separate studies. #, Small sample size. ICC no calculated. *Insufficient information for quality criteria rating regarding.
Vividness of Haptic Movement Imagery Questionnaire (VHMIQ)	n.d.s.	Campos et al. 1998 [85]	ES	S	Students	338	20.9	51♀, 287♂	Internal consistency	*α*=0.90	Very good	?	*Insufficient information reported about structural validity of the VMIQ and its modification called VHMIQ.
Vividness of Movement Imagery Questionnaire (VMIQ)	Sport	Isaac et al. 1986 [27]	NZ	E	Students/athletes	220	NR	NR	Test-retest	Pearson’s corr. coefficient *r*=0.76	Doubtful	−	ICC no calculated. *Insufficient information for quality criteria rating.
Vividness of Movement Imagery Questionnaire (VMIQ)	Sport	Eton et al. 1998 [86]	USA	E	Recreational athletes + non-athletes	36	NR	NR	Test-retest	Pearson’s corr. coefficient for internal *r*=0.80, external *r*=0.64	Doubtful	?	Small sample size for this analysis. ICC not calculated. *Insufficient information for quality criteria rating.
					Varsity athletes	51	NR	27♀, 24♂	Internal consistency	External *α*=0.96 Internal *α*=0.96	Very good	?	*Insufficient information for quality criteria rating regarding structural validity.
					Recreational athletes	48		24♀, 24♂					
					Non-athletes	26		14♀, 12♂					
Revised Vividness of Movement Imagery Questionnaire-2 (VMIQ-2)	Sport	Williams et al. 2012^2^ [31]	CA	E	Athletes	370	20.3	185♀, 185♂	Internal consistency	CR=0.94 external, 0.93 internal and 0.93 Kinaesthetic AVE=0.56 external, 0.52 internal and 0.53 kinaesthetic	Very good	+	Very good sample size for this analysis.
	Sport	Williams et al. 2012^3^ [31]	CA	E	Athletes	97	19.5	58♀, 39♂	Internal consistency	CR=0.93 external, 0.92 internal and 0.93 kinaesthetic AVE=0.54 external, 0.50 internal and 0.53 kinaesthetic	Very good	+	Adequate sample size for this analysis.
	Sport	Roberts et al. 2008^3^ [7]	UK	E	Athletes	71	21.72	55♀, 16♂,	Internal consistency	External *α*=0.95 Internal *α*=0.95 Kinaesthetic *α*=0.93	Very good	+	Roberts et al. 2008^3^ [7] = study 3 Adequate sample size for this analysis..
	Sport	Ziv et al. 2017 [87]	IL	HE	Students	88	29.5	56♀,	Test-retest	External *r*=0.72 Internal *r*=0.57 Kinaesthetic *r*=0.66	Doubtful	−	ICC not calculated. *Insufficient information for quality criteria rating
							25.6	32♂,					
									Internal consistency	T1: *α*=0.91 external, *α*=0.95 internal, *α*=0.94 Kinaesthetic T2: *α*=0.94 external, *α*=0.94 internal, *α*=0.95 kinaesthetic	Very good	?	T1=first test, T2= retest. Insufficient information for quality criteria rating regarding structural validity.
	Sport	Qwagzeh et al. 2018 [88]	JO	AR	Students	46	NR	18♀, 28♂,	Internal consistency	External *α*=0.98 Internal *α*=0.98 Kinaesthetic *α*=0.98	Doubtful	?	Sample size calculation not mentioned and may be doubtful for this analysis. Structural validity of the VMIQ-2 not reported
	n.d.s.	Dahm et al. 2019 [89]	AT	G	Students	78	24.0	30♀, 48♂	Test-retest	Concordance correlation coefficient (CCC) calculated External *r*=0.62 Internal *r*=0.61 Kinaesthetic *r*=0.69	Doubtful	−	CCC> 0.70. Doubtful if the test conditions were similar.
						254	24.0	79♀, 175♂	Internal consistency	External *α*=0.91 Internal α=0.90 Kinaesthetic *α*=0.91	Very good	+	Very good sample size for this analysis. Structural validity also reported.
Wheelchair Imagery Ability Questionnaire (WIAQ)	Med	Faull & Jones 2018^1^ [90]	UK	E	Athletes	6	25.17	6♂	Development	All participants (6 athletes and 3 experts) were transcribed verbatim and reviewed and analysed for themes and ideas. 24-item WIAQ was generated by the elite athletes and experts.	Adequate	NA	Results of several studies in this article reported. 2017^1^=study 1. Focus group performed, appropriate data collection method used, data analysis by two authors independently carried out.
					Experts	3	NR	NR					

Legend: The superscript numbers were used to distinguish the results per group

Disciplines in which field the tool was evaluated: *Edu* Education, *Med* Medicine, *Psy* Psychology, *n.d.s.* not discipline-specific healthy participants/students

Country abbreviations: *AT* Austria, *BR* Brazil, *CA* Canada, *CH* Switzerland, *ES* Spain, *FR* France, *JO* Jordan, *IR* Iran, *JP* Japan, *IL* Israel, *SI* Slovenia, *TR* Turkey, *NZ* New Zealand, *PL* Poland, *UK* United Kingdom, *USA* United States of America

Language of the tool: *E* English, *F* French, *G* German, *P* Portuguese, *J* Japanese, *PO* Polish, *SL* Slovenian, *HE* Hebrew, *Tu* Turkish, *S* Spanish, *AR* Arabic

Cronbach’s alpha, *AVE* average variance extracted, *CI* confidence interval, *corr.* correlation, *CR* composite reliability, *COSMIN* Consensus-based Standards for the selection of health Measurement Instruments Risk of Bias Checklist, *external* external perspective, *ICC* interclass correlation coefficient, *internal* internal perspective, *kinaesthetic* kinaesthetic subscale, *KVIQ-20* original Kinaesthetic and Visual Imagery Questionnaire, *KVIQ-10* short version of the KVIQ, *LL* lower limb, *MDC* minimal detectable change, *MS* Multiple Sclerosis, *N* Sample size, *NA* Not applicable, *NR* Not reported, *PD* Parkinson disease, *SEM* standard error of measurement, *visual* visual subscale

# methods could be doubtful, students received a course credits for participation. It could be interpreted that there was a certain dependency/necessity to participate, but it was not taken into account by the COSMIN evaluation

Quality Criteria: ‘+’ = sufficient, ‘−’ = insufficient, ‘?’ = indeterminate, *See Table 1 and Legend for explanation of quality criteria

**Table 4:** Motor imagery assessments: The characteristics of the included studies - Validity

Tool	Disciplines	Study	Country	Language	Study population				Validity		COSMIN	Quality criteria	Comments
					Participants	***N***	Age mean (years)	Sex	Design	Results			
Kinesthetic and Visual Imagery Questionnaire (KVIQ)	Med	Malouin et al. 2007 [43]	CA	E	Stroke^a^	33	60.1	7♀, 26♂	Construct validity- structural validity	**KVIQ-20 + KVIQ-10** PCA and oblique rotation extracted two factors for both versions. Correlation between the two factors for both versions was 0.46. Factor loadings for KVIQ-20 ranged from 0.70 to 0.88 (visual) and 0.68 to 0.80 (kinaesthetic); for KVIQ-10 ranged from 0.73 to 0.86 (visual) and 0.68 to 0.80 (kinaesthetic). Total variance explained by 63.4% for KVIQ-20 and 67.7% for KVIQ-10	Adequate	+	EFA applied, factors loading >0.40, variance explained less than 50%, corr. among factors reported.
					Healthy^b^	70	42.9	49♀, 21♂					
					LL amputation^c^	13	35.0	13♂					
					Acquired blindness^d^	10	40.8	4♀, 6♂					
					LL immobilization^e^	5	50.1	5♂					
	Med	Randhawa et al. 2010 [68]	CA	E	PD	11	61.7	7♀, 4♂	Construct validity- hypothesis testing	**Corr. KVIQ-20 and MIQ-R** *r*=0.94 kinaesthetic *r*=0.88 visual *r*=0.93 for total score	Inadequate	+	Sample size included in this analysis not adequate. Strong corr. with instruments measuring the same construct.
	Med	Schuster et al. 2012 [67]	CH	G	Subacute stroke Chronic stroke Left parietal lobe MS PD	19	59.9	6♀, 13♂	Construct validity- hypothesis testing	**Corr. KVIQ-G and Imaprax-G** *r*=0.36 visual (KVIQ-G-20 vs. Imaprax) *r*=0.32 visual (KVIQ-G-10 vs. Imaprax)	Doubtful	−	Small sample size. Only patients, who chose the internal perspective, were analysed. Low corr. with instruments measuring the same construct.
						73	62.8	28♀, 45♂	Construct validity- structural validity	**KVIQ-G-20** PCA and promax rotation identified bifactorial structure of the KVIQ-G-20. Factor loadings for kinaesthetic subscale 0.79–0.93 and 0.68–0.91 for visual. Total variance of both factors explained by 69.7%	Inadequate	?	EFA applied, factors loading >0.40, variance explained less than 50%, corr. among factors reported but very low sample size.
	Med	Tabrizi et al. 2013 [69]	IR	NR	MS	15	31.7	12♀, 3♂	Construct validity- hypothesis testing	**Corr. KVIQ-20 and MIQ** *r*=0.75 kinaesthetic *r*=0.78 visual	Doubtful	+	*Insufficient information about factor analysis reported for quality criteria rating. Strong corr. with instruments measuring the same construct.
									Construct validity- structural validity	**KVIQ-20** Bifactorial structure of the KVIQ-20 was confirmed. Total variance of both factors explained by 90%	Inadequate	?	
	Med	Nakano et al. 2018 [71]	JP	J	Students	28	20.6	13♀, 15♂	Construct validity- hypothesis testing	**Corr. KVIQ-20 and MIQ-R** *r*=0.77 kinaesthetic *r*=0.64 visual **Corr. KVIQ-10 and MIQ-R** *r*=0.78 kinaesthetic *r*=0.62 visual	Doubtful	+	Sample size calculation not mentioned. Small sample size. Strong corr. with instruments measuring the same construct.
Movement Imagery Questionnaire (MIQ)	Sport	Hall et al. 1985 [72]	CA	E	Students	80	NR	NR	Construct validity- stability of the internal structure	**Corr. kinaesthetic vs. visual subscale** Correlation between the score achieved on the both subscales (kinaesthetic and visual) was 0.58	NA	NA	Factor structure was not analysed. Only the total score corr. for both subscales was reported and authors suggest the stability of the subscale structure.
	n.d.s	Atienza & Balaguer 1994 [73]	ES	E	Students	110	20.1	47♀, 63♂	Construct validity- structural validity	Common factor analysis using maximum likelihood and oblique rotation confirmed extracted two factors. Factor loadings for visual ranged from 0.58 to 0.82 and for kinaesthetic 0.46 to 0.81. Total variance explained by 47.8%.	adequate	?	Explained variance <50%, but all factors loaded >0.40. Corr. among factors not reported.
	n.d.s	Lequerica et al. 2002 [22]	USA	E	Students	80	22.1	41♀, 39♂	Construct validity- hypothesis testing	**Corr. MIQ and GTVIC** *r*=0.45 MIQ visual **Corr. MIQ and VMIQ** *r*=0.56 kinaesthetic; *r*=0.52 visual	Doubtful	+	#, Insufficient information on measurement properties of the comparator measures. The results in accordance with hypothesis: sign. corr. among subjective measures of mental imagery. No corr. between subjective and objective measures of mental imagery ability providing the evidence for the multidimensional nature of imagery.
Revised Movement Imagery Questionnaire (MIQ-R)	Psy	Hall & Martin 1997 [91]	CA	E	Students	50	20.9	26♀, 24♂	Criterion validity	**Corr. MIQ and MIQ-R** *r*=0.77 kinaesthetic *r*=0.77 visual	Doubtful	+	#, Doubtful sample size. Corr. with gold standard- MIQ was >0.70.
	Sport	Monsma et al. 2009 [74]	USA	E	Athletes and dancers	325	20.2	189♀, 136♂	Construct validity- structural validity	CFA include a path between two factors (kinaesthetic and visual) and suggest these two factors are interrelated. ∆*χ*^2^(1)=126.14, *p*<0.001. CFI=0.99, NNFI=0.98, AGFI=0.95, SRMR=0.03, RMSEA=0.06.	Very good	+	Accepted model fit: CFI, NNFI or AGFI >0.95, or SRMR <0.08, or RMSEA <0.06.
	Sport	Williams et al. 2012^1^ [31]	CA	E	Athletes and dancers	400	20.8	219♀, 181♂	Construct validity- structural validity	MTMM approach to CFA and two models CT and CTCU were tested. Factor loadings for both models ranged from 0.70- 0.84. Corr. between the two factors (kinaesthetic and visual) for the CT was 0.25 and for the CTCU 0.23. CTCU model provided a significantly better fit to the data compared with the CT model. *χ*^2^=25.99, df=15, CFI=0.99, TLI=0.99, SRMR=0.03, RMSEA=0.05. The kinaesthetic and visual imagery are separate but related constructs.	Very good	+	Accepted model fit: CFI, TLI>0.95, or SRMR <0.08, or RMSEA<0.06.
Movement Imagery Questionnaire- Revised second version (MIQ-RS)	Sport	Gregg et al. 2010 [75]	UK	E	Athletes	321	23.3	174♀, 146♂	Construct validity- structural validity	CFA confirmed the bifactorial (kinaesthetic and visual) structure of MIQ-RS. *χ*^2/^/df=3.72, CFI=0.99, RFI=0.98, RMSEA=0.09.	Inadequate	-	MIQ-RS developed for patients with motor impairments but tested with athletes. Should be tested in another field. RMSEA not acceptable. SRMR not reported.
									Criterion validity	**Corr. MIQ-RS and MIQ-R** *r*=0.80 kinaesthetic *r*=0.82 visual	Very good	+	Corr. with gold standard- MIQ-R was >0.70.
	Med	Butler et al. 2012 [76]	USA	E	Stroke^a^	23	59.2	7♀, 16♂	Construct validity- structural validity	PCA and varimax rotation extracted two factors: kinaesthetic and visual. Communalities ranged from 0.72 to 0.95 in the stroke and 0.72 to 0.96 in the healthy group. Corr. between the two factors (kinaesthetic and visual) in the stroke was 0.61 and in the healthy 0.69. Total variance in the stroke group was explained by 83.4% and in the healthy group by 88.6%.	Inadequate	?	All criteria for EFA fulfilled but very low sample size.
					Healthy^b^	23	51	11♀, 12♂					
									Construct validity- hypothesis testing	**Corr. MIQ-RS and KVIQ-10** kinaesthetic *r*=0.84^a^/ *r*=0.86^b^ visual *r*=0.62^a^/ 0.77^b^	Very good	+	Strong corr. with instruments measuring the same construct.
	n.d.s.	Loison et al. 2013 [77]	FR	F	Healthy	153	37.9	118♀, 35♂	Construct validity- structural validity	CFA confirmed the bifactorial (kinaesthetic and visual) structure of MIQ-RS French version. Corr. between items were strong, for the kinesthetic 0.74–0.85 and for visual 0.65–0.79. Total variance explained by 55–73% for kinesthetic and 42–62% for visual. *χ*^2/^/df=2.23, CFI=0.93, SRMR=0.06, RMSEA=0.09.	Very good	−	Accepted model fit: CFI >0.95, or SRMR <0.08, or RMSEA <0.06.
Movement Imagery Questionnaire-3 (MIQ-3)	Sport	Williams et al. 2012^2^ [31]	CA	E	Athletes and dancers	370	20.3	185♀, 185♂	Construct validity- structural validity	MTMM approach to CFA and two models CT and CTCU were tested. Factor loadings for the CT model ranged from 0.70 to 0.81 and for the CTCU model ranged from 0.64 to 0.81. Corr. between the factors (external, internal and kinesthetic) for the CT was 0.33 to 0.68 and for the CTCU 0.32 to 0.60. The three-factor CTCU model provided the best fit to the data compared with the CT model: *χ*^2^=75.12, df=39, CFI=0.98, TLI=0.97, SRMR=0.04, RMSEA=0.05. The corr. between kinaesthetic and internal was strong (*r* = 0.60)	Very good	+	Accepted model fit: CFI or TLI >0.95, or SRMR <0.08, or RMSEA <0.06 The MIQ-3 factor structure was not invariant across gender.
									Criterion validity- concurrent validity	**Corr. MIQ-3 and VMIQ-2** *r*=0.68 external *r*=0.63 internal *r*=0.71 kinaesthetic	Very good	-	Corr. between MIQ-3 and VMIQ-2 only for kinaesthetic just above 0.70.
	Sport	Williams et al. 2012^3^ [31]	CA	E	Athletes	97	19.5	58♀, 39♂	Criterion validity- Predictive validity	MIQ-3 external sign. predict skill observational learning (OL) *β*=0.39, *t*=2.82, *p*=0.006 MIQ-3 external sign. predict strategy (OL) *β*=0.44, *t*=3.17, *p*=0.002 MIQ-3 kinaesthetic sign. predict performance (OL) *β*=0.48, *t*=3.30, *p*=0.001	Doubtful	?	Multiple regressions conducted to assess the predictive validity. Sample size doubtful. Doubtful if FOLO could be used as external criterion.
	Sport	Budnik-Przybylska et al. 2016 [78]	PL	PO	Athletes	276	21.3	102♀, 174♂	Construct validity- structural validity	CFA with maximum likehood estimation confirmed the three-factor (external, internal and kinaesthetic) structure. *χ*^2^=76.98, df=51, CFI=0.93, GFI=0.89, AGFI=0.83, RMR=0.25, RMSEA=0.04	Very good	+	Accepted model fit: CFI, GFI >0.95, or SRMR <0.08, or RMSEA <0.06.
	n.d.s.	Paravlic et al. 2018 [79]	Sl	SL	Healthy	86	35.3	41♀, 45♂	Construct validity- structural validity	CFA and three-factor model achieved best model fits: *χ*^2^=75.40, df=51, CFI=0.94, TLI=0.93, RMR/SRMR=0.11, RMSEA=0.07	Adequate	−	Accepted model fit: CFI or TLI >0.95, or SRMR <0.08, or RMSEA <0.06. Above mentioned criteria for good properties not met.
	n.d.s.	Dilek et al. 2020 [80]	TR	Tu	Healthy	181	21.6	53♀, 132♂	Construct validity- structural validity	CFA and the three-factor structures previously proposed in the literature were tested using the LISREL structural equation-modelling programme developed. *χ*^2^ =115.60, df =51, *P*=0.000). CFI=0.97, GFI=0.91, AGFI=0.86, RMR=0.04, RMSEA=0.08, SRMR=0.05 Factor loadings 0.54–0.76.	Very good	+	Accepted model fit: CFI or GFI >0.95, or SRMR <0.08, or RMSEA <0.06.
	Sport	Robin et al. 2020 [81]	FR	F	Students	172	20.2	115♀	Construct validity- structural validity	EFA identified three factors: external, internal and kinaesthetic. Explained variance by factor 1=48.63%, factor 2=14.56%, factor 3=17.71%. Factor loadings 0.74–0.92. CFA with maximum likelihood was performed: *χ*^2^=120.75, df=54, CFI=0.91, RMSR=0.07 and 0.08, RMSEA=0.09.	Very good	−	Accepted model fit: CFI or GFI >0.95, or SRMR <0.08, or RMSEA <0.06.
							19.9	57♂					
	n.d.s.	Trapero-Asenjo et al. 2021 [82]	ES	S	Students	140	21.5	47♀, 93♂	Construct validity- structural validity	CFA and the three-factor model showed good fit: RMSEA=0.07, NFI=0.90, RFI=0.91, CFI=0.90. The absolute fit measures with *χ*^2^ of *p*=0.001 indicating an inadequate model.	Doubtful	-	Accepted model fit: CFI or GFI >0.95, or SRMR <0.08, or RMSEA <0.06. Rotation method by CFA not described.
									Construct validity- hypothesis testing	**Corr. MIQ-3 and MIQ-R** Total score Spearmen’s *r*=0.89 External and visual *r*=0.72 Internal and visual *r*=0.70 Kinaesthetic scales *r*=0.89	Inadequate	+	No information on the measurement properties of the comparator instrument. Strong corr. with instruments measuring the same construct.
Movement Imagery Questionnaire for Children (MIQ-C)	n.d.s.	Martini et al. 2016 [83]	CA	E	Healthy children	204	9.6	125♀, 79♂	Construct validity- structural validity	MTMM approach to CFA and four models were tested. Factor loadings for the CT model ranged from 0.51 to 0.67 and for the CTCU model ranged from 0.51 to 0.69. Corr. between the factors (external, internal and kinaesthetic) for the CT was 0.42 to 0.65 and for the CTCU 0.39 to 0.63. The three-factor CTCU model provided the best fit to the data compared with the CT model: *χ*^2^=75.33, df=39, CFI=0.93, TLI=0.89, SRMR=0.05, RMSEA=0.07.	Very good	+	Accepted model fit: CFI or TLI >0.95, or SRMR <0.08, or RMSEA <0.06.
Test of Ability in Movement Imagery (TAMI)	Psy	Madan & Singhal, 2013^2^ [84]	CA	E	Students	49	19.6	29♀, 20♂	Construct validity- structural validity	PCA and varimax rotation confirmed that factor objective movement imagery was loaded by TAMI with 0.81.	Inadequate	?	#, EFA performed but not explicit to explore the structural validity of TAMI. *Insufficient information reported for quality criteria rating.
									Construct validity- hypothesis testing	**Corr. TAMI and VMIQ-2 internal visual:** *r*=0.36, *p*<0.05 **Corr. TAMI and FPIQ three subscales: ***r*=0.45^1^, *r*=0.39^2^, *r*=0.34^3^, *p*<0.05 **Corr. TAMI and VVIQ:***r*=0.43, *p*<0.01 TAMI do not correlate with VMIQ-2 external and kinaesthetic subscales, with the MRT, and with the FPIQ kinaesthetic	Inadequate	?	The subscales of FPIQ: ^1^= position, ^2^= action, ^3^= object No hypothesis defined. Insufficient information about measurement properties of the comparator instrument.
	Psy	Madan & Singhal, 2014 [92]	CA	E	Students	189	19.5	125♀, 64♂	Construct validity- hypothesis testing	Corr. TAMIw *and VMIQ-2 internal visual: *r*=0.37 **Corr. TAMIw and FPIQ subscale position: ***r*=0.44 **Corr. TAMIw and VVIQ: ***r*=0.32 TAMIw does not correlate with VMIQ-2 external and kinaesthetic subscales, with the MRT, and with the FPIQ action, object and kinaesthetic subscales	Inadequate	?	#, No hypothesis defined. No information about measurement properties of the comparator instrument.
Test of Ability in Movement Imagery with Hands (TAMI-H)	Psy	Donoff et al. 2017 [93]	CA	E	Students	70	NR	49♀, 21♂	Construct validity- hypothesis testing	**Corr. TAMI-H and TAMIw:** *r*=0.29 FM/ *r*=0.53 IM **Corr. TAMI-H and FPIQ kinaesthetic:** *r*=0.34 FM/ *r*=0.26 IM **Corr. TAMI-H and FPIQ position:** *r*=0.19 FM/ *r*=0.26 IM **Corr. TAMI-H and FPIQ action:** *r*=0.21 FM/ *r*=0.34 IM **Corr. TAMI-H and FPIQ object:** *r*=0.35 FM/ r=0.44	inadequate	?	Author mentioned that new Tool-TAMI-H (with two imagery type: Functionally-involved Movement (FM) and Isolated Movement (IM)) was developed but no information reported about development. Measurement properties of the comparator instrument not mentioned.
Vividness of Haptic Movement Imagery Questionnaire (VHMIQ)	n.d.s.	Campos et al. 1998 [85]	ES	S	Students	338	20.9	51♀, 287♂	Construct validity- hypothesis testing	**Corr. VHMIQ and VMIQ** Pearson *r*=0.56 for women, *r*=0.66 for men 0.66 and *r*=0.60 for all participants.	Inadequate	?	Strong corr. was expected. Not reported if different corr. between VHMIQ and internal VMIQ or VHMIQ and external VMIQ was found. No information about measurement properties of the comparator instrument.
									Construct validity- hypothesis testing	**Known-groups validity** Mixes-model analysis of variance with the factor sex and type of image: neither sex (F: 2.12 *p*>0.05) or type of image (F: 3.24, *p*>0.05) had a sig. effect on reported vividness of imagery.	Doubtful	?	Results are in accordance with the hypothesis that no sex difference should be expected but no adequate description provided of important characteristics of the subgroups.
Vividness of Movement Imagery Questionnaire (VMIQ)	Sport	Isaac et al. 1986 [27]	NZ	E	Students^a^	220	NR	NR	Construct validity- hypothesis testing	**Corr. VMIQ and VVIQ** Pearson corr. coefficient for group a *r*=0.81 Spearman rank for group b *r*=0.75, group c *r*=0.45 and group d *r*=0.65	Inadequate	?	Small sample size in group b, c and d. Corr. ranged from low to strong among different groups. But group differences not reported. Insufficient information about measurement properties of the comparator instrument.
					No trampoline experience^b^	25							
					Trampoline experience^c^	25							
					International level trampolinists^d^	16							
	Sport	Eton et al. 1998 [86]	USA	E	Varsity athletes	51	NR	27♀, 24♂	Construct validity- hypothesis testing	**Corr. VMIQ and VVIQ** *r*=0.60, *p*<0.01	Doubtful	?	Doubtful if constructs measured by comparator instrument are same. Some information about measurement properties of the comparator instrument.
					Recreational athletes	48		24♀, 24♂					
					Non-athletes	26		14♀, 12♂					
	n.d.s	Lequerica et al. 2002 [22]	USA	E	Students	80	22.1	41♀, 39♂	Construct validity- hypothesis testing	**Corr. VMIQ and GTVIC** *r*=0.72 VMIQ visual **Corr. VMIQ and MIQ** see above notes for the MIQ	Doubtful	+	See above comments for the MIQ.
Revised Version of the Vividness of Movement Imagery Questionnaire (VMIQ-2)	Sport	Roberts et al. 2008^1^ [7]	UK	E	Athletes	351	20.44	159♀, 189♂	Construct validity- structural validity	The three-factor CTCU analysis provided the best fit to the data: *χ*^2^=840.65, df=555, CFI=0.98, NNFI=0.97, SRMR=0.04, RMSEA=0.04. Factor loadings ranged from 0.60 to 0.78. Corr. between the factors: internal and external *r*=0.39, internal and kinaesthetic *r*=0.63, external and kinaesthetic *r*=0.41	Very good	+	Roberts et al. reported in their article the results of three separate studies. 2008^1^= study 1 Very good sample size for this analysis.
	Sport	Roberts et al. 2008^2^ [7]	UK	E	Athletes	355	20.44	119♀, 235♂, 1 NR	Construct validity- structural validity	The three-factor CTCU further provided the best fit to the data: *χ*^2^=1242.76, df=555, CFI=0.98, NNFI=0.97, SRMR=0.06, RMSEA=0.06. Factor loadings ranged from 0.64 to 0.82. Corr. between the factors: internal and external r=0.51, internal and kinaesthetic r=0.62, external and kinaesthetic r=0.43	Very good	+	Roberts et al. 2008^2^ [7]= study 2 Very good sample size for this analysis.
	Sport	Roberts et al. 2008^3^ [7]	UK	E	Athletes	71	21.72	55♀, 16♂,	Construct validity- hypothesis testing	**Corr. internal VMIQ-2 and visual MIQ-R** *r*=−0.34, *p*<0.05 **Corr. external VMIQ-2 and visual MIQ-R** *r*=−0.65, *p*<0.01 **Corr. kinaesthetic VMIQ-2 and kinaesthetic MIQ-R** *r*=−0.74, *p*<0.01	Doubtful	+	Roberts et al. 2008^3^ [7]= study 3 Strong corr. with instruments measuring the same construct. 75% of the results are in accordance with the hypotheses.
	Sport	Qwagzeh et al. 2018 [88]	JO	AR	Students	46	NR	18♀, 28♂,	Construct validity- hypothesis testing	Concurrent validity was 0.89.	Inadequate	−	No information about comparator or how concurrent validity was calculated. Only briefly mention in the text.
									Construct validity- hypothesis testing/	**Known-groups validity** There were gender differences: female demonstrated more clear and vivid external imagery (*p*<0.001) and kinaesthetic (*p*<0.001) than male. For internal imagery no sign. differences (*p*=0.339) were found.	Inadequate	?	No adequate description provided of important characteristics of the subgroups for understanding of these results. No difference was expected.
	n.d.s.	Dahm et al. 2019 [89]	AT	G	Students	254	24.0	79♀, 175♂	Construct validity- structural validity	MTMM and MT approach to CFA and three models were tested. The three-factor MTMM model provided the best fit to the data: *χ*^2^/df=1.63, CFI=0.92, SRMR=0.06, RMSEA=0.05. Factor loadings for external 0.57–0.75, for internal 0.56–0.73, for kinaesthetic 0.60–0.74.	Very good	+	Accepted model fit: CFI or TLI >0.95, or SRMR <0.08, or RMSEA <0.06. Not all criteria met for positive rating of this measurement property.
Wheelchair Imagery Ability Questionnaire (WIAQ)	Med	Faull & Jones 2018^2^ [90]	UK	E	Athletes	115	31.46	62♀, 53♂	Construct validity- structural validity	CFA using maximum likelihood was performed. The three-factor 15-item model was tested using the three Bayesian Structural Equation Modelling. The interfactor correlations between the three imagery factors were as follows; external with internal *r*=0.71 (0.59, 0.80), external with kinaesthetic *r*=0.48 (0.30, 0.63), and internal with kinaesthetic *r*=0.63 (0.49, 0.74).	Doubtful	?	Sample size was adequate. 2017^2^= study 2. The use of BSEM analysis is becoming accepted as an innovative method to analyse a structural validity. However, this method was not proposed by COSMIN and therefore our rating is doubtful and indeterminate for this measures.
	Med	Faull & Jones 2018^3^ [90]	UK	E	Athletes	115	31.46	62♀, 53♂	Construct validity- hypothesis testing	**Corr. WIAQ with SIAQ (total score)** external and SIAQ *r*=0.39 internal and SIAQ *r*= 0.26 kinaesthetic and SIAQ *r*=0.20 **Corr. WIAQ and TOPS-2 (two scales, practice and competition)** external and practice *r*=0.23, external and competition *r*=0.27 kinaesthetic and practice *r*=0.21, kinaesthetic and competition *r*=0.27 No sig. corr. between internal and TOPS-2	Doubtful	+	2017^3^= study 3. No information about measurement properties of the comparator instrument. 75% of the results are in accordance with the hypotheses.

Legend: The superscript numbers were used to distinguish the results per group

*Disciplines* in which field the tool was evaluated: *Edu* education,* Med* medicine, *Psy* psychology, *n.d.s.* not discipline-specific; healthy participants/students

Country abbreviations: *AT* Austria, *CA* Canada, *CH* Switzerland, *ES* Spain, *FR* France, *JO* Jordan, *IR* Iran, *JP* Japan, *SI* Slovenia, *TR* Turkey, *NZ* New Zealand, *PL* Poland, *UK* United Kingdom, *USA* United States of America

Language of the tool: *E* English, *F* French, *G* German, *P* Portuguese, *J* Japanese, *PO* Polish, *SL* Slovenian, *Tu* Turkish, *S* Spanish, *AR* Arabic

*AGFI* adjusted goodness of fit index, *BSEM* Bayesian Structural Equation Modeling, *CI* confidence interval, *corr.* correlation, *CT* correlated trial, *CFA* confirmatory factor analysis, *CFI* Comparative fit index, *CTCU* correlated trial-correlated uniqueness, *COSMIN* Consensus-based Standards for the selection of health Measurement Instruments Risk of Bias Checklist, *df *degrees of freedom, *EFA* exploratory factor analysis, external external perspective subscale, FOLO Functions of Observational Learning Questionnaire, FPIQ Florida Praxis Imagery Questionnaire, GFI goodness of fit index, *GTVIC* Gordon Test of Visual Imagery Control, *internal* internal perspective subscale, *kinaesthetic* kinaesthetic subscale, *KVIQ-20* original Kinaesthetic and Visual Imagery Questionnaire, *KVIQ-10* short version of the KVIQ, *MS* multiple sclerosis, *LISREL* Linear Structural RELations, *MT* Multi-Trait, *MRT* Mental Rotation Test, *MTMM* Multitrait-multimethod, *N* sample size, *NR* not reported, *NNFI* non-normed fit index, *PCA* principal component analysis, *PD* Parkinson disease, *RFI* Relative Fit Index, *RMR* the root mean square residual, *RMSEA* root mean square error of approximation, sign. significant, *SIAQ* Sport Imagery Ability Questionnaire, *SRMR* standardised root mean square residual, *TLI* Tucker-Lewis index, *TAMI* Test of Ability in Movement Imagery, *TAMI-H* Test of Ability in Movement Imagery with Hands, TAMIw* TAMI-weighted - new scoring method (More difficult questions were more weighted than relatively easier questions), *TOPS-2* Test of Performance Strategies-2, visual visual subscale, *VVIQ* Vividness of Visual Imagery Questionnaire, *χ2* chi-square;

# methods could be doubtful, students received a course credits for participation. It could be interpreted that there was a certain dependency/necessity to participate, but it was not taken into account by the COSMIN evaluation

Quality Criteria: ‘+’ =sufficient, ‘−’=insufficient, ‘?’=indeterminate. *See Table 1 Legend for explanation of quality criteria

For criteria of an exploratory factor analysis (EFA) see de Vet et al. 2011 [52], Izquierdo et al. 2014 [61] and Watkins 2018 [62]

### Motor imagery assessments: validity

#### Risk of bias rating

In total, 30 out of the 33 motor imagery articles reported structural, criterion or construct validity. Only ten studies [6, 43, 73, 74, 77–80, 83, 89] were rated as very good or adequate and 12 studies [27, 67–69, 75, 76, 82, 84, 85, 88, 92, 93] were rated as inadequate regarding their methodological quality. The ‘risk of bias assessment/rating’ could not be applied to the study by Hall et al. [72] due to insufficient reporting on statistical methods that were performed.

### Measurement properties

There is high evidence for sufficient structural validity regarding the MIQ-R, MIQ-3 and VMIQ-2 assessments. The MIQ-C showed also sufficient structural validity but with moderate evidence (only one study of very good methodological quality). Construct validity of the MIQ and WIAQ was sufficient, but with low evidence (one study per assessment with doubtful quality). The FPIQ and Imaprax were not evaluated for validity. Further, the structural and construct validity of the KVIQ (original and short versions) for different language versions ranged from insufficient to sufficient between studies. These psychometric properties were evaluated with different populations (e.g. healthy individuals, patients after a stroke, Parkinson’s disease (PD), multiple sclerosis (MS), or patients with orthopaedic problems). However, only one study per subgroup was identified, which meant that pooling the data was not feasible. Furthermore, the construct validity of the KVIQ was sufficient in two studies (with PD or with MS patients), but both studies had a very small sample size (*N* < 15) and were therefore downgraded for imprecision. Moreover, structural and construct validity of the MIQ-RS, TAMI, TAMI-H and VMIQ reported in several studies were rated as indeterminate.

### Motor imagery assessments: Reliability

#### Risk of bias rating

In total, 29 out of the 33 motor imagery articles reported development, internal consistency or test-retest reliability. Nine studies [7, 31, 73, 79–82, 85, 90] were rated as very good or adequate regarding their methodological quality. A total of 15 studies [27, 43, 67, 71, 72, 74–76, 78, 83, 84, 86–89] showed doubtful methodological quality and five studies [66, 68–70, 77] were rated as inadequate.

#### Measurement properties

The test-retest reliability of several assessments was insufficient or indeterminate due to a lack of details reported in the studies, e.g. how reliability was calculated. For example, authors of several studies did not calculate the intraclass correlation coefficient (ICC) and stated that a ‘reliability coefficient’ or ‘reliabilities’ were calculated without specific description on the types of coefficients that were calculated (e.g. ICC, Pearson or Spearman correlations). In most cases, internal consistency was insufficient or indeterminate due to low evidence for sufficient structural validity. Only the MIQ-R, MIQ-3 and VMIQ-2 revealed a very clear sufficient internal consistency with a high evidence (multiple studies of at least adequate methodological quality) which corresponds to a sufficient structural validity. The KVIQ showed sufficient test-retest reliability but with low evidence. However, the results were summarised only for patients after a stroke.

Only two studies [76, 83] reported a sample size calculation. For the MIQ, MIQ-R, MIQ-3, VMIQ, VMIQ-2, KVIQ, and TAMI, the results were qualitatively summarised and reported in the Summary of Findings (SoF) Table (Additional file 4: Table 2S).

### Mental imagery assessments

In total, 90 out of 121 articles reported mental imagery assessments. Based on their construct, we divided the assessments into three subgroups:

1. General mental imagery ability assessments (*n* = 24): Auditory Imagery Scale (AIS), Auditory Imagery Questionnaire (AIQ), Bucknell Auditory Imagery Scale (BAIS), Betts Questionnaire Upon Mental Imagery (150 items, QMI), Betts Questionnaire Upon Mental Imagery (shorted 35 items, SQMI), Clarity of Auditory Imagery Scale (CAIS), Gordon Test of Visual Imagery Control (TVIC), Imaging Ability Questionnaire (IAQ), Imagery Questionnaire by Lane, Kids Imaging Ability Questionnaire (KIAQ), Mental Imagery Scale (MIS), Plymouth sensory imagery Questionnaire (Psi-Q), Sport Imagery Ability Measure (SIAM), Revised Sport Imagery Ability Measure (SIAM-R), Sport Imagery Ability Questionnaire (SIAQ), Survey of mental imagery, Visual Elaboration Scale (VES), Vividness of Olfactory Imagery Questionnaire (VOIQ), Vividness of Object and Spatial Imagery Questionnaire (VOSI), Vividness of Visual Imagery Questionnaire (VVIQ), Revised version Vividness of Visual Imagery Questionnaire (VVIQ-2), Vividness of Visual Imagery Questionnaire- Revised version (VVIQ-RV), Vividness of Visual Imagery Questionnaire-Modified (VVIQ-M), Vividness of Wine Imagery Questionnaire (VWIQ).
2. Assessments to evaluate ability to rotate or manipulate mental images- mental rotation (*n* = 12): Card Rotation Test, Cube-cutting Task (CCT), German Test of the Controllability of Motor Imagery (TKBV), Hand laterality task, Judgement test of foot and trunk laterality, Map Rotation Ability Test (MRAT), Mental Paper Folding (MPF), Mental Rotation of Three-Dimensional Objects, Measure of the Ability to Form Spatial Mental Imagery (MASMI), Measure of the Ability to Rotate Mental Images (MARMI), Shoulder specific left right judgement task (LRJT), Spatial Orientation Skills Test (SOST).
3. Assessments of mental imagery to distinguish between the use of different cognitive styles (*n* = 7): Object-Spatial Imagery Questionnaire (OSIQ), Object-Spatial Imagery and Verbal Questionnaire (OSVIQ), Paivio’s Individual Differences Questionnaire (3 IDQ versions with 86 items, 72 items and 34 items), Sussex Cognitive Styles Questionnaire (SCSQ), Verbalizer-Visualizer Questionnaire (VVQ).
4. Assessments to evaluate use of imagery (*n* = 5): Children’s Active Play Imagery Questionnaire (CAPIQ), Exercise Imagery Questionnaire - Aerobic Version (EIQ-AV), Sport Imagery Questionnaire (SIQ), Sport Imagery Questionnaire for Children (SIQ-C), Spontaneus Use of Imagery Scale (SUIS).

Tables 5 and 6 present the characteristics of included studies, the ‘risk of bias assessment/rating’ and the psychometric properties. The general characteristics of included instruments as well as SoF are presented in Additional files 5 and 6: Tables 3S and 4S.

**Table 5:** Mental imagery assessments: The characteristics of the included studies - Reliability

Tool	Disciplines	Study	Country	Language	Study population				Reliability				COSMIN	Quality criteria	Comments
					Participants	***N***	Age mean (years)	Sex	Design	Results					
**a. General mental imagery in any sensorial modality**															
Auditory Imagery Scale (AIS)	n.d.s.	Gissurarson 1992 [94]	IS	E	Volunteers	160	33.0	70♀, 90♂	Internal consistency	*α*=0.80			Very good	?	Very good sample size. Cronbach's alpha >0.70. Structural validity reported but indeterminate.
	n.d.s.	Campos 2017 [95]	ES	S	Students	444	20.4	190♀, 254♂	Internal consistency	*α*=0.63			Very good	−	Very good sample size. Cronbach’s alpha <0.70.
Auditory Imagery Questionnaire (AIQ)	n.d.s.	Hishitani 2009^1^ [160]	JP	E	Students	10	21.8	10♂	Development	Students were recruited for item collection. 12 items were selected, and each item can be rated on a 5-point scale.			Inadequate	NA	It is not clear, for which target population the AIQ was developed. Data collection and analysis not described.
	n.d.s.	Campos 2017 [95]	ES	S	Students	444	20.4	190♀, 254♂	Internal consistency	*α*=0.74			Very good	+	Very good sample size. Cronbach’s alpha >0.70. Structural validity reported.
Bucknell Auditory Imagery Scale (BAIS)	n.d.s.	Halpern 2015 [97]	USA	E	Volunteers	76	22.6	22♀, 54♂	Internal consistency	Control scale *α*=0.81 vividness scale *α*=0.83			Very good	?	Cronbach's alpha for both scales calculated and >0.70. Structural validity reported but indeterminate.
Betts Questionnaire Upon Mental Imagery (original 150-item, QMI)	Psy	Betts 1909 [25]	CO	E	Students and psychologists	46	NR	NR	Development	Betts described 4 experiments with 143 participants. 1 experiment (*n*=46) was development of QUMI. 7 sensory modalities were defined: visual, auditory, cutaneous, kinaesthetic, gustatory, olfactory, organic with total 150 items, and rating scale 1-7. In another experiments the degree of clearness and vividness of the image, the correlation of the various type of image with each other and the correlation of imagery ability with scholarly was studied with students and teachers.			NA	NA	Development of QMI but no psychometric properties reported. No information provided about the target population for which the assessment was developed.
Betts Questionnaire Upon Mental Imagery (shorted version 35-item, SQMI)	Psy	Sheehan 1967 [98]	AU	E	Students	280	23.0	140♀, 140♂	Development	7 sensory modalities: visual, auditory, cutaneous, kinaesthetic, gustatory, olfactory and organic. Total 35 items.			Inadequate	NA	Betts and Sheehen included psychology students for evaluation. Further studies are needed including older populations.
	n.d.s.	Sheehan 1967 [98]	USA	E	Students	62	NR	62♀	Test-retest	Pearson corr. visual subscale and total score *r*=0.78.			Inadequate	−	Time interval (7 months) for test-retest not appropriate. No ICC for test-retest calculated. Population only males.
	n.d.s.	Juhasz 1972 [99]	USA	E	Students^a^		12.0	NR	Internal consistency	*α*=0.95^a^			Inadequate	−	Insufficient information about participants and study procedures. Cronbach’s alpha for total score reported.
					Professors^b^		67.0			*α*=0.99^b^					
	n.d.s.	Evans et al. 1973 [100]	USA	E	Students	35	22.0	NR	Test-retest	Pearson corr. for total score *r*=0.91 Subscales: visual=0.67, auditory=0.74, tactile=0.82, kinaesthetic=0.74, gustatory=0.75, olfactory=0.72, organic=0.61.			Doubtful	-	Sample size and time interval for this analysis doubtful (6 weeks). Low test-retest reliability for organic and visual subscales.
	n.d.s.	Westcott & Rosenstock 1976 [101]	USA	E	Students	147	NR.	66♀, 81♂	Test-retest	Reliabilities ranged from 0.72 to 0.75			Doubtful	?	No information whether ICC or correlation for reliabilities were calculated.
									Internal consistency	*α* ranged from 0.91 to 0.94			Inadequate	?	Cronbach’s for total score reported. *Insufficient information reported for quality criteria rating.
	n.d.s.	White et al. 1977 [48]	AU	E	students	251	NR	89♀, 162♂	Test-retest	Total score=0.59 Subscales: visual=0.52, auditory=0.46, tactile=0.51, kinaesthetic=0.32, gustatory=0.46, olfactory=0.59, organic=0.51.			Inadequate	−	No information how reliability was calculated (Pearson or ICC). Time interval for test-retest was 12 months.
	n.d.s	Baranchok John 1995 [102]	MX + USA	S + E	Mexican students^a^	350	NR	159♀, 191♂	Internal consistency	**Both language versions** Total *α*=0.90^a^. Subscales: auditory=0.70, kinaesthetic=0.67, gustatory=0.76, olfactory=0.72, organic=0.70, cutaneous=0.63, visual=0.67 Total *α*=0.88^b^. Subscales: auditory=0.70, kinaesthetic=0.67, gustatory=0.73, olfactory=0.70, organic=0.67, cutaneous=0.62, visual=0.66			Very good	−	Translation process made with 30 students. High corr. *r*=0.98 between English and Spanish language version suggested semantic equivalence. Cronbach’s alpha for most scales >0.70.
					US students^b^	307		130♀, 177♂							
	n.d.s.	Sacco & Reda 1998 [103]	IT	I	Students	201	22.6	65♀, 136♂	Internal consistency	Total *α*=0.86. Subscales: auditory=0.65, kinaesthetic=0.58, gustatory=0.63, olfactory=0.64, organic=0.75, cutaneous=0.64, visual=0.67			Very good	−	Cronbach's alpha only for organic scale >0.70. *No information for structural validity reported.
	n.d.s.	Campos & Pérez-Fabello 2005 [104]	ES	S	Students	562	20.2	148♀, 414♂	Internal consistency	*α*=0.92			Inadequate	−	Cronbach’s for total score reported. Should be calculated for each subscales.
Clarity of Auditory Imagery Scale (CAIS)	n.d.s.	Willander & Baraldi 2010 [105]	SE	E/Se	Students	212	25.9	58♀, 154♂	Internal consistency	*α*=0.88			Very good	?	Cronbach’s alpha >0.70. Structural validity doubtful.
	n.d.s.	Campos 2011 [106]	ES	S	Students	234	19.6	47♀, 187♂	Internal consistency	*α*=0.82			Very good	?	Cronbach’s alpha >0.70. Structural validity indeterminate.
	Edu	Tuznik & Francuz 2019 [107]	PL	Po	Musicians	39	22.5	21♀, 18♂	Test-retest	N=87 ICC 0.85 (95% CI 0.76–0.91)			Adequate	+	Adequate sample size. ICC calculated and >0.70, formula described.
					Non- musicians	40	24.5	20♀, 20♂	Internal consistency	*α*=0.87			Very good	?	Cronbach’s alpha >0.70. Structural validity reported indeterminate.
Gordon Test of Visual imagery control (GTVIC)	n.d.s.	Juhasz 1972 [99]	USA	E	Students^a^	67	NR	NR	Internal consistency	*α*^a^=0.88			Doubtful	?	*Insufficient information about participants and study procedures. Cronbach’s alpha higher for smaller sample sizes.
					Professors^b^	12				*α*^b^=0.95					
	n.d.s.	Mckelvie & Gingras 1974 [108]	CA	E/F	Students	87	16.5	NR	Internal consistency	Split-half with the Spearmen-Brown formula 0.76			Inadequate	−	Cronbach’s alpha not calculated. No Information about test procedures.
						33	16.5	NR	Test-retest	Pearson corr. *r*=0.84			Doubtful	−	Unclear whether test conditions were similar. Sample size doubtful. ICC not calculated.
	n.d.s.	Westcott & Rosenstock 1976 [101]	USA	E	Students	147	NR	66♀, 81♂	Internal consistency	*α* ranged from 0.64 to 0.66			Very good	−	Very good sample size. Cronbach’s alpha <0.70.
									Test-retest	r ranged from 0.81 to 0.86			Doubtful	?	No information whether ICC or correlation for reliabilities calculated.
	n.d.s.	Hiscock 1978^2^ [109]	USA	E	Students	123	NR	55♀, 68♂	Internal consistency	Split-half, *r*=0.77			NA	NA	Authors reported several studies in one article. COSMIN + quality criteria rating could not be applied. Results only in discussion mentioned.
	n.d.s.	Hiscock 1978^3^ [109]	USA	E	Students	79	NR	36♀, 43♂	Internal consistency	Split-half, *r*=0.84			NA	NA	
	n.d.s.	Leboutillier & Marks 2002 [110]	UK	E	Students	167	20.0 (median)	52♀, 115♂	Study aim was to assess each item of the GTVIC for skewness through *z* distribution transformations. If provided scales were normal, analyses of construct validity and internal reliability were performed. All attempts to normalise the data failed and no further analysis was performed.				NA	NA	Study conclusion: measure should not be used as a continuous variable, because GTVIC was not designed as an interval scale.
	n.d.s.	Pérez-Fabello & Campos 2004 [111]	ES	S	Students	479	20.5	70♀, 409♂	Internal consistency	*α*=0.69			Very good	−	Cronbach’s alpha >0.70.
Imaging Ability Questionnaire (IAQ)	Med	Kwekkeboom 2000 [42]	USA	E	Participants from different sources	200	48.7	NR	Development	IAQ contained 54 items, two subscales: an absorption and an image subscale. Scoring 0–4. Item variance carried out with 200 participants. 4 items were eliminated. Item sensitivity tested with 80 (mean age 40.5) participants. 18 items were eliminated. 32 (21 absorption and 11 image) items remained in the final version.			Inadequate	NA	Patients were not asked regarding comprehensiveness and comprehensibility.
	Med	Kwekkeboom 2000 [42]	USA	E	Participants from different sources	200	48.7	NR	Internal consistency	54-item version *α*=0.95 32-item version Total *α*=0.93; absorption *α*=0.92; Image generation *α*=0.92.			Very good	+	Very good sample size. Cronbach’s alpha for each subscale calculated.
						84	53.0	NR	Test-retest	0.92			Doubtful	?	ICC not calculated. Insufficient information on how test-retest reliabilities was calculated.
Imagery Questionnaire by Lane	n.d.s.	Lane 1977 [112]	CA	E	Students	320	NR	122♀, 198♂	Internal consistency	Seven modalities: visual *α*=0.50 auditory *α*=0.53 cutaneous α=0.46 kinaesthetic *α*=0.57 gustatory *α*=0.56 olfactory *α*=0.64 feeling states *α*=0.53			Very good	−	Development process not described. No information about test procedures. Cronbach’s alpha >0.70.
Kids Imaging Ability Questionnaire (KIAQ)	Med	Kwekkeboom et al. 2000 [113]	USA	E	Children	58	9.9	19♀, 39♂	Internal consistency	**17-item KIAQ** 1^st^ Time, *N*=54 analysed: *α*=0.70 absorption scale, *α*=0.61 image generation scale, total *α*=0.76. 2^nd^ Time, *N*=44 analysed: *α*=0.69 absorption scale, *α*=0.58 image generation scale, total *α*=0.75.			Very good	−	Low sample size considered for 2^nd^Time (*n*<50). Cronbach’s alpha not for all items >0.70.
									Test-retest	*N*=44 analysed, Pearson’s corr. coefficient *r*=0.73			Doubtful	?	Sample size < 50. ICC not calculated. Corr. coefficient does not consider systematic error.
Mental Imagery Scale (MIS)	n.d.s	Dercole et al. 2010 [114]	IT	I	Participants characteristics NR	262	29.0	92♀, 170♂	Development	MIS: 33 items generated: image formation speed, permanence/stability, dimensions, level of details and grain, distance and depth of field/perspective. rating scale 1–5.			Inadequate	NA	Participants not clearly described. No information provided of the target population for which the assessment was developed.
	n.d.s	Dercole et al. 2010 [114]	IT	I	Participants characteristics NR	262	29.0	92♀, 170♂	Internal consistency	Inter-item analyses for components: Stability=0.77, Distance=0.76, Level of Details=0.74, Rapidity=0.72, Dimensions= 0.60, Perspective=0.69.			Very good	-	Cronbach’s alpha for two items >0.70.
Plymoth sensory imagery questionnaire (Psi-Q)	n.d.s.	Andrade et al. 2014^1^ [115]	UK	E	Students	NA	NR	NA	Development	7 modalities: vision, sound, smell, taste, touch, bodily sensation, emotional feeling, five items for each modality, total 35 items.			Inadequate	NA	Several studies in this article reported. No information on target population. Only evaluated with students.
						41	NR	NR	Test-retest	*r*=0.71(subscales ranged from 0.43 to 0.84)			Inadequate	−	Time interval between measurements not appropriate. Sample size doubtful.
						404	NR	NR	Internal consistency	*α*=0.96			Inadequate	−	Cronbach’s alpha for total score reported. Sex not reported.
	n.d.s.	Andrade et al. 2014^2^ [115]	UK	E	Students	209	NR	NR	Internal consistency	*α*=0.93			Inadequate	−	Cronbach’s alpha for total score reported. Sex not reported.
	n.d.s.	Andrade et al. 2014^3^ [115]	UK	E	Students	212	23.4 (median)	59♀, 153♂	Internal consistency	Long form *α*=0.96 Short form *α*=0.94			Inadequate	−	Cronbach’s alpha for total score reported.
	n.d.s.	Pérez-Fabello & Campos 2020 [116]	ES	S	Students	394	21.0	101♀, 293♂	Internal consistency	vision *α*=0.68 sound *α*=0.77 smell *α*=0.72 taste *α*=0.75 touch *α*=0.75 body *α*=0.68 emotions *α*=0.72			Very good	+	Very good sample size, Cronbach’s alpha for each subscales reported, structural validity evaluated and sufficient.
Sport Imagery Ability Measure (SIAM)	Sport	Watt 2003^1^ [36]	AU	E	Students and athletes	5	15-16	NR	Development	72. Items. Five imagery dimensions (vividness, control, ease, speed, duration) in any of six sensorial modalities: visual, auditory, kinaesthetic, olfactory, gustatory, and tactile. Scoring: each item out of 100.			Doubtful	NA	Several studies in this article reported. Sample size doubtful. Insufficient *Information about data recording (e.g. interviews recorded and transcribed verbatim) and data analysis.
	Sport	Watt 2003^1^ [36]	AU	E	Students and athletes	474	18.42	268♀, 206♂	Internal consistency	Gustatory *α*=0.80 Auditory *α*=0.68 Duration *α*=0.72 Vividness *α*=0.70 Speed *α*=0.65	Olfactory *α*=0.81 Tactile *α*=0.76 Emotion *α*=0.76 Control *α*=0.73 Visual *α*=0.68 Ease *α*=0.63		Very good	?	For quality criteria rating: 1/3 of all items are <0.70. A subgroup analysis regarding age or sport and physical activities experience may reveal more homogeneous data.
Revised Sport Imagery Ability Measure (SIAM-R)	Sport	Watt 2003^1^ [36]	AU	E	Students and athletes	47	NR	NR	Test-retest	Gustatory *r*=0.83 Auditory *r*=0.51 Kinaesthetic *r*=0.68 Duration *r*=0.57 Vividness *r*=0.59 Speed *r*=0.44	Olfactory *r*=0.78 Tactile *r*=0.70 Emotion *r*=0.63 Control *r*=0.61 Visual *r*=0.51 Ease *r*=0.44		Doubtful	?	Sample Size doubtful. ICC not calculated. Insufficient information on how test-retest reliabilities were calculated.
	Sport	Watt 2003^2^ [36]	AU	E	Athletes and students	633	18.77	334♀, 299♂	Internal consistency	Gustatory *α*=0.87 Auditory *α*=0.75 Kinaesthetic *α*=0.77 Control *α*=0.79 Vividness *α*=0.75 Ease *α*=0.67	Olfactory *α*=0.84 Tactile *α*=0.80 Emotion *α*=0.75 Duration *α*=0.77 Speed *α*=0.66 Visual *α*=0.76		Very good	?	Very good sample size. High internal consistency. However, last 3 items <0.70.
						58	NR.	NR	Test-retest	Gustatory *r*=0.76 Auditory *r*=0.41 Kinaesthetic *r*=0.58 Control *r*=0.66 Vividness *r*=0.56 Ease *r*=0.50	Olfactory *r*=0.65 Tactile *r*=0.61 Emotion *r*=0.75 Duration *r*=0.59 Speed *r*=0.53 Visual *r*=0.67		Doubtful	?	ICC not calculated. Insufficient information on how test-retest reliabilities were calculated.
Sport Imagery Ability Questionnaire (SAIQ)	Sport	Williams & Cumming 2011 [117]	UK	E	Athletes	403	20.2	198♀, 205♂	Development	35 items designed to asses five types of imagery content: CS= cognitive specific, CG= cognitive general, MS= motivational specific, MG-A= motivational general arousal, MG-M= motivational general mastery. After factor analysis 20-item version was used in further development.			Doubtful	NA	Data collection and analyses not clearly described, e.g. how they designed 35-item version. No group meetings or interviews mentioned.
	Sport	Williams & Cumming 2011^1^ [117]	UK	E	Athletes	375	24.7	179♀, 196♂	Internal consistency	**20-item version of SIAQ**			Very good	+	Authors reported results from 4 studies in this article. Criterion level for CR 0.70 and AVE 0.50.
											**CR**	**AVE**			
										Skill imagery:	0.74	0.50			
										Strategy imagery	0.75	0.50			
										Goal imagery	0.79	0.57			
										Affect imagery	0.78	0.55			
	Sport	Williams & Cumming 2011^2^ [117]	UK	E	Athletes	363	24.8	175♀, 188♂	Internal consistency	**12-item version of SIAQ** CR ranged from 0.76 to 0.80 AVE ranged from 0.52 to 0.58			Very good	+	Criterion level for CR 0.70 and AVE 0.50.
	Sport	Williams & Cumming 2011^3^ [117]	UK	E	Athletes	426	NR	199♀, 227♂	Internal consistency	**Modified SIAQ: 15-item version (3 new items added to 12-item version) + fifth subscale added: mastery** CR ranged from 0.76 to 0.86 AVE ranged from 0.51 to 0.68			Very good	+	Sample size very good. Criterion level for CR 0.70 and AVE 0.50.
						116	NR	NR	Test-retest	Skill ICC=0.83 Strategy ICC=0.86 Goal ICC=0.86 Affect ICC=0.75 Mastery ICC=0.85			Doubtful	+	Test-retest interval doubtful. Test conditions were presumably similar. All ICC values > 0.70.
	Sport	Williams & Cumming 2011^4^ [117]	UK	E	Athletes	220	19.5	86♀, 134♂	Internal consistency	**Modified SIAQ: 15 items, five subscales** CR ranged from 0.78 to 0.86 AVE ranged from 0.55 to 0.67			Very good	+	Sample size very good. Criterion level for CR 0.70 and AVE 0.50.
Survey of mental imagery	n.d.s.	Switras 1978 [118]	USA	E	Students	350	NR	129♀, 221♂	Internal consistency Form A	Controllability	Vividness		very good	?	For development 1200 participants involved but no characteristics reported. Two versions of the Survey of Mental Imagery assessments: Form A and B.
										Visual *α*=0.79	*α*=0.88				
										Auditory *α*=0.78	*α*=0.87				
										Gustatory *α*=0.86	*α*=0.90				
										Tactile *α*=0.78	*α*=0.85				
										Somesthetic *α*=0.68	*α*=0.78				
										Kinaesthetic *α*=0.81	*α*=0.89				
	n.d.s.	Switras 1978 [118]	USA	E	Students	350	NR	129♀, 221♂	Internal consistency Form B	Controllability	Vividness		Very good	?	# Students received course credits for participation. Cronbach’s alpha calculated including all subscales. Structural validity indeterminate.
										Visual *α*=0.83	*α*=0.89				
										Auditory *α*=0.78	*α*=0.87				
										Olfactory *α*=0.80	*α*=0.85				
										Gustatory *α*=0.88	*α*=0.91				
										Tactile *α*=0.76	*α*=0.84				
										Somesthetic *α*=0.71	*α*=0.79				
										Kinaesthetic *α*=0.80	*α*=0.87				
	n.d.s.	Grebot 2003 [119]	FR	F	Teachers	162	36.0	31♀, 131♂	Internal consistency	French version with 52 items: only visual, auditory, somesthetic and kinaesthetic modalities. Controllability: Visual *α*=0.66, Auditory *α*=0.88, Somesthetic *α*=0.77, Kinaesthetic *α*=0.91 Vividness: Visual *α*=0.86, Auditory *α*=0.91, Somesthetic *α*=0.83, Kinaesthetic *α*=0.93 Formation: Visual *α*=0.88, Auditory *α*=0.89, Somesthetic *α*=0.80, Kinaesthetic *α*=0.93			Very good	?	Only form A used. Cronbach’s alpha calculated for each subscale. Unclear development process on French and new dimension ‘formation’. *Insufficient information for quality criteria rating regarding structural validity.
Visual Elaboration Scale (VES)	n.d.s.	Slee 1976 [120]	AU	E	Students	40	NR	NR	Internal consistency	**Original form of VES (**Three absent objects and 15 items) Item-total correlation (range) 1. object *α*=0.25–0.48 2. object *α*=0.30–0.56 3. object *α*=0.23–0.51 Five items did not show sig. corr. with total score and were removed from original form.			doubtful	?	Only item-total corr. calculated and no Cronbach’s alpha or KR-20. Sample size doubtful. No information about participants.
					Students	50	NR	NR	Internal consistency	**Second form of the scale** (four objects and 20 items) Item-total correlation (range) 1. object *α*=0.35–0.56 2. object *α*=0.27–0.74 3. object *α*=0.34–0.62 4. object *α*=0.25–0.55 KR-20 reliability was 0.78 Five items were removed from second form and the 15 items remaining were accepted as a final form. KR-20 calculated for final form (*N*=50) 0.78.			Doubtful	?	Only a few information about participants. # Participants received course credits for their participation. *Insufficient information for quality criteria rating regarding structural validity.
Vividness of Olfactory Imagery Questionnaire (VOIQ)	n.d.s.	Gilbert et al. 1998 [121]	USA	E	Fragrance experts^a^	122	NR	63♀, 59♂	Internal consistency	Split-half reliability coefficient 0.77^a^/ 0.86^b^			Inadequate	−	Cronbach’s alpha not calculated. Structural validity not mentioned.
					Non-expert controls^b^	95		50♀, 45♂							
Vividness of Object and Spatial Imagery Questionnaire (VOSI)	n.d.s.	Blazhenkova Olesya 2016^1^ [122]	TU	NR	Students	111	21.8	53♀, 58♂	Development	Pilot version: 9 items for object imagery vividness and 9 items for spatial imagery vividness. Rating scale 1–5. Factor analysis confirmed two factors: object and spatial imagery. Sign. and positive corr. found between VOSI pilot and OSIQ.			inadequate	NA	Results of two studies in this article reported.
	n.d.s.	Blazhenkova Olesya 2016^2^ [122]	TU	NR	Students	205	21.0	95♀, 110♂	Development	The final version of VOSI: 14 items assessing object imagery vividness and 14 items assessing spatial imagery.			Inadequate	NA	For both versions (pilot and final), no information provided on how data were collected for item creating. Target population not mentioned. Only students participated and were reimbursed with course credits or chocolate bars.
									Internal consistency	Object vividness scale: *α*=0.88 Spatial vividness scale: *α*=0.85			Inadequate	-	Cronbach’s alpha for total score reported.
Vividness of Visual Imagery Questionnaire (VVIQ)	n.d.s.	Marks 1973 [26]	NZ	E	Students	68	NR	NR	Test-retest	*r*=0.74			Doubtful	?	Test-retest reliability only briefly mentioned. No information on how test-retest was calculated.
	n.d.s.	Mckelvie & Gingras 1974 [108]	CA	E	Students	87	16.5	NR	Internal consistency	Split-half with the Spearmen-Brown formula 0.93			Inadequate	−	Cronbach’s alpha not calculated. No information about test procedures.
	n.d.s.	Mckelvie 1974 [108]	CA	E	Students	33	16.5	NR	Test-retest	Pearson corr. *r*=0.67			Doubtful	−	Unclear if the test-retest conditions were similar. Sample size doubtful.
	n.d.s.	Rossi 1977 [123]	USA	E	Students	119	NR	NR	Test-retest	0.73			Doubtful	?	Time interval doubtful. Participants characteristics not described. No information on how test-retest was calculated.
									Internal consistency	*α*=0.91			Doubtful	?	No information about participants characteristics and test procedures. Structural validity evaluated but indeterminate.
	Sport	Isaac et al. 1986 [27]	NZ	E	Students/ athletes	220	NR	NR	Test-retest	Pearson’s corr. coefficient *r*=0.75			Doubtful	−	ICC no calculated. *Insufficient information for quality criteria rating.
	Sport	Eton et al. 1998 [86]	USA	E	Recreational athletes + non-athletes	36	NR	NR	Test-retest	Pearson’s corr. coefficient for eyes open *r*=0.48, eyes closed *r*=0.62			Doubtful	−	Small sample size. ICC not calculated. *Insufficient information for quality criteria rating.
					Varsity athletes	51	NR	27♀, 24♂	Internal consistency	Eyes open *α*=0.91 Eyes closed *α*=0.93			Very good	?	*Insufficient information for quality criteria rating regarding structural validity.
					Recreational athletes	48		24♀, 24♂							
					Non-athletes	26		14♀, 12♂							
Vividness of Visual Imagery Questionnaire (VVIQ)	n.d.s.	Campos et al. 2002 [124]	ES	S	Secondary school students	850	13.3	428♀, 422♂	Internal consistency	*α*=0.88			Very good	?	High internal consistency but not reported whether for eyes open or closed version. Structural validity indeterminate.
	n.d.s.	Leboutillier & Marks 2001 [125]	UK	E	Students	198	23.86	75♀, 123♂	Internal consistency	Nature scenes overall *α*=0.88 (range 0.31–0.67) Person scene overall *α*=0.80 (range 0.42–0.62) Ship scene overall *α*=0.76 (range 0.36–0.52)			Very good	+	Only the eyes-open version of VVIQ was evaluated in this study.
	n.d.s.	Campos & Pérez-Fabello, 2009 [126]	ES	S	Students	279	20.1	117♀, 162♂	Internal consistency	*α*=0.91			Very good	?	*Insufficient information for quality criteria rating regarding structural validity.
Revised version Vividness of Visual Imagery Questionnaire (VVIQ-2)	n.d.s.	Campos & Pérez-Fabello, 2009 [126]	ES	S	Students	279	20.1	117♀, 162♂	Internal consistency	*α*=0.94			Very good	?	*Insufficient information for quality criteria rating.
	n.d.s.	Campos 2011 [106]	ES	S	Students	206	19.7	43♀, 163♂	Internal consistency	*α*=0.91			Very good	?	# Students received course credits for participation. *Insufficient information for quality criteria rating regarding structural validity.
Vividness of Visual Imagery Questionnaire- Revised version (VVIQ-RV)	n.d.s.	Campos 2011 [106]	ES	S	Students	206	19.7	43♀, 163♂	Internal consistency	*α*=0.96			Very good	?	#, *Insufficient information for quality criteria rating.
Vividness of Visual Imagery Questionnaire –Modified (VVIQ-M)	n.d.s.	Halpern 2015 [97]	USA	E	Volunteers	76	22.6	22♀, 54♂	Internal consistency	*α*=0.91			Very good	?	*Insufficient information for quality criteria rating.
Vividness of Wine Imagery Questionnaire (VWIQ)	Edu	Croijmans et al. 2019 [127]	NL	E	Volunteers with experience with wine	50	NR	NR	Test-retest	Smell *r*=0.87 Taste *r*=0.83 Vision *r*=0.79			Doubtful	?	Only corr. calculated. ICC not calculated. Sample size doubtful and no description of participants.
						83	40.8	71♀,12♂	Internal consistency	Omega coefficient Smell 0.95 Taste 0.96 Vision 0.88			Very good	?	Omega could be acceptable but structural validity may be insufficient. This should be evaluated with a larger sample size.
**b. Assessments of mental rotation**															
Card Rotation Test	n.d.s.	Ekstrom et al. 1976 [128]	USA	E	NR	NR	NR	NR	NR	NR			NA	NA	Ekstrom et at. 1976 published ‘Manual for Kit of Factor-Referenced Cognitive Tests’. First description of Card Rotation Test and Cube Comparison Test.
Cube Comparison Test	n.d.s.	Ekstrom et al. 1976 [128]	USA	E	NR	NR	NR	NR	NR	NR			NA	NA	
German Test of the Controllability of Motor Imagery in older adults (TKBV)	n.d.s.	Schott 2013 [29]	DE	G	Healthy	195	57.3	102♀, 93♂	Internal consistency	Two scales (Recognition and Free recall) with total 20 items, 10 items per scale. *α*=0.89 for Free recall *α*=0.73 for Recognition			Very good	+	Very good sample size. Cronbach’s alpha calculated for each scale. Structural validity evaluated.
Hand Laterality Task	n.d.s.	Hirschfeld et al. 2013 [30]	DE	G	Students	99	21.2	20♀, 79♂	Internal consistency	**Split-Half with the Spearman-Brown** Intercepts: blocked group=0.79 and mixed group=0.82. Slopes: blocked group=0.79 and mixed=0.20.			Inadequate	−	Cronbach’s alpha not calculated. Unacceptable low reliability for the slopes mixed group.
									Test-retest	Corr. Intercepts: blocked group *r*=0.68 and mixed group *r*=0.51 Slopes: blocked group *r*=0.69 and mixed *r*=0.55.			Doubtful	?	Time interval (6 weeks) for test-retest doubtful. ICC not calculated. Corr. coefficient does not consider systematic error.
Left/Right Judgements (LRJ)	Med	Bray & Mosley 2011 [129]	AU	E	Patients with back pain^a^	5	46.0	1♀, 4♂	Test-retest	**Response time trunk rotation** ICC=0.87^a^/ ICC=0.74^b^ **Response time hands** ICC=0.70^a^/ ICC=0.95^b^ **Accuracy trunk rotation** ICC=0.92^a^/ ICC=0.80^b^ **Accuracy hands** ICC=0.92^a^/ ICC=0.87^b^			inadequate	+	ICC for accuracy and response time for all pictures (with trunk rotation and hands) was >0.70. However, very low sample size. Further studies with a large sample size needed.
					Healthy^b^	5	40.0	2♀, 3♂							
	n.d.s.	Zimney et al. 2018 [130]	USA	E	Students	50	24.3	15♀, 35♂	Test-retest	**Card-based LRJ** Accuracy: left ICC=0.60 (CI, 0.29–0.78), right ICC=0.79 (CI, 0.63–0.88) Response time: ICC=0.84 (CI, 0.06–0.95). **Tablet-based LRJ** Accuracy: left ICC=0.60 (CI, 0.31–0.77), right ICC=0.38 (CI, 0.04–0.64) Response time: ICC=0.90 (CI, 0.82–0.94)			Doubtful	?	Sample size and time interval for test-retest doubtful. ICC only for reaction time >0.70. ICC for accuracy very low.
									Measurement error	**Card-based LRJ** Accuracy: left SEM=2.55%, MDC=7.07%, right SEM=2.12%, MDC=5.86% Response time: SEM=0.16%, MDC=0.44% **Tablet-based LRJ** Accuracy: left SEM=4.89%, MDC=13.54%, right SEM=6.81%, MDC=18.87% Response time SEM=0.13%, MDC=0.37%			Doubtful	?	Sample size and time interval for test-retest doubtful. Minimal important change (MIC) not defined.
	n.d.s.	Williams et al. 2019^1^ [131]	AU	E	Healthy	20	55.3	5♀, 15♂	Test-retest	**Tablet version of LRJ** Accuracy ICC=0.82 Response time ICC=0.90			Doubtful	+	Results of two studies in this article reported. Only one day between test-retest. Sample size doubtful.
Judgement Test of Foot and Trunk Laterality	Med	Linder et al. 2016 [132]	SE	Se	LBP patients^a^	30	44.9	10♀, 20♂	Test-retest	Reliability between Test 1 and 2, ^a^*N*=24, ^b^*N*=26 ^a^ICC=0.51–0.75 ^b^ICC=0.59–0.85 Reliability between Test 2 and 3, ^a^*N*=21, ^b^*N*=23 ^a^ICC=0.63–0.91 ^b^ICC=0.51–0.89			Inadequate	?	Time interval between tests inappropriate. Doubtful sample size (<50). ICC by patients lower and <0.70, but not for all tasks.
					Healthy^b^	30	43.3	10♀, 20♂							
Map Rotation Ability Test (MRAT)	n.d.s.	Campos & Campos-Juanatey 2020 [133]	ES	S	Students	257	19.7	86♀, 171♂	Internal consistency	*α*=0.77			Very good	?	*Insufficient information for quality criteria rating regarding structural validity.
Mental Paper Folding	Psy	Shepard & Feng 1972 [134]	USA	E	Students	20	NR	11♀, 9♂	NR	NR			NA	NA	First description of measure of visuospatial ability, no psychometric properties evaluated.
Mental Rotation of Three-Dimensional Objects (MRT)	Psy	Shepard & Metzler 1971 [135]	USA	E	Healthy	8	NR	NR	NR	NR			NA	NA	First description of the mental rotation tasks, no psychometric properties evaluated.
	n.d.s.	Vandenberg & Kuse 1978 [136]	USA	E	Healthy	3268	NR	NR	Internal consistency	Kuder-Richardson 20 formula=0.88			NA	NA	Vandenberg & Kuse 1978 [136] reported finding from previous studies (partly unpublished data). Insufficient data reported for COSMIN and quality criteria evaluating.
					Students	312	NR	197♀, 115♂	Internal consistency	Split-Half with the Spearman-Brown formula 0.79			NA	NA	
					NR	336	NR	NR	Test-retest	Corr. =0.83			NA	NA	
					NR	456	NR	NR	Test-retest	Corr. =0.70			NA	NA	
	n.d.s.	Campos & Campos-Juanatey 2020 [137]	ES	S	Students	281	19.8	97♀, 184♂	Internal consistency	*α*=0.82			very good	?	*Insufficient information for quality criteria rating regarding structural validity.
Measure of the Ability to Form Spatial Mental Imagery (MASMI)	n.d.s.	Campos 2009 [96]	ES	S	Students	138	20.1	63♀, 75♂	Internal consistency	*α*=0.93			Very good	?	*Insufficient information for quality criteria rating regarding structural validity.
	n.d.s.	Campos 2013 [138]	ES	S	Students	254	19.5	108♀, 146♂	Internal consistency	*α*=0.93			Very good	?	*Insufficient information for quality criteria rating regarding structural validity.
	n.d.s.	Campos & Campos-Juanatey 2020 [137]	ES	S	Students	281	19.8	97♀, 184♂	Internal consistency	*α*=0.84			Very good	?	*Insufficient information for quality criteria rating regarding structural validity.
Measure of the Ability to Rotate Mental Images (MARMI)	n.d.s.	Campos 2012 [139]	ES	S	Students	354	19.5	45♀, 309♂	Internal consistency	*α*=0.90			Very good	?	Very good sample size but more than 90% females. No information about structural validity.
Shoulder specific left right judgement task (LRJT)	Med	Breckenridge et al. 2017 [140]	AU	E	Patients with shoulder pain	1413	42.9	NR	Internal consistency	*α*=0.95 for all 40 items (20 left and 20 right)			Very good	?	Very good sample size. A positive corr. reported for age and response time, but negative corr. for age and accuracy and between gender and response time. Structural validity not evaluated.
Spatial Orientation Skills Test (SOST)	n.d.s.	Campos & Campos-Juanatey 2020 [137]	ES	S	Students	281	19.8	97♀, 184♂	Internal consistency	*α*=0.83			Very good	?	*Insufficient information for quality criteria rating regarding structural validity.
**c. Assessments of mental imagery to distinguish between different types of imagers**															
Object-Spatial Imagery Questionnaire (OSIQ)	n.d.s.	Blajenkova et al. 2006^1^ [34]	USA	E	Students	214	20.33	108♀, 106♂	Development	After PCA 30 items (15 spatial and 15 object imagery) were retained. Two subscales: object and spatial imagery. Scoring 0–4.			Inadequate	NA	Results of four studies reported. There is no clear description of the target population for which the OSIQ was developed. Only with psychology students evaluated.
									Internal consistency	Object scale *α*=0.83 Spatial scale *α*=0.79			Very good	+	Test-retest after 1 week.
					Students	24	22.9	4♀,20♂	Test-retest	Object *r*=0.81 Spatial *r*=0.95			Doubtful	?	Corr. calculated and no ICC calculated.
Object-Spatial Imagery and Verbal Questionnaire (OSVIQ)	n.d.s.	Blazhenkova & Kozhevnikov 2009^1^ [35]	USA	E	Students	38	NR	NR	Development	45 Items: 15 object, 15 spatial, 15 verbal. 5-point scale.			Inadequate	NA	Results of four studies reported. # There is not clear description provided of the target population for which the OSVIQ was developed. Only with psychology students evaluated.
					Students and professionals from different fields	625	24.0	251♀,374♂	Internal consistency	Verbal scale *α*=0.74 Object scale *α*=0.83 Spatial scale *α*=0.79			Very good	?	Cronbach's alpha >0.70. Structural validity indeterminate.
	n.d.s.	Blazhenkova & Kozhevnikov 2009^2^ [35]	USA	E	Students	41	NR	NR	Test-retest	Corr. calculated: Verbal *r*=0.73 Object *r*=0.75 Spatial *r*=0.84			Doubtful	?	Sample size < 50. Corr. calculated and no ICC calculated
	n.d.s.	Campos 2011 [106]	ES	S	Students	213	19.6	62♀,151♂	Internal consistency	Object scale *α*=0.77 Spatial scale *α*=0.81 Verbal scale *α*=0.72			Very good	?	Cronbach’s alpha >0.70. Structural validity indeterminate.
	n.d.s.	Campos & Campos-Juanatey 2020 [137]	ES	S	Students	281	19.8	97♀, 184♂	Internal consistency	Verbal scale *α*=0.72 Object scale *α*=0.79 Spatial scale *α*=0.81			Very good	?	*Insufficient information for quality criteria rating regarding structural validity.
Paivio’s Individual Differences Questionnaire (IDQ, 86 items)	n.d.s.	Paivio & Harshman 1983 [141]	CA	E	NR	NR	NR	NR	Development	IDQ assess verbal and imaginal habits, preferences and abilities. Total 86 items with possible answer 'true' or ‘falsh’ to each item.			Inadequate	NA	Insufficient information reported about qualitative data collection for questionnaire construction. Target population unclear.
					Students	713	NR	NR	Internal consistency	Verbal scale 47 items *α*=0.86 Imagery scale 39 items *α*=0.82			Very good	+	Very good sample size. No information on sex and age. Cronbach’s alpha >0.70.
Paivio’s Individual Differences Questionnaire (shorted IDQ, 34 items)	n.d.s.	Kardash et al. 1986 [142]	USA	E	Students	189	NR	99♀, 90♂	Internal consistency	Verbal scale 27 items *α*=0.71 Imagery scale 7 items *α*=0.52			Very good	-	Short version revealed lower internal consistency. Cronbach’s alpha <0.70.
Revised Paivio’s Individual Differences Questionnaire (IDQ, 72 items)	n.d.s.	Hiscock 1978^1^ [109]	USA	E	Students	48^1^	NR	48♂	Internal consistency	Imagery scale *α*=0.80^1^; *α*=0.81^2^; *α*=0.87^3^ Verbal scale *α*=0.83^1^; *α*=0.86^2^; *α*=0.88^3^			Very good	+	3 student groups. Sample size in first group (*N*=48) doubtful. Cronbach’s alpha consistent in all three groups >0.70.
						114^2^		57♀, 57♂							
						79^3^									
								36♀, 43♂							
	n.d.s.	Hiscock 1978^1^ [109]	USA	E	Students	58	NR	NR	Test-retest	Imagery scale 0.84 Verbal scale 0.88.			Doubtful	?	4 studies reported in this article. Insufficient information on how test-retest reliabilities were calculated.
Sussex Cognitive Styles Questionnaire (SCSQ)	n.d.s.	Mealor et al. 2016^1^ [143]	UK	E	NA	NA	NA	NA	Development	Total 84 items generated: 22 from OSIVQ, 4 from IDQ, 24 from Systemising Quotient questionnaire, 7 from the ‘Attention to Detail’ subscale of the Autism Quotient. 27 items generated by authors.			Inadequate	NA	Target population and context of use unclear. Item generation only based on existing questionnaire, without asking of experts or target population.
					Students	1542	27.0	586♀, 956♂	Internal consistency	Imagery ability *α*=0.88 Technical /Spatial *α*=0.89 Language and Word Forms *α*=0.80 Need for Organisation *α*=0.77 Global bias *α*=0.74 Systemising Tendency *α*=0.73			Very good	?	Sample size good. Cronbach’s alpha calculated for each scale and >0.70. Structural validity indeterminate.
Verbalizer-Visualiser Questionnaire (VVQ)	n.d.s.	Stevens et al. 1986 [144]	USA	E	Students	184	NR	49♀, 123♂	Test-retest	Pearson corr. *r*=0.47			Doubtful	?	ICC not calculated. Insufficient information on how test-retest reliabilities were calculated.
	n.d.s.	Campos et al. 2004 [145]	ES	S	Students	969	14.2	496♀, 473♂	Internal consistency	*α*=0.30			Very good	-	Very good sample size for this analysis. Low internal consistency, Cronbach’s alpha >0.70.
	n.d.s.	Wedell et al. 2014 [146]	DE	G	Volunteers	476	24.1	99♀, 377♂	Internal consistency	*α*=0.04			Inadequate	-	Total Cronbach’s alpha calculated, but not for each scale. Very low internal consistency, Cronbach’s alpha >0.70.
**d. Assessments of use of mental imagery**															
Children’s Active Play Imagery Questionnaire (CAPIQ)	Sport	Cooke et al. 2014^1^ [147]	CA	E	None	NA	NA	NA	Development	Based on existing literature 16 items were generated. 5-point scale.			Doubtful	NA	2014^1^=phase 1. Item generation based only on existing literature. Target population was not involved in item generation.
	Sport	Cooke et al. 2014^2^ [147]	CA	E	Children	302	10.0	145♀, 157♂	Internal consistency	Capability *α*=0.82 Social *α*=0.71 Fun *α*=0.65			Very good	−	Cronbach’s alpha for scale ‘fun’ <0.70.
	Sport	Cooke et al. 2014^3^ [147]	CA	E	Children	252	10.4	118♀, 134♂	Internal consistency	Capability *α*=0.82 Social *α*=0.73 Fun *α*=0.82			Very good	?	Cronbach’s alpha for each scale calculated. Structural validity evaluated but insufficient.
	Sport	Kashani et al. 2017 [148]	IR	Pe	Students	60	NR	NR	Test-retest	Capability ICC=0.87 Social ICC=0.88 Fun ICC=0.87			Adequate	+	Adequate sample size, ICC >0.70.
Exercise Imagery Questionnaire-Aerobic Version EIQ-AV	Sport	Hausenblas et al. 1999^2^ [149]	CA	E	Students exercisers^a^	307	22.9	9♀,296♂	Development	EIQ-AV evaluated use of exercise imagery with 23 items. Three scales: Appearance, Energy, and Technique. Scoring: 9-point scale.			doubtful	NA	Results from 3 studies reported in this article. Data collection with another sample of 144 (Phase 1) athletes provided basis for item development. However, insufficient data reported how data were analysed and if participants were asked about comprehensibility and comprehensiveness.
					Students exercisers^b^	171	22.4	3♀,168♂							
	Sport	Hausenblas et al. 1999^3^ [149]	CA	E	Students exercisers^a^	307	22.9	9♀,296♂	Internal consistency	Cronbach’s alpha calculated for three factors for both samples ranged from 0.81 to 0.90.			Doubtful	?	Unclear whether Cronbach’s alpha for each factor separately calculated for the two samples.
					Students exercisers^b^	171	22.4	3♀,168♂							
					Students exercisers^a^	144	22.0	16♀,128♂	Internal consistency	Calculated Cronbach’s alphas for the 3 factors for both samples ranged from 0.71 to 0.85 , with one exception; the alpha value for Technique for sample 1 was 0.65.			Doubtful	?	Cronbach’s alpha presumably calculated for each scale, but only range was reported. Cronbach’s alpha for 1 scale >0.70.
					Students exercisers^b^	267	22.4	5♀,262♂							
					Students exercisers	18	21.6	NR	Test-retest	Five days apart, *r*=0.88			Doubtful	?	Small sample size. Test procedure not described. ICC not calculated.
Exercise Imagery Questionnaire-Aerobic Version EIQ-AV	Sport	Pérez-Fabello & Campos 2020 [150]	ES	S	Students	166	20.1	127♀,39♂	Internal consistency	**Three factors** Appearance *α*=0.78, CR=0.59 Energy *α*=0.75, CR=0.34 Technique *α*=0.78, CR=0.64 **Two factors** Energy CR=0.30 Technique CR=0.41 Cronbach’s alpha total >0.70			Very good	?	Sample size good, Cronbach’s alpha for each subscale reported and was >0.70 but CR below recommended values.
Sport Imagery Questionnaire (SIQ)	Sport	Hall et al. 1998^1^ [151]	CA	E	Athletes	113	23.6	53♀,60♂	Development	46 items designed to asses 4 types of imagery content: CS= cognitive specific, CG= cognitive general, MS= motivational specific, MG= motivational general. After factor analysis, MG factor was found to represent two distinct subscales: MG-A= motivational general arousal and MG-M= motivational general mastery.			doubtful	NA	Data from 3 different studies in the article included. Insufficient data reported about qualitative data collection to identify relevant items.
									Internal consistency	Motivational specific (MS) *α*=0.82 motivational general (MG) *α*=0.76 cognitive specific (CS) *α*=0.87 cognitive general (CG) *α*=0.77			Very good	+	Cronbach’s alpha for each scales >0.70.
	Sport	Hall et al. 1998^2^ [151]	CA	E	Athletes	271	NR	184♀,87♂	Internal consistency	**30-item version** motivational specific (MS) *α*=0.88, motivational general arousal (MG-A) *α*=0.70 motivational general mastery (MG-M) *α*=0.83 cognitive specific (CS) *α*=0.85 cognitive general (CG) *α*=0.75			Very good	+	Cronbach’s alpha for each scales >0.70.
	Sport	Vurgun et al. 2012 [152]	TR	Tu	Athletes	142	21.8	100♀,42♂	Test-retest	Motivational specific 0.76 Motivational general arousal 0.60 Cognitive specific 0.72 Cognitive general 0.62 Motivational general mastery 0.71			Adequate	?	ICC presumably calculated but without sufficient information on the procedure (model and formula not described). Reliability coefficient for 2 subscales <0.70.
									Internal consistency	Motivational specific *α*=0.91 Motivational general arousal *α*=0.83 Cognitive specific *α*=0.88 Cognitive general *α*=0.88 Motivational general mastery *α*=0.85			Very good	+	Cronbach’s alpha for each subscales >0.70. Structural validity reported and results are close to the results from the original study. However, low sample size for validity evaluation.
	Sport	Ruiz & Watt 2014 [153]	Not clear	S	athletes	361	24.1	234♀,29♂	Internal consistency	**30-item version** Cognitive specific (CS) *α*=0.81 Cognitive general (CG) *α*=0.72 Motivational specific (MS) *α*=0.86 Motivational general arousal (MG-A) *α*=0.73 Motivational general mastery (MG-M) *α*=0.83			very good	+	Cronbach’s alpha for each scales >0.70.
Sport Imagery Questionnaire for Children (SIQ-C)	Sport	Hall et al. 2009^1^ [154]	CA	E	Young athletes	428	10.9	137♀,291♂	Internal consistency	Cognitive specific (CS) *α*=0.80 Cognitive general (CG) *α*=0.69 Motivational specific (MS) *α*=0.75 Motivational general arousal (MG-A) *α*=0.69 Motivational general mastery (MG-M) *α*=0.82			Very good	+	Several studies reported. Development could not be evaluated (insufficient data reported). Finally, 21-item version of SIQ-C was evaluated. 2 scales with *α*=0.69 may be viewed as sufficient.
	Sport	Hall et al. 2009^2^ [154]	CA	E	Young athletes	628	NR	283♀,345♂	Internal consistency	Cognitive specific (CS) *α*=0.77 Cognitive general (CG) *α*=0.62 Motivational specific (MS) *α*=0.70 Motivational general arousal (MG-A) *α*=0.77 Motivational general mastery (MG-M) *α*=0.70			Very good	?	Calculated Cronbach’s alpha was lower by higher sample size. CG scale <0.70.
Spontaneous Use of Imagery Scale (SUIS)	n.d.s.	Reisberg et al. 2003 [155]	USA	E	Researcher in imagery field	150	39.4	NR	Internal consistency	Inter-item corr. was for all items 0.98 or higher.			Doubtful	?	Only inter-item corr. calculated, no Cronbach’s alpha. *No information regarding structural validity.
	n.d.s.	Nelis et al. 2014 [156]	UK	E	Students^a^	491	18.6	88♀,403♂	Internal consistency	*α*^a^=0.76 *α*^b^=0.72 *α*^c^=0.72			Very good	+	# Students received course credits for participation. Very good sample size. Structural validity reported. Cronbach’s alpha >0.70.
					Volunteers^b^	373	34.9	119♀,254♂							
					Students^c^	433	18.4	82♀,351♂							
					Students	49	NR	NR	Test-retest	ICC=0.69			Inadequate	+	Time interval of 5 months not appropriate. Sample size doubtful. ICC almost 0.70.
	n.d.s.	Görgen et al. 2016^1^ [157]	DE	G	Students	216	23.7	60♀,156♂	Internal consistency	*α*=0.66			Very good	−	Results from 2 studies reported in this article. 2015^1^=study 1. Cronbach’s alpha <0.70.
	n.d.s.	Görgen et al. 2016^2^ [157]	DE	G	Students	447	24.9	161♀,286♂	Internal consistency	**SUIS 17-item version** *α*=0.85			Very good	+	2015^2^=study 2. Very good sample size. Cronbach’s alpha >0.70.
	n.d.s.	Tanaka et al. 2018^1^ [158]	JP	J	Students	126	20.6	66♀,60♂	Test-retest	Pearson corr. *r*=0.76			Adequate	?	Results from two studies reported in this article. 2018^1^=study 1. ICC not calculated.
									Internal consistency	*α*=0.66			Very good	−	Cronbach’s alpha <0.70.

Legend: The superscript numbers were used to distinguish the results per group

Disciplines in which field the tool was evaluated: Edu Education, Med Medicine, Psy Psychology, n.d.s. not discipline-specific healthy participants/students

Country abbreviations: *AU* Australia, *CA* Canada, *CO* Columbia, *DE* Germany, *ES* Spain, *FR* France, *IR* Iran, *IS* Island, *IT* Italy, *JP* Japan, *MX* Mexico, *NL* Netherlands, *NZ* New Zealand, *PL* Poland, *SE* Sweden, *TR* Turkey, *UK* United Kingdom, *USA* United States of America

Language of the tool: *E* English, *F* French, *G* German, *I* Italian, *S* Spanish, *Se* Swedish, *J* Japanese, *Po* Polish, *Pe* Persian

*α* Cronbach’s alpha, *AVE* average variance extracted, *CI* confidence interval, *corr. *correlation, *CR* composite reliability, *COSMIN* Consensus-based Standards for the selection of health Measurement Instruments Risk of Bias Checklist, *ICC* interclass correlation coefficient, *KR-20* Kuder–Richardson, *LBP* low back pain, *MDC* minimal detectable change, *N* Sample size, *NA* Not applicable, *NR* Not reported, *PCA* principal component analysis, *SEM* standard error of measurement, *sign.* significant, *TKBV* Test zur Kontrollbarkeit der Bewegungsvorstellungsfähigkeit

Quality Criteria=see Table 1 and Legend for explanation of quality criteria

# methods could be doubtful, students received a course credits for participation. It could be interpreted that there was a certain dependency/necessity to participate, but it was not taken into account by the COSMIN evaluation

Quality Criteria: ‘+’ = sufficient, ‘−’ = insufficient, ‘?’ = indeterminate. *See Table 1 and Legend for explanation of quality criteria

**Table 6:** Mental imagery assessments: The characteristics of the included studies - Validity

Tool	Disciplines	Study	Country	Language	Study population				Validity		COSMIN	Quality Criteria	Comments
					Participants	***N***	Age mean (years)	Sex	Design	Results			
**a. General mental imagery in any sensorial modality**													
Auditory Imagery Scale (AIS)	n.d.s.	Gissurarson 1992 [94]	IS	E	Volunteers	160	33.0	70♀, 90♂	Construct validity- structural validity	PCA conducted. All seven items loaded on a single dimension. Item loaded 0.50–0.77.	Adequate	?	Only EFA conducted. *Not all information reported for quality criteria rating. CFA should be the next step.
									Construct validity- hypothesis testing	**Corr. AIS with VVIQ** *r*=0.48 **Corr. AIS with GTVIC** *r*=−0.23 **Know-group validity** Sex difference on the AIS were not significant.	Inadequate	?	Psychometric properties of comparator instrument not reported. Participant's characteristics not reported. Low corr. indicated, that there are two unrelated modalities: visual and auditory. But no corr. calculated with instrument which measures the same construct.
	n.d.s.	Allbutt et al. 2008 [159]	UK	E	Students	113	25.2	31♀, 82♂	Construct validity- hypothesis testing	**Corr. AIS with VVIQ-2** *r*=−0.35	Doubtful	?	Psychometric properties of comparator instrument insufficiently reported. Very low negative corr. between assessments. See comment above.
	n.d.s.	Campos 2017 [95]	ES	S	Students	444	20.4	190♀, 254♂	Construct validity- structural validity	CFA performed using on factor model: *χ*^2^//df=2.05, CFI=0.91, GFI=0.98, NNFI=0.80, RMSEA=0.05 and SRMR=0.04.	Doubtful	+	CFA performed but rotation method used was not described. Accepted model fit: CFI >0.95, or SRMR <0.08, or RMSEA <0.06.
									Construct validity- hypothesis testing	**Corr. ASI with CAIS** *r*=-0.49 **Corr. ASI with Bett's QMI** *r*=0.37	Doubtful	?	Psychometric properties of comparator instrument insufficiently reported. Not all results in accordance with the hypotheses. Corr. with comparator instrument <0.50.
Auditory Imagery Questionnaire (AIQ)	n.d.s.	Hishitani 2009^1^ [160]	JP	E	Students	193	20.3	146♀, 47♂	Construct validity- structural validity	PCA with oblimin rotation conducted. 3 factors extracted: relaxing sound, human voice, unpleasant sound. Factor loaded 0.31-0.74. Corr. factors 1 and 2 were 0.47, factors 2 and 3 were 0.47, factors 1 and 3 were 0.66. CFA performed using two-factor model (factor 1=human voice; factor 2=relaxing and unpleasant sound: GFI=0.92, CFI=0.93, RMSEA=0.07. CFA performed using hierarchical model composed of four factors: relaxing sound, human voice, mind's ear, unpleasant sound. GFI=0.94, CFI=0.96, RMSEA=0.06.	Very good	+	Steps of FA well described. Very good sample size. CFA with hierarchical model showed acceptable fit to the data. Accepted model fit: CFI >0.95, or SRMR <0.08, or RMSEA <0.06.
Auditory Imagery Questionnaire (AIQ)	n.d.s.	Hishitani 2009^2^ [160]	JP	E	Students	131	19.9	107♀, 24♂	Construct validity- hypothesis testing	**Corr. AIQ with VVIQ** *r*=0.48 **Know-group validity** Two subgroups were formed depending on whether the participants practiced music or not. Sig. differences between groups was found *p*<0.05.	Inadequate Doubtful	?	Psychometric properties of comparator instrument not reported. No corr. with comparator instrument which measures the same construct. Participant's characteristics not described.
	n.d.s.	Campos 2017 [95]	ES	S	Students	444	20.4	190♀, 254♂	Construct validity- structural validity	CFA performed using two-factor model: *χ*^2^/df=3.83, CFI=0.84, GFI=0.92, NNFI=0.86, RMSEA=0.08 and SRMR=0.07.	Doubtful	+	CFA performed but rotation method used not described. Accepted model fit: CFI >0.95, or SRMR <0.08, or RMSEA <0.06.
	n.d.s.	Campos 2017 [95]	ES	S	Students	444	20.4	190♀, 254♂	Construct validity- hypothesis testing	**Corr. AIQ with AIS** *r*=0.44 **Corr. AIQ with CAIS** *r*=−0.48 **Corr. ASI with Bett's QMI** *r*=0.59	Doubtful	?	Psychometric properties of comparator instrument insufficient reported. Results are not in accordance with the hypotheses. Stronger corr. between AIS and CAIS expected.
Bucknell Auditory Imagery Scale (BAIS)	n.d.s.	Halpern 2015 [97]	USA	E	Volunteers	76	22.6	22♀, 54♂	Construct validity- structural validity	EFA using PCA with varimax rotation performed. 3 components/factors defined: environmental sound, voice and music. BAIS-V: loading for environmental sound 0.48–0.81, for voice 0.42–0.77, for music 0.48–0.89. Total variance explained by 58%. BAIS-C: loading for environmental sound 0.55–0.82, for voice 0.44–0.73, for music 0.45–0.84. Total variance explained by 59%. Some items loaded on more than one factor but this loading <0.50.	Doubtful	?	Sample size doubtful. Some items showed instability and loaded on two factors. CFA should be conducted to confirm these three components.
									Construct validity- hypothesis testing	**Corr. BAIS (both scales) with VVIQ-M** *r*=0.62 **Know-group validity** No sig. difference between men and women on the BAIS score. Sig. difference between men and women on the VVIQ-M.	Doubtful	?	Psychometric properties of comparator instrument insufficiently reported. Participants insufficiently described. No hypotheses defined.
Betts Questionnaire Upon Mental Imagery (shorted version 35-item, SQMI)	Psy	Sheehan P. W., 1967 [98]	AU	E	Students	62	NR	62♀	Cross-cultural validity	American and Australian students compared. No sig. difference between students regarding vividness over all items established.	Inadequate	?	Low sample size. Population not described. Unclear which group difference analysis was performed.
						60	NR	28♀, 32♂	Construct validity- structural validity	*r*=0.99 between total scores based on the complete scale and the shortened form was obtained. A factor established: a general imagery ability for all sensory modalities. All 35 items in the scale loaded highly on the factor, with an average loading of 0.57	Inadequate	?	Sample size for this analysis inadequate. *Not all information reported for quality criteria rating.
Betts Questionnaire Upon Mental Imagery (shorted version 35-item, SQMI)	n.d.s.	White et al. 1974 [161]	AU	E	Students	1562	22.3♀	600♀	Construct validity- structural validity	PCA with varimax rotation; one factor with several modalities: auditory, kinaesthetic, gustatory, olfactory, organic, cutaneous, visual. Total variance explained by 51.8%. Factor loadings ranged from 0.43 to 0.89. Only one item ‘sun’ on visual subscale loaded very low (<0.20).	Adequate	?	One item on visual subscale 'sun' should be removed from questionnaire.
							20.4♂	962♂					
	n.d.s.	Lorenz & Neisser 1985 [162]	USA	E	Students	46	NR	NR	Construct validity- structural validity	PCA with varimax rotation used to extract 3 factors: Factor 1. Vividness and control, Factor 2. Spatial manipulation, Factor 3. childhood memory. Betts QMI loaded on 1^st^ factor with loading 0.81.	Inadequate	-	Sample size inadequate for this analysis.
	n.d.s.	Kihlstrom et al. 1991 [163]	USA	E	Students	2036	NR	NR	Construct validity- structural validity	PCA with orthogonal rotation showed 7 factors corresponding closely to the subscales.	Doubtful	?	#, Participants not described. *Not all information reported for quality criteria rating.
									Construct validity- hypothesis testing	**Corr. Betts QMI with GTVIC** *r*=0.25	Inadequate	?	Measurement properties of the comparator instrument not reported. The corr. with the comparison instrument that measures the same construct is missing.
	n.d.s.	Campos & Pérez-Fabello 2005 [104]	ES	S	Students	562	20.2	148♀, 414♂	Construct validity- structural validity	PCA followed by varimax orthogonal rotation identified 8 factors, together accounted for 58.4% of total variance; Factor loadings 0.42–0.79. 3 items referred to different senses loaded on the 7. factor. Item 5 loaded on the 8 factor, which was a kind of visual image.	Adequate	?	Some items seem to be unstable and could be removed. Item removed could influence the number of factors/modalities identified.
									Construct validity- hypothesis testing	**Corr. Betts QMI and GTVIC** *r*=−0.34 **Correlation Betts QMI and VVIQ** *r*=0.58	Inadequate	?	Measurement properties of the comparator instrument not reported. Corr. Betts QMI with VVIQ reported, but unclear which modality of Betts QMI has a strong corr. with VVIQ.
	n.d.s	Baranchok John 1995 [102]	USA + MX	S + E	Mexican students^1^	350	NR	159♀, 191♂	Cross-cultural validity	The *t*-test, *t*(12)=0.71, *p*>0.10, supported the null hypothesis, suggesting that there was no difference between students from the USA and Mexico. The Spanish version of the QMI seems linguistically and statistically equivalent to the English version.	Very good	+	Very good sample size and good description of study population and procedures.
					US students^2^	307		130♀, 177♂					
									Construct validity- structural validity	PCA with varimax rotation identified one general imagery factor with 7 modaliies specific factors. 51.1% of the variance was explained by the USA students and 49.9% by the Mexican. Factor loaded from students from the USA by 0.25–0.83 (only one item on visual subscale loaded <0.20) and from the Mexican students by 0.25-0.80 (one item on visual and two items on kinaesthetic loaded <0.20).	Adequate	−	Some items loaded very low. These results confirmed findings by White (1974) [161] and Campos & Péréz-Fabello 2005 [104]. Kinaesthetic subscale seems the most unstable, and item 5 on visual subscale should be evaluated again.
Clarity of Auditory Imagery Scale (CAIS)	n.d.s.	Willander & Baraldi 2010 [105]	SE	E/Se	Students	212	25.9	58♀, 154	Construct validity- structural validity	EFA and principal axis factoring was conducted and one factor was extracted. Factor loadings of 16 items ranged from 0.40 to 0.67. The total variance was explained by 31.63%.	Adequate	?	Following COSMIN recommendation EFA should be rated as adequate. CFA should be performed too. Explained variance just above 0.30.
Clarity of Auditory Imagery Scale (CAIS)									Construct validity- hypothesis testing	**Known-groups validity** No difference established between men and women (*p* > 0.05).	Doubtful	+	Results are in accordance with the hypotheses but participants characteristics insufficiently described.
	n.d.s.	Campos 2011 [106]	ES	S	Students	234	19.6	47♀, 187♂	Construct validity- structural validity	PCA with varimax orthogonal rotation was conducted. 5 factors with eigenvalues >1 identified. Factor 1 loaded by Item 5,11,12,13,14,15,16; Second factor loaded by Item 6,8,9: Third factor: Item 7 and 10; fourth factor: Item 1 and 2; Fifth factor Item 3 and 4. Factor loadings ranged 0.41–0.79. The five factors explained 57.4% of total variance.	Adequate	?	According to COSMIN recommendations EFA should be rated as adequate. EFA identified 5 factors, but factors not explained by CFA should be performed too.
									Construct validity- hypothesis testing	**Corr. CAIS with VVIQ-2** *r*=0.42 **Corr. CAIS with MASMI** *r*=−0.12 **Corr. CAIS with Bett’s QMI** visual *r*=−0.31, auditory *r*=−0.46, cutaneous *r*=−0.37, kinaesthetic *r*=−0.36, gustatory *r*=−0.42, olfactory *r*=−0.41, organic *r*=−0.25	Doubtful	?	Measurement properties of the comparator instrument insufficiently reported. Very low corr. with other measures. The corr. with the comparison instrument that measures the same construct is missing.
	Edu	Tuznik & Francuz 2019 [107]	PL	PO	Musicians	39	22.5	21♀, 18♂	Construct validity- structural validity	PCA was conducted by forcing a one-factor solution. The factor loadings of 16 items ranged from 0.46 to 0.74. All factor loadings were >0.32. The total variance was explained by 34.48%.	Doubtful	?	Doubtful sample size.
					Non-musicians	40	24.5	20♀, 20♂					
									Construct validity- hypothesis testing	**Known-group validity** Neither gender (*p*=0.372) of participants or their level of musical expertise (*p*=0.114) differentiated the scores obtained.	Very good	?	Participants characteristics well described. Not all results are in accordance with hypotheses.
Gordon Test of Visual Imagery Control (GTVIC)	n.d.s.	Lorenz & Neisser 1985 [162]	USA	E	Students	46	NR	NR	Construct validity- structural validity	PCA with the varimax rotation was used to extract 3 factors: Factor 1: Vividness and control, Factor 2: Spatial manipulation, Factor 3: childhood memory. GTVIC loaded on 1. factor with loading 0.81.	Inadequate	−	Sample size inadequate for this analysis.
	n.d.s.	Kihlstrom et al. 1991 [163]	USA	E	Students	2805	NR	NR	Construct validity- structural validity	PCA with orthogonal rotation performed twice and showed: 1. 4 factors: car in colour or not, car in normal motion or car in unusual positions or motions. 2. 2 factors: car in normal motion or car in unusual positions or motions.	Doubtful	?	#, Participants not described. Unclear factor structure: four or two? *Not all information reported for quality criteria rating.
									Construct validity- hypothesis testing	**Corr. GTVIC with Betts QMI** *r*=0.25 **Corr. GTVIC with VVIQ** *r*=0.45	Inadequate	?	No information on measurement properties of the comparator instrument available. See comment above about Betts QMI.
	n.d.s.	Lequerica et al. 2002 [22]	USA	E	Students	80	22.1	39♀, 41♂	Construct validity- hypothesis testing	**Corr. GTVIC with VMIQ visual subscale** *r*=0.72 **Corr. GTVIC with MIQ visual subscale** *r*=0.45 Sign. corr. among subjective measures of mental imagery. No corr. between objective and subjective measures of mental imagery providing evidence for the multidimensional nature of imagery.	Adequate	+	# Students received extra credits in their psychology courses for participation. Results in accordance with the hypothesis.
	n.d.s.	Pérez-Fabello & Campos 2004 [111]	ES	S	Students	479	20.5	70♀, 409♂	Construct validity- structural validity	PCA followed by varimax orthogonal rotation identified four factors. Movement, misfortune, colour, stationarity. The total variance explained by 55.6%. Factors loadings range 0.43 to 0.88.	Adequate	−	Statement of four- factor structure should be rejected. Item 6 loaded on two factors. Fewer than 3 items loaded on factor 3 and 4.
Gordon Test of Visual Imagery Control (GTVIC)	n.d.s.	Pérez-Fabello & Campos 2004 [111]	ES	S	Students	479	20.5	70♀, 409♂	Construct validity- hypothesis testing	**Corr. GTVIC with VVIQ** *r*=−0.40 **Corr. GTVIC with VVQ** *r*=0.05	Adequate	?	Authors calculated corr. between different measures (construct validity), which measured different constructs. The corr. with the comparison instrument that measures the same construct is missing.
Alternate Form of the Gordon Test of Visual Imagery Control (TVIC)	n.d.s.	Mckelvie 1992 [28]	CA	E	Students	116	NR	49♀, 67♂	Criterion validity	**Corr. GTVIC alternate form with GTVIC original** Pearson corr. *r*=0.52	Very good	−	Author calculated corr. between alternate form and original version of GTVIC, which belongs to criterion validity. However, corr. between measures <0.70.
Imaging Ability Questionnaire (IAQ)	Med	Kwekkeboom 2000 [42]	USA	E	Participants from different sources	200	48.7	NR	Construct validity- structural validity	CFA with PCA and oblique rotation was performed and two factors confirmed: absorption and image generation. Factor loadings >0.44. The corr. between two factors was *r*=0.42.	Adequate	?	Adequate sample size for factor analysis. *Not all information reported for quality criteria rating .
Imagery Questionnaire by Lane	n.d.s.	Lane 1977 [112]	CA	E	Students	320	NR	122♀, 198♂	Construct validity- structural validity	PCA with varimax rotation of modality yielded one factor: imagery control. Loadings ranged from 0.59 to 0.76. 11 factors were obtained in the component analysis of the individual items. While the composition of four of these factors approximated the content of four of the modalities, no factor completely and exclusively represented any given modality.	Doubtful	?	Insufficient information about factor analysis and quality criteria rating not possible.
						60	NR	22♀, 38♂	Construct validity- hypothesis testing	**Corr. Imagery by Lane with:** **GTVIC ***r*=0.53 **Betts QMI ***r*=0.57	Inadequate	−	Why comparison with Betts QMI, when not the same domains/constructs were investigated?
Kids Imaging Ability Questionnaire (KIAQ)	Med	Kwekkeboom et al. 2000 [113]	USA	E	Experts	3	NR	NR	Content validity	All reviewers agreed that the items adequately represented the construct of ‘imaging ability’. The content and language of items were assessed to be appropriate for 6- to 14-year-olds. The format, either self-administered or reading items to the child, was also agreed to be satisfactory.	Doubtful	?	Only 3 experts reviewed the KIAQ for relevance, comprehensiveness and comprehensibility. Target population was not considered for evaluation of content validity.
					Children	58	9.9	19♀, 39♂	Construct validity- hypothesis testing	**Corr. KIAQ with SFPI** 1. Time, *N*=54: *r*=0.31 2. Time, *N*=44: *r*=0.46	Doubtful	−	Doubtful if comparator instrument cover the same construct Corr. <0.50.
Mental Imagery Scale (MIS)	n.d.s	Dercole et al. 2010 [114]	IT	I	Participants characteristics NR	262	29.0	92♀, 170♂	Construct validity- structural validity	EFA with oblimin rotation produced six factor solution: stability, perspective, distance, level of details, dimensions, rapidity. The total variance explained by 54.6%. Factors loadings 0.52–0.80.	Doubtful	+	Sample size very good but participants not described. CFA should be performed.
Plymoth sensory imagery questionnaire (Psi-Q)	n.d.s.	Andrade et al. 2014^1^ [115]	UK	E	Students	404	NR	NR	Construct validity- structural validity	EFA with maximum likelihood extraction and oblimin rotation found seven factors with eigenvalues >1. Goodness of fit test: *χ*^2/^(371)=889. Factors loaded very strong, all >0.50 (range 0.53–0.87).	Very good	?	This article reported results from 3 studies. *Not all information reported for quality criteria rating.
Plymoth sensory imagery questionnaire (Psi-Q)	n.d.s.	Andrade et al. 2014^2^ [115]	UK	E	Students	209	NR	NR	Construct validity- structural validity	CFA with 7 factor model provided a good model fit: *χ*^2^/df=1.51, CFI=0.93, RMSEA=0.05.	doubtful	+	Accepted model fit: CFI >0.95, or SRMR <0.08, or RMSEA <0.06.
	n.d.s.	Andrade et al. 2014^3^ [115]	UK	E	Students	212	23.4 (median)	59♀, 153♂	Construct validity- hypothesis testing	**Corr. Psi-Q long version with VVIQ-2** *r*=0.67 **Corr. Psi-Q short version with VVIQ-2** *r*=0.66	Inadequate	?	Measurement properties of the comparator instrument not reported. Several modalities are covered with Psi-Q. Unclear which modality strong corr. (>0.50) with VVIQ-2.
	n.d.s.	Pérez-Fabello & Campos 2020 [116]	ES	S	Students	394	21.0	101♀, 293♂	Construct validity- structural validity	CFA for long version with 7 factor model provided a good model fit: *χ*^2^ (733.95), df=413, GFI=0.89, CFI=0.92, NNFI=0.91, RMSEA=0.04, SRMR=0.05.	Very good	+	Accepted model fit: CFI >0.95, or SRMR <0.08, or RMSEA <0.06.
									Construct validity- hypothesis testing	**Corr. Psi-Q with Betts QMI was sign.** (*p*<0.01), *r*=0.40–0.56 **Corr. Psi-Q with VVIQ was sign. (*p*<0.01)** *r*=−0.30–0.41 **Corr. Psi-Q with OSIVQ object was sign.** *r*=0.19–0.34	Doubtful	+	Measurement properties of the comparator instruments insufficiently reported. The 75 % of the results are in accordance with the hypothesis.
Sport Imagery Ability Measure (SIAM)	Sport	Watt 2003^1^ [36]	AU	E	Students	5	Range 15–16	NR	Content validity	Items were selected through examination of relevant imagery theories, analysis of research work in the field of imagery ability, and review and analysis of a number of existing measures of imagery ability, used in the areas of sport and general psychology. Students were asked about comprehensibility, professionals were asked about relevance and comprehensiveness. 6 experts reviewed all items. Comments and suggested modifications were analysed and incorporated into the final draft.	Doubtful	?	This article reported results from 4 studies. Data recording and analysis are not clearly described. Relevance, comprehensiveness and comprehensibility no evaluated by the population of interest.
					Experts	6	NR						
Revised Sport Imagery Ability Measure (SIAM-R)	Sport	Watt 2003^1^ [36]	AU	E	Students	474	18.42	268♀, 206♂	Construct validity- structural validity	EFA with oblimin rotation, two factors: 1. dimensions and visual modality; 2. modalities minus visual modality. The total variance explained by 75%. Factors loadings greater than 0.50 (0.50–0.92). Only emotion variable had no loadings greater than 0.50. 1. Factor=0.45 and 2. Factor=0.43 both the loadings for this variable were very close.	Adequate	?	This article reported results from 4 studies, 2003^1^=study 1. Subscales emotion and kinaesthetic loaded on both factors with >0.40.
	Sport	Watt 2003^2^ [36]	AU	E	Athletes and students	633	18.77	334♀, 299♂	Construct validity- structural validity	CFA performed. The model of 4 factors (visual/dimensions, body feeling, chemical, emotion/auditory) produced the best fit indices for the data. Nonetheless, the combination of the emotion and auditory variables as a latent construct was considered implausible. The three-factor model involving auditory sense grouped with the other single organ senses of taste and smell, visual/dimensions, and bodily feeling had the greatest conceptual coherence as a representation of sport imagery ability. *χ*^2^ (df)=617.63 (51), CFI=0.92, NFI=0.91, TLI=0.89, RMSEA=0.13.	Doubtful	−	2003^2^= study 2. Rotation method by CFA not described. Accepted model fit: CFI, NFI and TLI >0.95, or RMSEA <0.06.
Revised Sport Imagery Ability Measure (SIAM-R)	Sport	Watt 2003^3^ [36]	AU	E	Athletes and students	436	18.35	232♀, 204♂	Construct validity- convergent and discriminant validity	**Corr. SIAM-R with GTVIC, VMIQ-2, SQMI** All correlations between all the imagery tests and subscales were significant. Small to moderate correlations (*r*=0.27 to 0.48) were found for the SIAM control, vividness, visual, and kinaesthetic subscales with a number of the related dimension modalities variables of the other imagery measures, providing support for the convergent validity of these subscales of the SIAM. **Corr. SIAM with MAB** Very low to small correlations (*r*=0.01 to 0.20) reported between the SIAM subscales and (a) the cognitive ability measures and (b) unrelated dimension and modality variables of the other imagery measures, supporting the discriminant validity.	Very good	+	2003^3^= study 3. Appropriate sample size. The results are in accordance with the hypothesis.
	Sport	Watt 2003^4^ [36]	AU	E	Athletes	33	17.91	19♀, 14♂	Criterion validity- concurrent validity	**Corr. SIAM with CV Imagery characteristic** visual=0.04, kinaesthetic=0.13, auditory=0.29, tactile=-0.20, emotion=0.19	Inadequate	-	2003^4^= study 4. Low sample size. For criterion validity a valid measure should be considered as 'gold standard'.
Sport Imagery Ability Questionnaire (SAIQ)	Sport	Williams & Cumming 2011 [117]	UK	E	Athletes	403	20.2	198♀, 205♂	Content validity	5 sport psychology experts, who were experienced in designing questionnaires, and 5 athletes systematically examined the wording and the content of items. Content validity index was calculated.	Doubtful	?	Pilot study (SAIQ development). Results from 4 studies reported in this article. Insufficient information about test procedures: how data were collected- individually or group. Data collection regarding relevance, comprehensiveness and comprehensibility doubtful.
	Sport	Williams & Cumming 2011^1^ [117]	UK	E	Athletes	375	24.7	179♀, 196♂	Construct validity- structural validity	20-item version was evaluated. Principle axis factoring with oblimin rotation resulted in 4 factors/subscales: skill imagery, strategy imagery, goal imagery and affect imagery. Final SAIQ included 12 items with 3 item per factor. Eigenvalues ranged from 1.13–4.05, together accounting for 69.63 % of the variance.	Adequate	+	Following COSMIN recommendation EFA should be rated as adequate.
	Sport	Williams & Cumming 2011^2^ [117]	UK	E	Athletes	363	24.8	175♀, 188♂	Construct validity- structural validity	12-item version evaluated. CFA with maximum likelihood performed. The four-factor model demonstrated adequate fit model: *χ*^2^=96.19, CFI=0.96, TLI=0.95, SRMR=0.05, RMSEA=0.05. Factor loadings 0.58–0.86.	Very good	+	Accepted model fit: CFI, TLI>0.95, or SRMR<0.08, or RMSEA <0.06.
	Sport	Williams & Cumming 2011^3^ [117]	UK	E	Athletes	426	NR	199♀, 227♂	Construct validity- structural validity	Modified version (15 items and 5 subscale) evaluated. CFA with maximum likelihood performed. An adequate fit to the data was established for a final five-factor model: *χ*^2^=204.53, CFI=0.96, TLI=0.95,SRMR=0.04, RMSEA=0.06. Factor loadings 0.62-0.88.	Very good	+	Accepted model fit: CFI, TLI>0.95, or SRMR<0.08, or RMSEA<0.06.
	Sport	Williams & Cumming 2011^4^ [117]	UK	E	Athletes	220	19.5	86♀, 134♂	Construct validity- structural validity	Modified version (15 items and 5 subscale) evaluated with second population. CFA with maximum likelihood performed. An adequate fit to the data was established for a five-factor model: *χ*^2^=108.59, CFI=0.98, TLI=0.97, SRMR=0.04, RMSEA=0.04. Factor loadings 0.62–0.88.	Very good	+	Accepted model fit: CFI, TLI>0.95, or SRMR<0.08, or RMSEA<0.06.
	Sport	Williams & Cumming 2011^4^ [117]	UK	E	Athletes	220	19.5	86♀, 134♂	Construct validity- hypothesis testing	**Corr. SIAQ with MIQ-3** Small to moderate corr. ranged from 0.14–0.24 suggesting that imagery ability of movement imagery and sport imagery content are not the same trait.	Doubtful	+	Authors used term concurrent validity, but criterion validity was evaluated. The results are in accordance with the hypothesis.
Survey of Mental Imagery	n.d.s.	Switras 1978 [118]	USA	E	Students	350	NR	129♀, 221♂	Construct validity- convergent and discriminant validity	Convergent and discriminant validity supported by the fact that the corr. between both main dimensions (controllability and vividness) on the same test forms were les (discriminant) than the corr. between the same factors on the different test forms (convergent).	Doubtful	?	*Insufficient information reported for COSMIN and quality criteria evaluation.
						28	NR	NR	Construct validity- structural validity	PCA with the orthogonal varimax rotation. 7 factors were extracted: visual, olfactory, somesthetic, kinaesthetic-tactile controllability, gustatory, kinaesthetic-tactile vividness, and auditory imagery. Factors loadings greater than 0.50. Form A: 0.60–0.81. Form B: 0.58–0.82.	Inadequate	-	FA performed only with 28 subtests (14 for each form).
	n.d.s.	Grebot 2003 [119]	FR	F	Teachers	162	36.0	31♀, 131♂	Construct validity- structural validity	Factor analysis, performed on 4 modality-factor subtest scores, yielded four specific factors corresponding to 4 modalities of imagery for controllability, vividness and formation. Expanded variance for controllability ranged from 7.3–13% for all four subscales, for vividness from 8.7–14.2% and for formation from 8.0–13.9%.	Inadequate	−	Sample size for this analysis insufficient.
Visual Elaboration Scale (VES)	n.d.s.	Campos & Pérez 1988 [164]	ES	S	Students	147	19.8	60♀, 87♂	Construct validity- hypothesis testing	**Corr. VES with MEIQ (MEIQ consists of 2 parts, visual scenes and personal actions, and three scales for each part: image, absorption and effort)** ***r***= ranged from −0.28 to −0.43 for both parts and image + effort subscales. Only for subscale absorption no sign. corr. **Corr. VES with IDQ** *r*=0.21 (VES and verbal scale of IDQ) *r*=0.27 (VES and imagery scale of IDQ)	Doubtful	?	Some information about comparator instrument provided, but no information on measurement properties of the comparator instrument. Test procedures not described. No hypothesis defined. Insufficient information about comparator instrument.
Vividness of Olfactory Imagery Questionnaire (VOIQ)	n.d.s.	Gilbert et al. 1998 [121]	USA	E	Fragrance experts^a^	122	NR	63♀, 59♂	Construct validity- hypothesis testing	**Corr. VOIQ with VVIQ** Experts *r*=0.18 Non-experts *r*=0.44 **Know-groups validity** Sig. difference between experts and non-experts on the VOIQ score. No difference between men and women.	Inadequate	−	Psychometric properties of comparator instrument not reported. Corr. with comparator instrument <0.50. Participants described. Results in accordance with hypothesis
					Non-expert controls^b^	95		50♀, 45♂			Very good	+	
Vividness of Object and Spatial Imagery Questionnaire (VOSI)	n.d.s.	Blazhenkova Olesya 2016^2^ [122]	TR	NR	Students	205	21.0	95♀, 110♂	Construct validity- structural validity	CFA confirmed 2 factors: object and spatial imagery. Object items loaded above 0.45 and spatial items loaded above 0.44. Two-factor model *χ*^2^ (349)=759.30, *p*<.001, CFI=0.77, GFI=0.77, RMSEA=0.08.	Doubtful	−	Participants completed the study online. Accepted model fit: CFI and GFI >0.95, or RMSEA <0.06.
									Construct validity- hypothesis testing	**Corr. VOSI and OSIQ** object imagery *r*=0.64 spatial imagery *r*=0.45	Adequate	+	Participants completed the study online. Results are in accordance with the hypothesis.
Vividness of Visual Imagery Questionnaire (VVIQ)	n.d.s.	Rossi 1977 [123]	USA	E	Students	119	NR	NR	Construct validity- structural validity	PCA performed. A single component explained 42% of variance by first administration, and 52% variance by second. Items loaded >0.50.	Doubtful	?	Rotation method used not described. *No all information reported for quality criteria rating. Sample size doubtful.
	n.d.s.	Lorenz & Neisser 1985 [162]	USA	E	Students	46	NR	NR	Construct validity- structural validity	PCA with the varimax rotation used to extract 3 factors: Factor 1: Vividness and control, Factor 2: Spatial manipulation, Factor 3: childhood memory. VVIQ loaded on 1.factor with loading 0.78.	Inadequate	−	Sample size inadequate for this analysis.
	n.d.s.	Kihlstrom et al. 1991 [163]	USA	E	Students	2805	NR	NR	Construct validity- structural validity	PCA with orthogonal rotation performed and showed 4 factors corresponded to the 4 content clusters of the VVIQ.	Doubtful	?	#, Participants not described. *Not all information reported for quality criteria rating.
	n.d.s.	Campos et al. 2002 [124]	ES	S	Secondary school students	850	13.3	428♀, 422♂	Construct validity- structural validity	PCA with varimax orthogonal rotation confirmed a single factor, vividness of visual imagery. All items loaded over 0.50 (0.53–0.66) which explained 37 % of total variance.	Adequate	?	Test procedures only briefly reported. *Insufficient information reported for quality criteria rating.
	n.d.s.	Leboutillier & Marks 2001 [125]	UK	E	Students	198	23.86	75♀, 123♂	Construct validity- structural validity	PCA with oblique rotation confirmed 3 factors (nature scenes, person scene, shop scene) and explained variance by 58.6%.	Adequate	?	*Not all information reported for quality criteria rating.
	n.d.s.	Campos & Pérez-Fabello, 2009 [126]	ES	S	Students	279	20.1	117♀, 162♂	Construct validity- hypothesis testing	**Corr. VVIQ and Gordon Test** *r*=−0.24 **Corr. VVIQ and Betts’ QMI** *r*=0.49, **Corr. VVIQ and VVIQ-2** *r*=−0.55	Doubtful	+	Some information on measurement properties of the comparator instrument. Results are in accordance with the hypotheses.
Revised version Vividness of Visual Imagery Questionnaire (VVIQ-2)	n.d.s.	Campos & Pérez-Fabello, 2009 [126]	ES	S	Students	279	20.1	117♀, 162♂	Construct validity- hypothesis testing	**Corr. VVIQ-2 and Gordon Test** *r*=−0.23 **Corr. VVIQ-2 and Betts’s QMI** *r*=−0.54 **Corr. VVIQ and VVIQ-2** *r*=−0.55	Doubtful	+	Some information provided on measurement properties of the comparator instrument. Results are in accordance with the hypotheses.
	n.d.s.	Campos 2011 [106]	ES	S	Students	206	19.7	43♀, 163♂	Construct validity- hypothesis testing	**Corr. VVIQ-2 and VVIQ-RV** *r*=0.67 **Corr. VVIQ-2 and Betts’ QMI** *r*=−0.53 **Corr. VVIQ-2 and MASMI** *r*=0.19 **Corr. VVIQ-2 and OSIVQ** verbal scale *r*=0.07 **Corr. VVIQ-2 and OSIVQ** object imagery scale *r*=0.51 **Corr. VVIQ-2 and OSIVQ** spatial imagery scale *r*=0.04	Adequate	+	# Sufficient information provided on measurement properties of the comparator instrument. Results are in accordance with the hypothesis: high corr. with Betts’ QMI and object imagery scale of OSIVQ, low corr. with MASMI and verbal + spatial scale of OSIVQ.
Vividness of Visual Imagery Questionnaire- Revised version (VVIQ-RV)	n.d.s.	Campos 2011 [106]	ES	S	Students	206	19.7	43♀, 163♂	Construct validity- hypothesis testing	**Corr. VVIQ-RV and VVIQ-2** *r*=0.67 **Corr. VVIQ-RV and Betts’ QMI** *r*=−0.53 **Corr. VVIQ-RV and MASMI** *r*=0.16 **Corr. VVIQ-RV and OSIVQ** verbal scale *r*=0.06 **Corr. VVIQ-RV and OSIVQ** object imagery scale *r*=0.53 **Corr. VVIQ-RV and OSIVQ** spatial imagery scale *r*=0.02	Adequate	+	#Only students participated and were reimbursed with course credits. Sufficient information provided on measurement properties of the comparator instrument provided. The results are in accordance with the hypothesis (see comment above).
Vividness of Wine Imagery Questionnaire (VWIQ)	Edu	Croijmans et al. 2019 [127]	NL	E	Volunteers with experience with wine	83	40.8	71♀,12♂	Construct validity- structural validity	PCA with oblique rotation employed and suggested 3 components: smell, taste, vision. Variance was explained by 68.8%. Factor loadings for smell 0.41–0.58, for taste 0.82–0.94, for vision 0.62–0.83.	Inadequate	−	Low sample size. Instability recognisable by smell items, which loaded on 2 factors (smell and taste)!
									Construct validity- hypothesis testing	**Corr. VWIQ with PSI-Q** smell *r*=0.36, taste=0.43, vision *r*=0.51 **Corr. VWIQ-vision with VVIQ** *r*=−0.51 **Corr. VWIQ-smell with VOIQ** *r*=−0.43	Inadequate	−	No description of participants. No information about the measurement properties of comparator instrument. Not all results are in accordance with the hypotheses.
**b. Assessments of mental rotation**													
Cube-Cutting Task (CCT)	n.d.s.	Lorenz & Neisser 1985 [162]	USA	E	Students	46	NR	NR	Construct validity- structural validity	PCA with the varimax rotation used to extract 3 factors: Factor 1: Vividness and control, Factor 2: Spatial manipulation, Factor 3: childhood memory. Cube loaded on 2. factor with loading 0.86.	Inadequate	−	Sample size inadequate for this analysis.
	n.d.s.	Richardson 1977 [165]	UK	E	Students	60	19.0 (male)	26♀	Construct validity- hypothesis testing	Sig. corr. for male established for: CCCT and Rated Imagery Vividness *r*=0.68 CCT and MPFB *r*=0.42 CCT and Paper Folding *r*=0.43 CCT and Controllability of Imagery *r*=0.36 CCT and Personal Reaction Inventory *r*=−0.41 Sig. corr. for female established for: CCT and Rated Imagery Vividness *r*=0.56 CCT and Necker Cube Fluctuations *r*=0.46 CCT and Memory for Designs *r*=0.34 CCT and Concealed Figures *r*=0.36 CCT and MPFB *r*=0.35	Inadequate	?	No information on measurement properties of the comparator instrument. No hypothesis defined. Insufficient information about comparator instrument.
							20.0 (female)	34♂					
	n.d.s.	Lequerica et al. 2002 [22]	USA	E	Students	80	22.1	39♀, 41♂	Construct validity- hypothesis testing	**Corr. CCT with MRT** *r*=0.58 **Corr. CCT with PFT** *r*=0.47 **Corr. CCT with JOLO** *r*=0.40 **Corr. CCT with HVOT** *r*=0.50 **Corr. CCT with WAIS-R** *r*=0.59	Inadequate	+	No information on measurement properties of the comparator instrument. The results are in accordance with the hypothesis: no sig. corr. between subjective and objective measures of mental imagery.
German Test of the Controllability of Motor Imagery in older adults (TKBV)	n.d.s.	Schott 2013 [29]	DE	G	Healthy	195	57.3	102♀, 93♂	Construct validity- structural validity	EFA with with the orthogonal varimax rotation showed two- factor structure: recognition and free recall. Total variance explained by 42%. Factors loaded ranged from 0.57–0.85.	Adequate	−	Adequate methodological quality because no CFA performed. Variance explained by two factors < 50%.
									Construct validity- hypothesis testing	**Corr. TKBV Recognition and TUG** *r*=−0.31 **Corr. TKBV Recognition and MIQ visual** *r*=0.143 **Corr. TKBV Recognition and MIQ kinaesthetic** *r*=0.13 **Corr. TKBV Free recall and TUG** *r*=−0.33 **Corr. TKBV Free recall and MIQ visual ***r*=0.14 **Corr. TKBV Free recall and MIQ kinaesthetic** *r*=0.11 No gender difference established.	Doubtful	?	Some information about comparator instrument provided, but no information on measurement properties of the comparator instrument. No hypothesis defined.
									Construct validity-hypothesis testing	**Corr. TKBV Recognition with Corsi block tapping test** *r*=0.45 **Corr. TKBV Free recall with Corsi block tapping test** *r*=0.38 **Corr. TKBV Recognition with physical activity** *r*=0.50 **Corr. TKBV Free recall with physical activity** *r*=0.36	Very good	−	Low corr. with comparator instrument <0.50.
Left/Right Judgements (LRJ)	Med	Bray & Mosley 2011 [129]	AU	E	Patients with back pain^a^	5	46.0	1♀, 4♂	Construct validity- hypothesis testing	**Know-groups validity** Patients with back pain made more errors overall than controls (*p*<0.015). The patients made more mistakes on the trunk rotation judgement task than on the hand judgement task (*p*<0.001).	Doubtful	+	Results are in accordance with hypothesis. However, sample size very small.
					Healthy^b^	5	40.0	2♀, 3♂					
	n.d.s.	Wallwork et al. 2013 [166]	AU	E	Volunteers	1737	40.0	520♀, 1130♂	Construct validity- hypothesis testing	**Know-groups validity** Response time increased with age, was greater in females than in males and was greater in left-handers than in right-handers (*p*<0.001). Accuracy reduced with age (*p*<0.001), but was unaffected by gender or handedness (*p*=0.493).	Very good	?	Sample size very good but gender imbalance (much more female participants than males). That should be taken into account for a know-groups-validity analysis.
Left/Right Judgements (LRJ)	Med	Bowering et al. 2014 [167]	AU	E	Patients with back pain + healthy	1008	37.0	324♀, 684♂	Construct validity- hypothesis testing	**Know-groups validity** Response time was not affected by back pain status. Patients who had back pain at the time of testing were less accurate than pain-free controls (*p*=0.027), as were patients who were pain free but had a history of back pain (*p*<0.01).	Doubtful	−	Insufficient description of participants (both groups) characteristics. Results are not in accordance with hypothesis.
	n.d.s.	Zimney et al. 2018 [130]	USA	E	Students	50	24.3	15♀, 35♂	Criterion validity	**Corr. card based with tablet version LRJ** Accuracy left *r*=0.46 Accuracy right *r*=0.26 RT *r*=0.78	Very good	?	Corr. between card-based version and ‘gold standard’ only for response time >0.70. Should be evaluated with a larger sample size.
	n.d.s.	Williams et al. 2019^1^ [131]	AU	E	Healthy	20	55.3	5♀, 15♂	Criterion validity	**Corr. between tablet and desktop version** Hand judgements ICC=0.84 for RT and ICC=0.91 for accuracy	Doubtful	+	Sample size could be doubtful for both studies. However, corr. between tablet version and desktop as ‘gold standard’ very good.
	n.d.s.	Williams et al. 2019^2^ [131]	AU	E	Healthy	37	38.5	9♀, 28♂	Criterion validity	**Corr. between tablet and desktop version** Back, foot, and neck judgements ICC=0.88 for RT and ICC=0.78 for accuracy	Doubtful	+	
Map Rotation Ability Test (MRAT)	n.d.s.	Campos & Campos-Juanatey 2020 [133]	ES	S	Students	257	19.7	86♀, 171♂	Construct validity- hypothesis testing	**Corr. MRAT with MRT** *r*=0.42 **Corr. MRAT with MASMI** *r*=0.40 **Corr. MRT with SOST** *r*=0.35 **Corr. MRAT with VVIQ** *r*=0.08	Doubtful	+	Some information on measurement properties of the comparator instrument reported. Structural validity not mentioned. Results are in accordance with hypothesis.
Mental Rotation of Three-Dimensional Objects (MRT)	n.d.s.	Vandenberg & Kuse 1978 [136]	USA	E	Students	312	NR	115♀,197♂	Construct validity- hypothesis testing	**Corr. Mental Rotation with spatial relation ***r*=0.50 **Corr. Mental Rotation with Chair-Window ***r*=0.45 **Corr. Mental Rotation with Identical Blocks ***r*=0.54	Inadequate	?	No information on constructs measured by the comparator instrument. No information on measurement properties of the comparator instrument.
Measure of the Ability to Form Spatial Mental Imagery (MASMI)	n.d.s.	Campos 2009 [96]	ES	S	Students	138	20.1	63♀, 75♂	Construct validity- hypothesis testing	**Corr. MASMI and PMA** *r*=0.44 **Corr. MASMI and VVIT** *r*=0.14 **Corr. MASMI and GTVIC** *r*=0.02 **Corr. MASMI and VVIQ** *r*=−0.15 **Corr. MASMI and VVIQ-2** *r*=0.13 **Corr. MASMI and Betts’ QMI** *r*=−0.02	Adequate	?	Some information on measurement properties of the comparator instrument provided. Structural validity not mentioned. Corr. between tests calculated but no hypotheses defined.
	n.d.s.	Campos& Campos-Juanatey 2020 [137]	ES	S	Students	281	19.8	97♀, 184♂	Construct validity- hypothesis testing	**Corr. MASMI with MRT** *r*=0.42 **Corr. MASMI with OSVIQ** object *r*=-0.06. spatial *r*=0.38, verbal *r*=-0.09 **Corr. MASMI with SOST** *r*=0.35	Doubtful	?	Some information on measurement properties of the comparator instrument provided. Structural validity not mentioned. Not all results are in accordance with hypotheses.
Measure of the Ability to Rotate Mental Images (MARMI)	n.d.s.	Campos 2012 [139]	ES	S	Students	354	19.5	45♀, 309♂	Construct validity- hypothesis testing	**Corr. MARMI with MRT** *r*=0.40 **Corr. MARMI with PMA** *r*=0.38 **Corr. MARMI with MASMI** *r*=0.48 **Corr. MARMI with VVIQ-2** *r*=0.10 Sign. difference between women and men (*p*<0.05). Men obtained sig. higher image rotation scores than women.	Doubtful	?	Some information about comparator instrument provided, but no information on measurement properties of the comparator instrument. Not all results are in accordance with hypotheses.
**c. Assessments of mental imagery to distinguish between different types of imagers**													
Object-Spatial Imagery Questionnaire (OSIQ)	n.d.s.	Blajenkova et al. 2006^1^ [34]	USA	E	Students	25	NR	NR	Content validity	Student interviewed about all items from the OSIQ. 3 experts in the field of mental imagery reviewed the OSIQ object and spatial items. Agreement among judges was 97%.	Doubtful	?	This article reported results from 4 studies. No details reported about interviews. Unclear if students were asked about relevance, comprehensiveness and comprehensibility.
					Experts	3							
	n.d.s.	Blajenkova et al. 2006^2^ [34]	USA	E	Students	164^a^	range (18-50)^a^	63♀, 83♂^a^	Construct validity- hypothesis testing	**Corr. OSIQ object with:** Paper Folding *r*=-0.10 Vandenberg-Kuse *r*=0.11 DTP *r*=0.19 VVIQ *r*=0.48 **Corr. OSIQ spatial with**: Paper Folding *r*=0.22 Vandenberg-Kuse *r*=0.26 Degraded Pictures *r*=0.05 VVIQ *r*=0.18	Doubtful	-	^a^= study 2a. Corr. between OSIQ object and Degraded Pictures as well as VVIQ was sign. but <0.70. Corr. between OSIQ spatial and Paper Folding as well as Vandenberg-Kuse was sign. but <0.50.
						49^b^	Range 17–47^b^	19♀, 30♂^b^	Construct validity- hypothesis testing	**Corr. OSIQ object with:** Paper Folding *r*=-0.33 Vandenberg-Kuse *r*=-0.19 Spatial Imagery Test *r*=-0.24 DPT *r*=0.31 **Corr. OSIQ spatial with**: Paper Folding *r*=0.51 Vandenberg-Kuse *r*=0.49 Spatial Imagery Test *r*=0.47 Degraded Pictures *r*=-0.05	Doubtful	-	^b^= study 2b Sample size doubtful, stronger corr. found as in study 2a. Sign. corr. between OSIQ object and Degraded Pictures was established. But corr. was very weak <0.50. Sign. corr. between OSIQ spatial and another measures for spatial imagery was established. But also very weak <0.50.
	n.d.s.	Blajenkova et al. 2006^3^ [34]	USA	E	Students	45	Range 18–30	18♀, 27♂	Construct validity: discriminant validity	**Corr. OSIQ object with:** APM *r*=-0.24 WAIS: Similarities *r*=-0.00 Advanced Vocabulary *r*=-0.12 **Corr. OSIQ spatial with:** APM *r*=0.20 WAIS: Similarities *r*=-0.20 Advanced Vocabulary *r*=-0.25	Doubtful	+	Sample size doubtful. OSIQ scales did not sig. correlate with measures of verbal and non-verbal intelligence. The results are in accordance with the hypothesis.
	n.d.s.	Blajenkova et al. 2006^4^ [34]	USA	E	Visual artists	28	NR	11♀, 17♂	Construct validity- hypothesis testing	**Know-groups validity** Visual artist scored higher than scientists and humanities professionals did on objects imagery scale. Scientists scored higher than visual artists and humanities professionals did on the spatial scale.	Doubtful	+	Authors used a term 'criterion validity', although the relationship between imagery abilities among different professions (subgroups) was investigated. However, characteristics of the group poorly described. The results are in accordance with the hypothesis.
					Natural scientists	24		19♀, 5♂					
					Humanities professionals	23		9♀, 14♂					
Object-Spatial Imagery and Verbal Questionnaire (OSVIQ)	n.d.s.	Blazhenkova & Kozhevnikov^1^ [35]	USA	E	Experts	3	NR	NR	Content validity	3 experts reviewed the verbal items with regard to their relevance to verbal cognitive style. After excluding all of the items on which there was a disagreement between the judges, items were administered to a sample of 166 students.	Doubtful	?	This article reported results from 2 studies. No details reported about interviews. Not clear if students were asked about relevance, comprehensiveness and comprehensibility? Expert asked only about relevance.
					Students and professionals from different fields	625	24.0	251♀,374♂	Construct validity- structural validity	First PCA revealed 18 factors with eigenvalues above 1. Only three factors (object, spatial, verbal imagery), had eigenvalues markedly higher than the others. These first 3 factors explained 31.95% of the variance. Based on the results from the initial PCA, a second PCA with varimax rotation was performed. The 45 OSIVQ loaded from 0.13–0.73.	Adequate	−	# Several factors loaded lower than 0.45 and variance explained by factors <50%.
	n.d.s.	Blazhenkova & Kozhevnikov 2009^2^ [35]	USA	E	Students	128	24.0	93♀,35♂	Construct validity- structural validity	Confirmatory factor analysis: the estimated three-factor model, and values of fit suggest that the three-factor model fits the data well. Model three-factor, *χ*^2^=27.61, df=24.00, *p* value=0.28, *χ*^2^/df= 1.15, CFI=0.97, RMSEA=0.03.	Inadequate	?	Sample size not appropriate for this analysis. Accepted model fit: CFI>0.95, or RMSEA <0.06. But several factors from previously PCA loaded very low.
									Construct validity- hypothesis testing	**Corr. OSIVQ spatial with spatial measures** PFT *r*=0.47 and with MRT *r*=0.31. OSIVQ verbal positiv corr. **Corr. OSIVQ verbal with verbal measures**: arranging words *r*=0.17 and with SAT verbal *r*=0.20. OSIVQ object positiv corr. **Corr. OSIVQ object with VVIQ*r*=0.41**	Doubtful	+	Some information on measurement properties of the comparator instrument reported. The results are in accordance with the hypothesis.
	n.d.s.	Campos & Pérez-Fabello 2011 [168]	ES	S	Students	213	19.6	62♀,151♂	Construct validity- structural validity	First analysis was PCA with varimax rotation and 13 factors identified, but only 3 factors had eigenvalues above 3.0 and explained 33.1% of the variance. A second three-factor forced PCA with varimax rotation was performed. Factor loadings was 0.07–0.80.	Inadequate	−	Sample size not appropriate for this analysis. Several factors loaded very low and variance explained by factors < 50%.
Paivio’s Individual Differences Questionnaire (IDQ, 86 items)	n.d.s.	Paivio & Harshman 1983 [141]	CA	E	Students	713	NR	NR	Construct validity- structural validity	FA with the oblique, 6 factor model (six factor: good verbal expression fluency, habitual use of imager, concern with correct use of words, self-reported reading difficulties, use of images to solve problems, vividness of daydreams/ dreams) provided a better fit to the data than the two-factor model.	Adequate	?	Data were collected in 1968 and 1970 with two samples. Finally data from 713 students analysed (collected in both years) but no details about samples available. *Insufficient data for quality criteria rating proposed by COSMIN.
Paivio’s Individual Differences Questionnaire (shorted IDQ, 34 items)	n.d.s.	Kardash et al. 1986 [142]	USA	E	Students	189	NR	99♀, 90♂	Construct validity- structural validity	CFA with the oblique five-factor model (factors: good verbal expression fluency, habitual use of imagery, concern with correct use of words, self-reported reading difficulties, vividness of daydreams, dreams) provided highest values: *χ*^2^=811.36, df=517, AGFI=0.77. Variance was explained by 71–77 %. Factor loadings 0.25–0.80. Only on item <0.25.	Adequate	−	AGFI value>0.95. Several factors loaded lower than 0.45.
Revised Paivio’s Individual Differences Questionnaire (IDQ, 72 items)	n.d.s.	Hiscock 1978^2^ [109]	USA	E	Students	123	NR	55♀, 68♂	Construct validity- hypothesis testing	**Corr. IDQ imagery scale with:** GTVIC *r*=0.21 Betts QMI visual scale *r*=0.49 Betts QMI auditory scale *r*=0.21 Marlowe-Crowne scale did not exceed *r*=0.11.	Doubtful	−	This article reported results from 4 studies. Construct measured by the comparator instrument unclear. The corr. with the comparison instrument that measures the same construct is missing.
	n.d.s.	Hiscock 1978^3^ [109]	USA	E	Students	79	NR	36♀, 43♂	Construct validity- hypothesis testing	**Corr. IDQ imagery scale with:** **GTVIC ***r*=0.56 **Betts QMI visual scale ***r*=0.46 **Betts QMI auditory scale ***r*=0.24 **Corr. Betts QMI visual scale with GTVIC** *r*=0.47	Inadequate	−	Construct measured by the comparator instrument not clear and measurement properties of the comparator instrument not reported. See comment above. Two measures (Visual Memory Scale and Visual Manipulation Scale) developed specifically for use in the present study.
Revised Paivio’s Individual Differences Questionnaire (IDQ, 86 items)	n.d.s.	Hiscock 1978^4^ [109]	USA	E	NR	81	NR	81♀	Construct and criterion validity	**Corr. IDQ imagery scale with Study of Values** *r*=0.35 **Corr. IDQ verbal scale with Quick Word Test** *r*=0.41	Inadequate	−	Different validity terms may be misunderstood in this study: construct and criterion validity. Author described the aim of the study as assessing of construct validity (various tests were correlated, but did not mention what was expected). However, the author used same measures to predict the findings, which is a part of criterion and not construct validity. The relevance of this study doubtful.
Sussex Cognitive Styles Questionnaire (SCSQ	n.d.s.	Mealor et al. 2016^1^ [143]	UK	E	Students	1542	27.0	586♀, 956♂	Construct validity- structural validity	EFA with an oblique rotation suggesting a six factor solution: imagery ability, technical /spatial, language and word forms, need for organisation, global bias, systemising tendency. The reduced version of the questionnaire contained 60 items, which explained 32% of total variance. Factor loading ranged from 0.31 to 0.74.	Adequate	?	2016^1^=study 1. Several items loaded <0.50. These items should be considered for deletion. CFA should be performed.
									Construct validity- hypothesis testing	**Know-groups validity** Females scored higher on imagery ability and males scored higher on technical/spatial.	Doubtful	?	Participant's characteristics insufficiently described and not all results are in accordance with hypothesis.
	n.d.s.	Mealor et al. 2016^3^ [143]	UK	E	Volunteers	121	35.0	24♀,97♂	Construct validity- hypothesis testing	**Know-groups validity** Females scored higher on imagery ability, and males scored higher on both technical/spatial, and systemising tendency. The differences observed between grapheme-colour and sequence-space synaesthetes on SCSQ scales shows that different forms of synaesthesia may predict different aspects of cognition.	Very good	?	2016^3^=study 3. Participants with equence-space synaesthesia, or grapheme-colour synaesthesia or with both. Participants characteristics described but not all results are in accordance with hypothesis.
Verbalizer-Visualiser Questionnaire (VVQ)	n.d.s.	Campos et al. 2004 [145]	ES	S	Students	969	14.2	496♀, 473♂	Construct validity- structural validity	PCA with varimax orthogonal rotation yielded 5 factors: 1. Factor= interest in words, 2. Factor= dream vividness and frequency, 3. Factor= verbal fluency, 4. Factor= task performance difficulty, 5. Factor= ways of thinking and acting. Factors loaded 0.43–0.77. This test does not have a clear factorial structure.	Adequate	−	Only high school students tested. Not all information reported for quality criteria rating. But this finding is in contrast with findings from previous studies, that obtained only 2 factors.
									Construct validity- hypothesis testing	**Corr. VVQ with GTVIC** *r*=0.08	Inadequate	−	No information on the measurement properties of the comparator instrument. Corr. found was very weak. It was expected. But the corr. with the comparison instrument that measures the same construct is missing.
	n.d.s.	Wedell et al. 2014 [146]	DE	G	Volunteers	476	24.1	99♀, 377♂	Construct validity- structural validity	FA and varimax rotation yielded 2 factors: visualizer and verbalizer. However, a large deviation between original and translated version was established. 7 items cannot clearly be attributed to one of the both factors.	Adequate	?	Quality criteria for good measurements properties cannot be rated.
**d. Assessments of use of mental imagery**													
Children’s Active Play Imagery Questionnaire (CAPIQ)	Sport	Cooke et al. 2014^1^ [147]	CA	E	Experts	7	NR	NR	Content validity	The assessment of item-content relevance and comprehensiveness was conducted by experts. Target population was not involved in this step. Not clear if data were analysed by 2 researchers independently.	Doubtful	?	Relevance, comprehensiveness and comprehensibility not evaluated in this phase.
	Sport	Cooke et al. 2014^2^ [147]	CA	E	Children	302	10.0	145♀, 157♂	Construct validity- structural validity	PCA with oblimin rotation identified a three-factor solution with 11 items. Factor 1=capability imagery. Factor 2=social imagery. Factor 3=fun imagery. The variance was explained by 61.4%. The interfactor correlations were low to moderate (1+2 *r*=0.23, 1+3 *r*=0.30, 2+3 *r*=0.44).	Adequate	?	Very good sample size. Factors loading not reported.
Children’s Active Play Imagery Questionnaire (CAPIQ)	Sport	Cooke et al. 2014^3^ [147]	CA	E	Children	252	10.4	118♀, 134♂	Construct validity- structural validity	CFA with three-factor model provided acceptable model fit: CFI=0.95, NFI=0.92, TLI=0.93, RMSEA=0.07.	Very good	−	Accepted model fit: CFI>0.95, or SRMR<0.08, or RMSEA<0.06 Almost all fits just below cut-off.
									Construct validity- hypothesis testing	**Known-group validity** No significant effects were noted between age (7–10 and 11–14) and for any of the imagery functions. Significant main effect for gender was found for capability imagery, (*p*=0.052), with females reporting more use of this imagery function.	Doubtful	?	Insufficient description of participants characteristics. Not all results are in accordance with hypothesis.
	Sport	Kashani et al. 2017 [148]	IR	Pe	Students	190	11.5	85♀, 85♂	Construct validity- structural validity	CFA based on the structural equation mode confirmed three-factor model with acceptable model fit: *χ*^2^=88.59, df=41, CFI=0.94, TLI=0.93, RMSEA=0.08.	Very good	−	Accepted model fit: CFI>0.95, or SRMR<0.08, or RMSEA<0.06 Almost all fits just below cut-off.
Exercise Imagery Questionnaire-Aerobic Version (EIQ-AV)	Sport	Hausenblas et al. 1999^2^ [149]	CA	E	Experts	3	NR	NR	Content validity	3 exercise professionals and 3 exercise participants commented on the wording, phraseology, and scoring of the questionnaire items. Minor revisions were made to the questionnaire items based on their comments.	Doubtful	?	This article reported results from 3 studies. No information whether experts and athletes were asked about relevance and comprehensiveness and how data were analysed.
					Athletes	3							
					Athletes	307^1^	22.9^1^	9♀,296♂^1^	Construct validity- structural validity	PCA with varimax rotation conducted for each sample to reduce items. From this analysis a three-factor structure emerged accounting for 63.8% of the variance in sample 1 and 67.6% of the variance in sample 2. The three factors are: energy, appearance, and technique.	Very good	?	*Insufficient information (e.g. factors loading) reported for quality criteria rating.
					Athletes	171^2^	22.4^2^	3♀,168♂^2^					
		Hausenblas et al. 1999^3^ [149]	CA	E	Athletes^a^	144	22.0	16♀,128♂	Construct validity- structural validity	CFA was conducted. Some items were removed. The revised model yielded good fit indices: Athletes^a^: *χ*^2^=40.5, *χ*^2^/df=1.69, RMSR=0.05, SRMSR=0.05, GFI=0.94, AGFI=0.89, NFI=0.92, NNFI=0.95, GFI=0.97. Athletes^b^: *χ*^2^=49.6, *χ*^2^/df=2.06, RMSR=0.05, SRMSR=0.05, GFI=0.96, AGFI=0.93, NFI=0.95, NNFI=0.96, GFI=0.97. Finally, version consists of 9 items.	Very good	+	Very good sample size. Steps of data analysis very clear described. Accepted model fit: CFI, TLI>0.95, or SRMR<0.08, or RMSEA<0.06.
					Athletes^b^	267	22.4	5♀,262♂					
	Sport	Pérez-Fabello & Campos 2020 [150]	ES	S	Students	166	20.1	127♀,39♂	Construct validity- structural validity	CFA and two-factor model (only factors energy and technique, the factor appearance was eliminated) revealed a better fit indicates: *χ*^2^ (df=8)=14.95, GFI=0.97, CFI=0.97, NNFI=0.94, RMSEA=0.07, SRMR=0.04.	Very good	+	Accepted model fit: CFI, TLI>0.95, or SRMR<0.08, or RMSEA<0.06.
									Construct validity- hypothesis testing	Sign. corr. among the three EIQ scales: technique with appearance imagery *r*=0.52, technique with energy imagery *r*=0.56, energy with appearance imagery *r*=0.48 No corr. found between EIQ and MIQ-R, VMIQ, or VVIQ. Only low corr. (*r*=0.26) was found between EIQ technique and GTVIC.	Very good	−	Most of the results are not in accordance with the hypothesis.
Sport Imagery Questionnaire (SIQ)	Sport	Hall et al. 1998^1^ [151]	CA	E	Experts	4	NR	NR	Content validity	4 research experts, in the area of sport psychology and 4 in cognitive psychology assessed content validity. The content, format, wording of the items and usage within athletic populations were determined and evaluated by experts.	Doubtful	?	This article reported results from 3 studies. No details reported about interviews, insufficient information about data analysis. Unclear whether athletes were asked about relevance, comprehensiveness and comprehensibility.
	Sport	Hall et al. 1998^1^ [151]	CA	E	athletes	113	23.6	53♀,60♂	Construct validity- structural validity	**46-item version** PCA and maximum likehood with oblique rotation was employed. MG was separated in two different factors: represent two distinct subscales: MG-A= motivational general arousal and MG-M= motivational general mastery.	Inadequate	?	Sample size for this analysis not appropriate. Quality criteria for good measurements properties cannot be rated.
	Sport	Hall et al. 1998^2^ [151]	CA	E	Students	161	NR	NR	Construct validity- structural validity	**30-item version, 5 scales** PCA and maximum likelihood with oblique rotation was employed. Results showed that the items loaded very cleanly onto 5 factors (cognitive general, cognitive specific, motivational specific, motivational general arousal, motivational general mastery) and all items loaded above the criterion level (>0.35). Factors loading ranged from 0.45–0.97.	Adequate	?	EFA performed. Sample size doubtful. Variance explained by factors not reported.
	Sport	Hall et al. 1998^3^ [151]	CA	E	Athletes	271	NR	184♀,87♂	Construct validity- structural validity	**30-item version, 5 scales** PCA revealed the existence of 5 distinct factors: cognitive general, cognitive specific, motivational specific, motivational general arousal, motivational general mastery. Factors loaded >0.45. Total variance explained by 57.5%.	Adequate	+	EFA with adequate sample size performed.
	Sport	Vurgun et al. 2012 [152]	TR	Tu	Athletes	142	21.8	100♀,42♂	Construct validity- structural validity	EFA and varimax rotation determined 30 items and 5 factors. The explained variance was by 65.48%. CFA with maximum likelihood estimation method performed and the model found with the EFA showed a good fit to the data: *χ*^2^ (395)=632.55, GFI=0.77, CFI=0.88, NNFI=0.87, RMSEA=0.06, SRMR=0.07.	Inadequate	+	Sample size inadequate for this analysis. Accepted model fit: CFI, TLI>0.95, or SRMR<0.08, or RMSEA<0.06.
	Sport	Ruiz & Watt 2014 [153]	Not clear	S	Athletes	361	24.1	234♀,29♂	Construct validity- structural validity	The CFA representing the 30-item 5 factor SIQ model revealed acceptable fit to the data, *χ*^2^ (378)=694.60; CFI=0.91; TLI=0.90; RMSEA=0.05; SRMR=0.05). Factors loaded 0.41-0.83.	Very good	+	Accepted model fit: CFI, TLI>0.95, or SRMR<0.08, or RMSEA<0.06.
Sport Imagery Questionnaire for Children (SIQ-C)	Sport	Hall et al. 2009^1^ [154]	CA	E	Young athletes	428	10.9	137♀,291♂	Construct validity- structural validity	CFA approached a reasonable fit for the hypothesised five-factor model; Q=3.08, CFI=0.89, GFI=0.89, RMSEA=0.07.	Doubtful	-	This article reported results from 3 studies. Rotation method not described. Accepted model fit: CFI, TLI>0.95, or SRMR<0.08, or RMSEA<0.06.
	Sport	Hall et al. 2009^2^ [154]	CA	E	Young athletes	628	NR	283♀,345♂	Construct validity- structural validity	CFA performed, with a five-factor model of imagery use being hypothesised: (Q=3.33, CFI=0.89, GFI=0.91, RMSEA=0.06) indicated that the measurement model was tenable.	Doubtful	-	Rotation method not described. Model fits were at the limit. Accepted model fit: CFI, TLI>0.90, or RMSEA<0.10.
	Sport	Hall et al. 2009^3^ [154]	CA	E	Young athletes	82	11.5	21♀,61♂	Construct validity- hypothesis testing	Corr. for MG-M and self-confidence *r*=0.73 and for MG-M and self-efficiency *r*=0.61. Corr. for CS imagery and self-confidence *r*=0.39 and self-efficacy *r*=0.41, CG imagery and self-confidence *r*=0.38 and self-efficacy *r*=0.38.	Adequate	+	Confidence was measured with the CSAI-2, self-efficacy with the SEQ-S. Some information on measurement properties of comparator instrument provided. Results are in accordance with the hypothesis.
Spontaneous Use of Imagery Scale (SUIS)	n.d.s.	Nelis et al. 2014 [156]	UK	E/ D	Students^a^	491	18.6	88♀,403♂	Construct validity- structural validity	EFA in group a suggested two components. CFA was conducted in groups b and c evaluating a one- and two-factor model. The one-factor model was accepted as final for the following reasons: Fit indices did not strongly differ between the two models, and in the two-factor model, the factors were highly correlated. Fit indices group b: CFI: 0.93. TLI=0.92, RMSEA=0.06, *χ*^2^=115 .50 df=54, *p*<.001. Factor loadings 0.35–0.98. 2 Items 1 and 6 did not reach 0.30. Fit indices group c: CFI: 0.91. TLI=0.89, RMSEA=0.07, 174.19, df=54, *p*<.001.Factor loadings 0.40–0.71. 2 items 1 and 6 did not reach 0.30.	Very good	+	# Very good sample size. The steps of data analysis very clearly described. Accepted model fit: CFI, TLI>0.95, or SRMR<0.08, or RMSEA<0.06.
					Volunteers^b^	373	34.9	119♀,254♂					
					Students^c^	433	18.4	82♀,351♂					
									Construct validity- hypothesis testing	**Corr. SUIS with VVIQ** *r*(350)=−0.35, *p*<.001 **Corr. SUIS with visual subscale of the QMI** *r*(338)=−0.38, *p*<.001.	Doubtful	+	The results are in accordance with hypothesis. Incomplete information on measurement properties of the comparator instrument.
	n.d.s.	Görgen et al. 2016^1^ [157]	DE	G	Students	216	23.7	60♀,156♂	Construct validity- structural validity	CFA one-factor model revealed acceptable fit indices: *χ*^2^ (df=54)=86.91, *p*<.01, RMSEA=0.05, CFI=0.92, TLI=0.90. Factor loadings 0.21–0.64. One item (item 6) reach −0.05.	Very good	-	This article reported results from two studies. Good sample size. Several factors loaded very low. Accepted model fit: CFI, TLI>0.95, or SRMR<0.08, or RMSEA<0.06.
									Construct validity- hypothesis testing	**Corr. SUIS with TABS** *R*=0.43, *p*<0.001 **Corr. SUIS with RSQ** *r*=0.14, *p*<0.05	Adequate	?	Sufficient information on measurement properties of the comparator instrument. Very low corr., no hypothesis defined. Insufficient information about comparator instrument.
	n.d.s.	Görgen et al. 2016^2^ [157]	DE	G	Students	447	24.9	161♀,286♂	Construct validity- structural validity	**SUIS 17-item version** CFA one-factor model revealed acceptable fit indices: *χ*^2^ (df=119)=413.71, *p*<.001, RMSEA=0.07, CFI=0.92, TLI=0.91.Factor loadings 0.26–0.73.	Very good	−	Very good sample size. One factor loaded <0.40. Accepted model fit: CFI, TLI>0.95, or SRMR <0.08, or RMSEA<0.06.
	n.d.s.	Görgen et al. 2016^2^ [157]	DE	G	Students	447	24.9	161♀,286♂	Construct validity- hypothesis testing	**Corr. SUIS 17-item with STAI-T** *r*=0.16, *p*<0.01 **Corr. SUIS 17-item with TABS** *r*=0.42, *p*< 0.001	Adequate	?	Sufficient information on measurement properties of the comparator instrument. Very low corr., no hypothesis defined. Insufficient information about comparator instrument.
	n.d.s.	Tanaka et al. 2018^1^ [158]	JP	J	Students	126	20.6	66♀,60♂	Construct validity- structural validity	CFA and single-factor model was performed. The model fit indices are marginally acceptable: RMSEA=0.09, GFI=0.88, AGFI=0.82, CFI=0.66.	Doubtful	-	Rotation methods for CFA not described. Accepted model fit: CFI, TLI>0.95, or SRMR<0.08, or RMSEA<0.06.
	n.d.s.	Tanaka et al. 2018^2^ [158]	JP	J	Patients with SAD	20	30.9	12♀,8♂	Construct validity- hypothesis testing	**Know-groups validity** No significant difference in mean SUIS-J score between patients with SAD (38.7, SD=5.06) and healthy controls (36.1, SD=6.9), *p*=0.92.	Very good	?	2018^2^=study 2. SAD=social anxiety disorder. Assumable that data from healthy participants from study 1 were analysed. No hypothesis defined.

Legend: The superscript numbers were used to distinguish the results per group

Disciplines in which field the tool was evaluated: *Edu* education, Med medicine, *Psy* psychology, *n.d.s.* not disciplines specific, healthy participants/students

Language of the tool, *E* English, *F* French, *G* German, *D* Dutch, *I* Italian, *S* Spanish, *Se* Swedish, *Tu* Turkish

Country abbreviations: *AU* Australia, *CA* Canada, *DE* Germany, *ES *Spain, *FR* France, *IR* Iran, *IT* Italy, *JP* Japan, *MX* Mexico, *NL* Netherlands, *SE* Sweden, *TR* Turkey, *PL* Poland, *UK* United Kingdom, *USA* United States of America

Advanced Vocabulary Advanced Vocabulary Test, *AGFI* adjusted goodness of fit index, *APM* Advanced Progressive Matrices, *CFA* confirmatory factor analysis, *CI* confidence interval, *CFI* Comparative fit index, *corr.* correlation, *COSMIN* COSMIN Consensus-based Standards for the selection of health Measurement Instruments Risk of Bias Checklist, CV Water Polo Imagery Concurrent Verbalisation (CV) Activity was developed by Watt 2003 [36] only for evaluating of criterion validity, *DPT* Degraded Pictures Test for measures object imagery, *df* degrees of freedom, *EFA* exploratory factor analysis, *HVOT* Hooper Visual Orientation Test, *ICC* interclass correlation coefficient, *JOLO* Judgement Of Line Orientation, *MAB* Multidimensional Aptitude Battery (MAB - Spatial Ability and Verbal Comprehension), *MEIQ* Mental Imagery Questionnaire, *MIQ-3* Movement Imagery Questionnaire-3, *MPFB* Minnesota Paper Board Form, *MRT* Mental Rotation of Three-dimensional Objects, *N* sample size, *NFI* normed fit index, *NNFI* non-normed fit index, *NR* not reported, *PMA* the Spatial Test of Primary Mental Abilities, *PCA *Principal Component Analysis, PFT, *RT* response time, *SEQ-S* Self-Efficacy Questionnaire—Soccer, *SFPI* Singer Fantasy Proneness Interview, *SRMR* standardised root mean square residual, *STAI-T* Trait-Angstskala des State-Trait-Angstinventars, *TLI* Tucker-Lewis index, *VKMRT* Vandenberg-Kuse=Vandenberg-Kuse Mental Rotation Test, *WAIS* Similarities Test of the conceptual similarity between the two words, *TABS* Tellegen Absorption Scale, *RSQ* Response Styles Questionnaire, *sign.* significant, *WAIS* Wechsler Adult Intelligent Scale, *WAIS-R* Wechsler Adult Intelligent Scale-Revised, *χ2* chi-square

Quality Criteria=see Table 1 Legend for explanation of quality criteria, # methods could be doubtful, students received a course credits for participation. It could be interpreted that there was a certain dependency/necessity to participate, but it was not taken into account by the COSMIN evaluation

Quality Criteria: ‘+’ = sufficient, ‘−’ insufficient, ‘?’ indeterminate. *See Table 1 and Legend for explanation of quality criteria

For criteria of EFA see de Vet et al. 2011 [52], Izquierdo et al. 2014 [61] and Watkins 2018 [62]

### Mental imagery assessments: Validity

#### Risk of bias rating

In total, 68 out of the 90 articles reported validity. A total of 18 studies [28, 42, 96, 102, 106, 111, 124, 125, 130, 141, 142, 146, 148, 150, 153, 157, 161, 166] were rated as very good or adequate and 21 studies [22, 35, 94, 98, 104, 109, 112, 115, 118, 119, 121, 127, 136, 145, 151, 152, 160, 162, 163, 165, 168] were rated as inadequate regarding their methodological quality.

#### Measurement properties

The structural, construct, content and criterion validity of most assessments were indeterminate due to lack of details reported in the studies regarding statistical methods and analysis (for more details see Tables 5 and 6). Some information about performed factor analyses such as factor loading by EFA or correlation between factors are not reported. Or the authors conducted an EFA, for which several items were loaded on more than on factor, which could indicate that these items should be deleted. However, for mostly assessments, a confirmatory factor analysis (CFA) is missing to confirm the number of extracted factors. Regarding rating of construct validity, the reviewers have formulated own hypotheses depending on comparator instruments and constructs measured. However, it was not possible for the reviewers to formulate a hypothesis in all cases as in some studies the information on the comparison instrument and the construct to be measured was insufficient. Consequently, the construct validity was rated as indeterminate. Finally, only the SIAQ revealed sufficient structural and construct validity in several studies of at least adequate methodological quality. There is moderate evidence (two studies with at least adequate methodological quality) for sufficient structural validity of the SIQ. The SIQ-C, on the other hand, has a low evidence for insufficient rating of structural validity (only two studies with doubtful methodological quality available).

### Mental imagery assessments: Reliability

#### Risk of bias rating

In total, 74 out of the 90 articles reported reliability. A total of 34 studies [29, 94–97, 102, 103, 105–107, 111, 112, 116, 118, 119, 124–126, 133, 137–140, 142, 145, 148, 150, 152–154, 157, 158, 168, 169] were rated as very good or adequate. A total of 22 studies [30, 34, 35, 41, 42, 98, 99, 101, 104, 108, 114, 115, 121, 122, 129, 132, 141, 143, 146, 156, 160, 170] were rated as inadequate regarding their methodological quality.

#### Measurement properties

The internal consistency or Cronbach’s alpha values of most assessments were reported as very high. However, for a quality rating of the internal consistency, the structural validity should also be taken into account, which finally led to an insufficient or indeterminate rating of this psychometric property. Other reasons for an insufficient rating were that in several studies the Cronbach’s alpha was calculated as multidimensional total score and not for each subscale. Only the SIAQ showed sufficient internal consistency with high evidence (multiple studies of very good methodological quality). Test-retest reliability was insufficient or indeterminate for most assessments due to an inappropriate time interval between the measurement sessions, and a poor reporting on the reliability coefficient calculation.

### Mental chronometry

Only one study [44] evaluated two assessments on mental chronometry: Time-dependent motor imagery screening test (TDMI) and Temporal Congruence Test (TCT) (Table 7). Both assessments showed sufficient test-retest reliability. No information about validity was provided. However, the methodological quality of this study was considered doubtful due to the small sample size.

**Table 7:** Mental chronometry assessments: The characteristics of the included studies - Reliability

Tool	Disciplines	Study	Country	Language	Study population				Reliability		COSMIN	Quality Criteria	Comments
					Participants	***N***	Age mean (years)	Sex	Design	Results			
Time-dependent motor imagery screening test (TDMI)	Med	Malouin et al. 2008 [44]	CA	E	Stroke^a^	20	58.3	15♀, 5♂	Test-retest	^a^Affected leg ICC=0.89–0.93 ^a^Unaffected leg ICC=0.88–0.93 ^b^Dominant leg ICC=0.88–0.89 ^b^Nondominant leg ICC=0.87–0.92	Doubtful	+	Low sample size in both groups.
					Healthy^b^	9	65.1	4♀, 5♂					
Temporal Congruence Test	Med	Malouin et al. 2008 [44]	CA	E	Stroke^a^	20	58.3	15♀, 5♂	Test-retest	^a^Affected leg ICC=0.76–0.87 ^a^Unaffected leg ICC=0.77–0.97 ^b^Dominant leg ICC=0.81–0.93 ^b^Nondominant leg ICC=0.77–0.93	Doubtful	+	Low sample size in both groups.
					Healthy^b^	9	65.1	4♀, 5♂					

Legend: The superscript numbers were used to distinguish the results per group

Disciplines in which field the tool was evaluated: Med medicine

Language of the tool: *E* English

Country abbreviations: *CA* Canada

*COSMIN* Consensus-based Standards for the selection of health Measurement Instruments Risk of Bias Checklist,* ICC* interclass correlation coefficient, *N* sample size, *NA* not applicable

Quality Criteria: ‘+’ sufficient, ‘–’ insufficient, ‘?’ indeterminate, For more information see Table 1 Legend for explanation of quality criteria

## Discussion

### Quality of studies and assessments

The aim of this systematic review was to evaluate all available assessments measuring individual imagery ability and their psychometric properties. Assessments were categorised based on their construct: motor imagery, mental imagery, and mental chronometry. A summary of the current level of evidence regarding the psychometric properties of the selected assessments is provided in the Tables 3, 4, 5, 6, and 7. All specific characteristics of the included assessments are presented in the supplementary material (Tables S1 and S3). In total, 121 articles were included reporting 155 studies evaluating psychometric properties of 65 assessments in four different disciplines. Articles reported data either about reliability or about validity. No study evaluated the responsiveness, which is defined as the ability of an instrument to detect change over time in the construct to be measured [171]. One possible reason for not reporting on responsiveness might be that the imagery ability or different imagery techniques are used for motor learning, to enhance performance, or to treat different psychological disorders. Hence, the outcome measured is not an improvement of imagery ability, and therefore, responsiveness was not evaluated.

We included in our SR only assessments that comprise items that solely focus on imagery ability. Assessments like the Sport Mental Training Questionnaire (SMTQ) [172] were excluded, as the majority of items focus on mental skills, such as performance, foundation, or interpersonal skills. Only three items of the SMTQ are focussing on imagery ability.

The methodological quality of most included studies was rated low. The reasons for this rating were for instance: a small sample size, inadequate statistical analysis or insufficient information reported. In particular, several studies calculated Cronbach’s alpha as multidimensional total score for internal consistency and not for each subscale of the assessment. The lack of reporting could lead to inaccuracy, because it is important to know the degree of inter-item correlation among the items for each subscale. Furthermore, some studies calculated the split-half reliability to report internal consistency. With this method, the correlation coefficient may not represent an accurate measure of reliability due to the fact that a single scale is being split into two scales, decreasing the reliability of the measure as a whole [173]. As proposed by COSMIN, we would recommend to calculate and report the internal consistency coefficient (usual Cronbach’s alpha for continuous scores) for each subscale separately. Specifically for structural validity, the authors did not report all details about the number of extracted factors by the EFA, the correlations among factors, the rotation methods applied and model fits from CFA (if performed). Furthermore, regarding construct validity, in some cases no information about the comparator instrument was available. Here, it was not possible to formulate a hypothesis by the reviewer to evaluate construct validity. Regarding the test-retest reliability, in several studies Person’s or Spearman’s reliability coefficient was calculated and no ICC. COSMIN recommends to calculate the ICC a two-way random effects model as the variance within individuals (e.g. systematic differences) and between time points taken into account this way. Using Pearson’s and Spearman’s correlation coefficient, systematic error is not taken into account [64]. Moreover, the time interval for test-retest reliability was sometimes not appropriate (more than 3 weeks apart), which could explain a low (< 0.70) correlation coefficient.

One possible reason for poor reporting is that the majority of the instruments were developed during the early 90s. A practical guide for conducting and reporting of such studies was published much later [52, 57, 58, 64, 174].

Further, reporting deficits in the selected studies resulted in an only substantial agreement with regard to the kappa statistic calculated between the ratings of ZS and CSA after full texts’ selection. For example, some reports did not use the usual terms for psychometric properties when describing the study aim [129, 167]. This led to a confusion among the authors (ZS and CSA) in their attempt to determine which psychometric properties were evaluated.

The psychometric properties for most of the assessments regarding construct validity (e.g. correlation with other measures) and criterion validity were rated as indeterminate or insufficient. These findings corresponded to previous studies [39, 48]. A possible explanation could be that most of these questionnaires are self-reports and the individuals should express the ease or vividness of imagery in relation to the Likert scale. There are no references or standards against which reports of imagery experience can be validated. This is not trivial, considering that the idea about what a vivid image is can vary greatly from person to person. Moreover, the objective and subjective assessments showed low correlation suggesting that these two types of imagery (object and spatial) are not related to each other. Previous studies reported the same findings [22, 34, 35]. Structural validity by most assessments was also considered as indeterminate or insufficient. For example, in several studies, when evaluating Betts Questionnaire, the GTVIC, or the CAIS, only the EFA was conducted and reported. Depending on the method of analysis used in different studies, the number of extracted factors varied greatly. No study conducted a CFA to confirm the number of factors identified. Further, particularly the evaluation of the Betts Questionnaire by various studies [102, 104, 161] showed that some items seem to be unstable on the kinaesthetic and the visual scale and should be removed. This is very interesting, as most of the other assessments for measuring individual differences in imagery were developed based on the Betts Questionnaire as a pioneer assessment, whose structural validity may be considered as indeterminate.

Almost all studies, when reporting psychometric properties of the comparator instrument or the ‘gold standard’ instrument, only reported about reliability (e.g. internal consistency), which is in most cases very high. Such assessments often lacked structural or criterion validity but authors did not critically discuss that. In addition, most studies were only conducted with students aged 12–28 years, who received a course credit for study participation.

The best-evaluated assessments with sufficient psychometric properties were the MIQ, MIQ-R, MIQ-3 and VMIQ-2 for evaluation of motor imagery ability. They are mostly applied in the field of sport. All assessments are self-reports, very easy to use and evaluate vividness in two modalities: visual and kinaesthetic. Moreover, the MIQ-3 and VMIQ-2 evaluate also the perspective used during imagination: external or internal. The MIQ-3 is translated into several languages, which enables a wide use. The SIAQ as mental imagery assessment in sport showed sufficient psychometric properties, but the SIAQ is not able to distinguish between ease of imaging and vividness. The VVIQ was evaluated only with psychology students, and only internal consistency was sufficient. In the field of medicine, the KVIQ is the most evaluated assessment, focusing on vividness in two modalities: visual and kinaesthetic. The original version KVIQ-20 is translated into several languages, but due to the number of items, applying the KVIQ-20 can be quite time-consuming. Structural validity is particularly critical and further studies with large sample sizes and the use of a CFA are needed. Although all assessments described above are self-report, easy to use and cost-effective, a general limitation of these assessments is that they do not allow to control for imagery ability before or during an experiment.

Our results demonstrate that there are a number of published instruments for measuring the imagery ability in different disciplines. We categorised all assessments based on their construct and a clear differentiation between the terms ‘motor imagery’ and ‘mental imagery’. These terms are often confused in the literature.

### Limitations regarding the COSMIN recommendations

As proposed by COSMIN, sample sizes are not taken into account when assessing study quality in terms of reliability. It is recommended, however, that sample size should be taken into account at a later step of the review process when the results of all available studies can be summarised (e.g. as imprecision, which refers to the total sample size). Hence, the pooled evidence from many small studies together can provide strong evidence for good reliability [64]. However, in our review, it was not possible to pool or qualitatively summarise the results from all small studies with *n* = ≤30 due to their different subgroups of patients, different language versions and inconsistency of results. Therefore, we downgraded every study with a small sample size for imprecision as having a risk of bias. We used the ‘other flaws’ option to take this into account. For other psychometric properties like content validity or structural validity, there are standards concerning the sample size. However, some measures were developed and evaluated only for a specific population (e.g. patients) [68, 69]. Therefore, a large sample size is often not feasible, but robust data can be expected due to homogeneity. In cases where we estimated the sample size to be low, most of these studies were of inadequate methodological quality [67–69]. On the other hand, several studies with a large sample size (e.g. students), when the target population for a specific measure was not clearly described, were rated as ‘adequate’ or ‘very good’ [141, 142].

In our opinion, the studies with healthy individuals (students, athletes, etc.) or with patients should be more differentiated during evaluation following the COSMIN guideline.

### Systematic review limitations and strengths

A limitation of our systematic review is that we did not emphasize on content validity of the evaluated assessments. We rated content validity only in case the authors did specify this as one of their study aims and included a sufficient description of the performed procedures. However, there were some questionnaire development studies, which could be considered assessing content validity. Nevertheless, most of the questionnaire development studies lacked important information about whether the target population was asked about relevance, comprehensiveness and comprehensibility of the questionnaire under development. The authors focused on reporting the validation steps. Therefore, we could not conclude, if the evaluation of content validity was not performed or not reported. Furthermore, we used the COSMIN evaluation tool, a widely accepted and valid tool for rating the methodological quality of studies. However, the COSMIN evaluation of methodology is strictly based on information published in the studies. As most identified articles were published more than 20 years ago, authors could not be contacted to request additional details. Therefore, some ratings as ‘doubtful’ could have been inequitable. In addition, our search was limited to English or German, so relevant articles may have been excluded. We applied the filter published by Terwee et al. [54] and adapted it for each database. However, we identified many articles by screening the references. The main reason why our filter did not find such articles is that the measurement properties are sometimes poorly reported in the abstract and some authors did not use any commonly used term for measurement properties in the title or abstract of their article. There is a large variation concerning terminology for measurement properties. For example, for reliability, many synonyms can be found in the literature (e.g. reproducibility, repeatability, precision, variability, consistency, dependability, stability, agreement, and measurement error) [54]. However, the composition of the search strategy and the search itself were conducted by a professional research librarian from the University of Zurich in accordance with the review protocol providing a comprehensive search and detailed knowledge of different databases in all four disciplines. Therefore, the search was easily reproduced and verified by ZS resulting in the same number of identified records. Moreover, all references were selected by two authors (ZS and CSA) and several reviewers extracted and double-checked all the data from the included articles, which limited the risk of errors in the extraction process.

## Conclusion

Over the last century, various assessments were developed to evaluate an individual’s imagery ability within different dimensions or modalities of imagery: vividness or image clarity, controllability, ease and accuracy of how an image can be mentally manipulated, perspective used, frequency of use of imagery and imagery preferences (verbal or visual style). However, the validity of many assessments is insufficient or indeterminate. Although reliability, in particular internal consistency, of most assessments was reported as high (Cronbach’s alpha > 0.70), due to insufficient or indeterminate structural validity this property of imagery assessment should also be regarded very critically. Furthermore, the COSMIN recommendations classified most studies as inadequate or doubtful due to small sample sizes, inadequate statistical analyses used, or an insufficient reporting. Most studies were conducted with young students and further studies are needed in other fields and wider age ranges.

Despite the limitations described, the present systematic review enables clinicians, coaches, teachers, and researchers to select a suitable imagery ability assessment for their settings and goals based on information provided regarding the assessment’s focus and quality.

## Supplementary Information

**Additional file 1.** Example search strategy for web of science.

**Additional file 2.** COSMIN Risk of Bias checklist.

**Additional file 3: Table 1S.** Characteristics of the Included Measurement Tools for Motor Imagery.

**Additional file 4: Table 2S.** Motor imagery: Summary of Findings using modified GRADE.

**Additional file 5: Table 3S.** Characteristics of the Included Measurement Tools for Mental Imagery.

**Additional file 6: Table 4S.** Mental imagery Assessments: Summary of Findings using modified GRADE.

## Data Availability

For the present systematic literature review, we used data from already published articles. All data from our further analysis can be found within the report.

## Abbreviations

CFA: Confirmatory factor analysis
COSMIN: COnsensus-based Standards for the selection of health Measurement Instruments
EFA: Explorative factor analysis
GRADE: Grading of Recommendations Assessment, Development, and Evaluation
PROM: Patient-Reported Outcome Measures
SoF: Summary of Findings
