# Supplementary material for: Imagery ability assessments: a cross-disciplinary systematic review and quality evaluation of psychometric properties
Source: BMC Med. 2022 May 2;20:166. doi: 10.1186/s12916-022-02295-3 (PMC9059408; doi:10.1186/s12916-022-02295-3)
Supplement: Supplementary file 1 — Additional file 1. Example search strategy for web of science. [file 12916_2022_2295_MOESM1_ESM.pdf]

Search History **Web of Science Core Collection**

Set	Search Terms	Results
# 1	((TS=("motor imag*" OR "mental imag*" OR "movement imag*" OR "mental rehearsal*" OR "mental pract*" OR "mental training"))) *AND* LANGUAGE: (English OR German)	8,964
# 2	((TS=(instrument* OR measure* OR questionnaire* OR scale* OR assess* OR test*))) *AND* LANGUAGE: (English OR German)	11,763,813
# 3	((TS=(psychometr* OR clinimetr* OR clinometr* OR "outcome assessment" OR "outcome measure*" OR "observer variation" OR reproducib* OR reliab* OR unreliab* OR valid* OR coefficient OR homogeneity OR homogeneous OR "internal consistency" OR (cronbach* AND (alpha OR alphas)) OR (item AND (correlation* OR selection* OR reduction*)) OR agreement OR precision OR imprecision OR "precise values" OR "test-retest" OR (test AND retest) OR (reliab* AND (test OR retest)) OR stability OR interrater OR inter-rater OR intrarater OR intrarater OR intertester OR inter-tester OR intratester OR intra-tester OR interobserver OR interobserver OR intraobserver OR intra-observer OR intertechnician OR inter-technician OR intratechnician OR intra-technician OR interexaminer OR inter-examiner OR intraexaminer OR intra-examiner OR interassay OR inter-assay OR intraassay OR intraassay OR interindividual OR inter-individual OR intraindividual OR intra-individual OR interparticipant OR inter-participant OR intraparticipant OR intra-participant OR kappa OR kappa's OR kappas OR repeatab* OR ((replicab* OR repeated) AND (measure OR measures OR findings OR result OR results OR test OR tests)) OR generaliza* OR generalisa* OR concordance OR (intraclass AND correlation*) OR discriminative OR "knowngroup" OR "factor analysis" OR "factor analyses" OR dimension* OR subscale* OR (multitrait AND scaling AND (analysis OR analyses)) OR "item discriminant" OR "interscale correlation*" OR error OR errors OR "individual variability" OR (variability AND (analysis OR values)) OR (uncertainty AND (measurement OR measuring)) OR "standard error of measurement" OR sensitiv* OR responsive* OR ((minimal OR minimally OR clinical OR clinically) AND (important OR significant OR detectable) AND (change OR difference)) OR (small* AND (real OR detectable) AND (change OR difference)) OR "meaningful change" OR "ceiling effect" OR "floor effect" OR "Item response model" OR IRT OR Rasch OR "Differential item unctioning" OR DIF OR "computer adaptive testing" OR "item bank" OR "cross-cultural equivalence"))) AND LANGUAGE: (English OR German)	9,659,388
# 4	#3 AND #2 AND #1	1,408
