# Supplementary material for: Imagery ability assessments: a cross-disciplinary systematic review and quality evaluation of psychometric properties
Source: BMC Med. 2022 May 2;20:166. doi: 10.1186/s12916-022-02295-3 (PMC9059408; doi:10.1186/s12916-022-02295-3)
Supplement: Supplementary file 2 — Additional file 2. COSMIN Risk of Bias checklist. [file 12916_2022_2295_MOESM2_ESM.pdf]

COSMIN Risk of Bias checklist

Date: July, 2018

Contact
L.B. Mokkink, PhD
VU University Medical Center
Department of Epidemiology and Biostatistics
Amsterdam Public Health research institute
P.O. box 7057
1007 MB Amsterdam
The Netherlands
Website: www.cosmin.nl
E-mail: w.mokkink@vumc.nl

COSMIN

How to site the COSMIN Risk of Bias Checklist

Please refer to the following studies when using the COSMIN Risk of Bias Checklist:

Mokkink, L.B., De Vet, H.C.W., Prinsen, C.A.C, Patrick, D.L., Alonso, J., Bouter, L.M., et al. COSMIN Risk of Bias checklist for systematic reviews of Patient-Reported Outcome Measures. Accepted for publication in Quality of Life Research.

Prinsen, C. A., Mokkink, L. B., Bouter, L. M., Alonso, J., Patrick, D. L., Vet, H. C., et al. COSMIN guideline for systematic reviews of Patient-Reported Outcome Measures. Submitted.

Terwee, C. B., Prinsen, C. A., Chiarotto, A., Vet, H. C., Westerman, M. J., Patrick, D. L., et al. COSMIN methodology for evaluating the content validity of Patient-Reported Outcome Measures: a Delphi study. Submitted.

For details on how to use the COSMIN risk of Bias checklist see 'COSMIN methodology for systematic reviews of Patient-Reported Outcome Measures (PROMs) – user manual' and 'COSMIN methodology for assessing the content validity of Patient-Reported Outcome Measures (PROMs) - user manual' available from our website www.cosmin.nl.

Abbreviations used:

CTT – classical test theory

DIF – differential item functioning

IRT – Item response theory

MGCFA – multi-group confirmatory factor analysis

MI – measurement invariance

NA – not applicable

PROM – patient-reported outcome measure

1PL model – 1 parameter IRT model

2PL model – 2 parameter IRT model

Instructions

Tick the boxes that need to be completed for the article

	**COSMIN Risk of Bias checklist**
	Box 1. PROM development
	Box 2. Content validity
	Box 3. Structural validity
	Box 4. Internal consistency
	Box 5. Cross-cultural validity\Measurement invariance
	Box 6. Reliability
	Box 7. Measurement error
	Box 8. Criterion validity
	Box 9. Hypotheses testing for construct validity
	Box 10. Responsiveness

To assess the methodological quality of each study, i.e. assessing the risk of bias of the result of a study, the corresponding COSMIN Risk of Bias box should be completed. To determine the overall quality of a study the lowest rating of any standard in the box is taken (i.e. "the worst score counts" principle). For example, if for a reliability study one item in a box is rated as 'inadequate', the overall methodological quality of that reliability study is rated as 'inadequate'. The response option 'NA' (not applicable) is at issue for some standards. For example, when a study on structural validity is based on CTT, the standard on IRT is not applicable and this standard should not be considered in the "worst score counts"-rating for that specific study. For standards where this option is not at issue, these cells are grey and shouldn't be used.

Box 1. PROM development

1a. PROM design

General design requirements		very good	adequate	doubtful	inadequate	NA
1	Is a clear description provided of the construct to be measured?	Construct clearly described			Construct not clearly described	
2	Is the origin of the construct clear: was a theory, conceptual framework or disease model used or clear rationale provided to define the construct to be measured?	Origin of the construct clear		Origin of the construct not clear		
3	Is a clear description provided of the target population for which the PROM was developed?	Target population clearly described			Target population not clearly described	
4	Is a clear description provided of the context of use	Context of use clearly described		Context of use not clearly described		
5	Was the PROM development study performed in a sample representing the target population for which the PROM was developed?	Study performed in a sample representing the target population	Assumable that the study was performed in a sample representing the target population, but not clearly described	Doubtful whether the study was performed in a sample representing the target population	Study not performed in a sample representing the target population **(SKIP items 6-12)**	

Concept elicitation (relevance and comprehensiveness)		very good	adequate	doubtful	inadequate	NA
6	Was an appropriate qualitative data collection method used to identify relevant items for a new PROM?	Widely recognized or well justified qualitative method used, suitable for the construct and study population	Assumable that the qualitative method was appropriate and suitable for the construct and study population, but not clearly described	Only quantitative (survey) method(s) used or doubtful whether the method was suitable for the construct and study population	Method used not appropriate or not suitable for the construct or study population	
7	Were skilled group moderators/interviewers used?	Skilled group moderators/ interviewers used	Group moderators /interviewers had limited experience or were trained specifically for the study	Not clear if group moderators /interviewers were trained or group moderators /interviewers not trained and no experience		Not applicable
8	Were the group meetings or interviews based on an appropriate topic or interview guide?	Appropriate topic or interview guide	Assumable that the topic or interview guide was appropriate, but not clearly described	Not clear if a topic guide was used or doubtful if topic or interview guide was appropriate or no guide		Not applicable

9	Were the group meetings or interviews recorded and transcribed verbatim?	All group meetings or interviews were recorded and transcribed verbatim	Assumable that all group meetings or interviews were recorded and transcribed verbatim, but not clearly described	Not clear if all group meetings of interviews were recorded and transcribed verbatim or recordings not transcribed verbatim or only notes were made during the group meetings/ interviews	No recording and no notes	Not applicable
10	Was an appropriate approach used to analyse the data?	A widely recognized or well justified approach was used	Assumable that the approach was appropriate, but not clearly described	Not clear what approach was used or doubtful whether the approach was appropriate	Approach not appropriate	
11	Was at least part of the data coded independently?	At least 50% of the data was coded by at least two researchers independently	11-49% of the data was coded by at least two researchers independently	Doubtful if two researchers were involved in the coding or only 1-10% of the data was coded by at least two researchers independently	Only one researcher was involved in coding or no coding	Not applicable
12	Was data collection continued until saturation was reached?	Evidence provided that saturation was reached	Assumable that saturation was reached	Doubtful whether saturation was reached	Evidence suggests that saturation was not reached	Not applicable
13	For quantitative studies (surveys): was the sample size appropriate?	≥100	50-99	30-49	<30	Not applicable

1b. Cognitive interview study or other pilot test	very good	adequate	doubtful	inadequate	NA
14 Was a cognitive interview study or other pilot test conducted?	YES			**NO (SKIP items 15-35)**	
General design requirements					
15 Was the cognitive interview study or other pilot test performed in a sample representing the target population?	Study performed in a sample representing the target population	Assumable that the study was performed in a sample representing the target population, but not clearly described	Doubtful whether the study was performed in a sample representing the target population	Study not performed in a sample representing the target population	
Comprehensibility					
16 Were patients asked about the comprehensibility of the PROM?	YES		Not clear (SKIP standards 17-25)	No **(SKIP standards 17-25)**	
17 Were all items tested in their final form?	All items were tested in their final form	Assumable that all items were tested in their final form, but not clearly described	Not clear if all items were tested in their final form	Items were not tested in their final form or items were not re-tested after substantial adjustments	

18	Was an appropriate qualitative method used to assess the <u>comprehensibility</u> of the PROM instructions, items, response options, and recall period?	Widely recognized or well justified qualitative method used	Assumable that the method was appropriate but not clearly described	Only quantitative (survey) method(s) used or doubtful whether the method was appropriate or not clear if patients were asked about the comprehensibility of the items, response options or recall period or patients not asked about the comprehensibility of the PROM instructions or the recall period	Method used not appropriate or patients not asked about the comprehensibility of the items or the response options	
19	Was each item tested in an appropriate number of patients?<br>For qualitative studies<br>For quantitative (survey) studies	 ≥7<br>≥50	 4-6<br>≥30	 <4 or not clear<br><30 or not clear		
20	Were skilled interviewers used?	Skilled group moderators/ interviewers used	Group moderators /interviewers had limited experience or were trained specifically for the study	Not clear if group moderators /interviewers were trained or group moderators /interviewers not trained and no experience		Not applicable
21	Were the interviews based on an appropriate interview guide?	Appropriate topic or interview guide	Assumable that the topic or interview guide was appropriate, but not clearly described	Not clear if a topic guide was used or doubtful if topic or interview guide was appropriate or no guide		Not applicable

22	Were the interviews recorded and transcribed verbatim?	All group meetings or interviews were recorded and transcribed verbatim	Assumable that all group meetings or interviews were recorded and transcribed verbatim, but not clearly described	Not clear if all group meetings or interviews were recorded and transcribed verbatim or recordings not transcribed verbatim or only notes were made during the group meetings/ interviews	No recording and no notes	Not applicable
23	Was an appropriate approach used to analyse the data?	A widely recognized or well justified approach was used	Assumable that the approach was appropriate, but not clearly described	Not clear what approach was used or doubtful whether the approach was appropriate	Approach not appropriate	
24	Were at least two researchers involved in the analysis?	At least two researchers involved in the analysis	Assumable that at least two researchers were involved in the analysis, but not clearly described	Not clear if two researchers were included in the analysis or only one researcher involved in the analysis		
25	Were problems regarding the comprehensibility of the PROM instructions, items, response options, and recall period appropriately addressed by adapting the PROM?	No problems found or problems appropriately addressed and PROM was adapted and re-tested if necessary	Assumable that there were no problems or that problems were appropriately addressed, but not clearly described	Not clear if there were problems or doubtful if problems were appropriately addressed	Problems not appropriately addressed or PROM was adapted but items were not re-tested after substantial adjustments.	Not applicable

Comprehensiveness		very good	adequate	doubtful	inadequate	NA
26	Were patients asked about the comprehensiveness of the PROM?	YES		NO or not clear **(SKIP items 27-35)**		
27	Was the final set of items tested?	The final set of items was tested	Assumable that the final set of items was tested, but not clearly described	Not clear if the final set of items was tested or not the final set of items was tested or the set of items was not re-tested after items were removed or added		
28	Was an appropriate method used for assessing the comprehensiveness of the PROM?	Widely recognized or well justified method used	Assumable that the method was appropriate but not clearly described or only quantitative (survey) method(s) used	Doubtful whether the method was appropriate or method used not appropriate		
29	Was each item tested in an appropriate number of patients?					
	For qualitative studies	≥7	4-6	<4 or not clear		
	For quantitative (survey) studies	≥50	≥30	<30 or not clear		
30	Were skilled interviewers used?	Skilled interviewers used	Interviewers had limited experience or were trained specifically for the study	Not clear if interviewers were trained or interviewers not trained and no experience		Not applicable

31	Were the interviews based on an appropriate interview guide?	Appropriate topic or interview guide	Assumable that the topic or interview guide was appropriate, but not clearly described	Not clear if a topic guide was used or doubtful if topic or interview guide was appropriate or no guide		Not applicable
32	Were the interviews recorded and transcribed verbatim?	All group meetings or interviews were recorded and transcribed verbatim	Assumable that all group meetings or interviews were recorded and transcribed verbatim, but not clearly described	Not clear if all group meetings or interviews were recorded and transcribed verbatim or recordings not transcribed verbatim or only notes were made during the group meetings/ interviews or no recording and no notes		Not applicable
33	Was an appropriate approach used to analyse the data?	A widely recognized or well justified approach was used	Assumable that the approach was appropriate, but not clearly described	Not clear what approach was used or doubtful whether the approach was appropriate or approach not appropriate		
34	Were at least two researchers involved in the analysis?	At least two researchers involved in the analysis	Assumable that at least two researchers were involved in the analysis, but not clearly described	Not clear if two researchers were included in the analysis or only one researcher involved in the analysis		

35	Were problems regarding the comprehensiveness of the PROM appropriately addressed by adapting the PROM?	No problems found or problems appropriately addressed and PROM was adapted and re-tested if necessary	Assumable that there were no problems or that problems were appropriately addressed, but not clearly described	Not clear if there were problems or doubtful if problems were appropriately addressed or PROM was adapted but items were not re-tested after substantial adjustments	Problems not appropriately addressed	Not applicable

Box 2. Content validity

2a. Asking patients about relevance

Design requirements	**very good**	**adequate**	**doubtful**	**inadequate**	**NA**
1 Was an appropriate method used to ask patients whether each item is relevant for their experience with the condition?	Widely recognized or well justified method used	Only quantitative (survey) method(s) used or assumable that the method was appropriate but not clearly described	Not clear if patients were asked whether each item is relevant or doubtful whether the method was appropriate	Method used not appropriate or patients not asked about the relevance of all items	
2 Was each item tested in an appropriate number of patients?					
For qualitative studies	≥7	4-6	<4 or not clear		
For quantitative (survey) studies	≥50	≥30	<30 or not clear		
3 Were skilled group moderators/interviewers used?	Skilled group moderators/ interviewers used	Group moderators /interviewers had limited experience or were trained specifically for the study	Not clear if group moderators /interviewers were trained or group moderators /interviewers not trained and no experience		Not applicable
4 Were the group meetings or interviews based on an appropriate topic or interview guide?	Appropriate topic or interview guide	Assumable that the topic or interview guide was appropriate, but not clearly described	Not clear if a topic guide was used or doubtful if topic or interview guide was appropriate or no guide		Not applicable

5	Were the group meetings or interviews recorded and transcribed verbatim?	All group meetings or interviews were recorded and transcribed verbatim	Assumable that all group meetings or interviews were recorded and transcribed verbatim, but not clearly described	Not clear if all group meetings or interviews were recorded and transcribed verbatim or recordings not transcribed verbatim or only notes were made during the group meetings/ interviews	No recording and no notes	Not applicable
Analyses						
6	Was an appropriate approach used to analyse the data?	A widely recognized or well justified approach was used	Assumable that the approach was appropriate, but not clearly described	Not clear what approach was used or doubtful whether the approach was appropriate	Approach not appropriate	
7	Were at least two researchers involved in the analysis?	At least two researchers involved in the analysis	Assumable that at least two researchers were involved in the analysis, but not clearly described	Not clear if two researchers were included in the analysis or only one researcher involved in the analysis		

**2b Asking patients about comprehensiveness**						
Design requirements		**very good**	**adequate**	**doubtful**	**inadequate**	**NA**
8	Was an appropriate method used for assessing the comprehensiveness of the PROM?	Widely recognized or well justified method used	Only quantitative (survey) method(s) used or assumable that the method was appropriate but not clearly described	Doubtful whether the method was appropriate	Method used not appropriate	
9	Was each item tested in an appropriate number of patients?<br>For qualitative studies<br>For quantitative (survey) studies	 ≥7<br>≥50	 4-6<br>≥30	 <4 or not clear<br><30 or not clear		
10	Were skilled group moderators/interviewers used?	Skilled group moderators/ interviewers used	Group moderators /interviewers had limited experience or were trained specifically for the study	Not clear if group moderators /interviewers were trained or group moderators /interviewers not trained and no experience		Not applicable
11	Were the group meetings or interviews based on an appropriate topic or interview guide?	Appropriate topic or interview guide	Assumable that the topic or interview guide was appropriate, but not clearly described	Not clear if a topic guide was used or doubtful if topic or interview guide was appropriate or no guide		Not applicable

12	Were the group meetings or interviews recorded and transcribed verbatim?	All group meetings or interviews were recorded and transcribed verbatim	Assumable that all group meetings or interviews were recorded and transcribed verbatim, but not clearly described	Not clear if all group meetings or interviews were recorded and transcribed verbatim or recordings not transcribed verbatim or only notes were made during the group meetings/ interviews	No recording and no notes	Not applicable
Analyses						
13	Was an appropriate approach used to analyse the data?	A widely recognized or well justified approach was used	Assumable that the approach was appropriate, but not clearly described	Not clear what approach was used or doubtful whether the approach was appropriate	Approach not appropriate	
14	Were at least two researchers involved in the analysis?	At least two researchers involved in the analysis	Assumable that at least two researchers were involved in the analysis, but not clearly described	Not clear if two researchers were included in the analysis or only one researcher involved in the analysis		

2c Asking patients about comprehensibility

Design requirements		very good	adequate	doubtful	inadequate	NA
15	Was an appropriate qualitative method used for assessing the comprehensibility of the PROM instructions, items, response options, and recall period?	Widely recognized or well justified qualitative method used	Assumable that the method was appropriate but not clearly described	Only quantitative (survey) method(s) used or doubtful whether the method was appropriate or not clear if patients were asked about the comprehensibility of the items, response options or recall period or patients not asked about the comprehensibility of the PROM instructions	Method used not appropriate or patients not asked about the comprehensibility of the items, response options, or recall period	
16	Was each item tested in an appropriate number of patients?					
	For qualitative studies	≥7	4-6	<4 or not clear		
	For quantitative (survey) studies	≥50	≥30	<30 or not clear		
17	Were skilled group moderators/interviewers used?	Skilled group moderators/ interviewers used	Group moderators /interviewers had limited experience or were trained specifically for the study	Not clear if group moderators /interviewers were trained or group moderators /interviewers not trained and no experience		

18	Were the group meetings or interviews based on an appropriate topic or interview guide?	Appropriate topic or interview guide	Assumable that the topic or interview guide was appropriate, but not clearly described	Not clear if a topic guide was used or doubtful if topic or interview guide was appropriate or no guide		Not applicable
19	Were the group meetings or interviews recorded and transcribed verbatim?	All group meetings or interviews were recorded and transcribed verbatim	Assumable that all group meetings or interviews were recorded and transcribed verbatim, but not clearly described	Not clear if all group meetings or interviews were recorded and transcribed verbatim or recordings not transcribed verbatim or only notes were made during the group meetings/ interviews	No recording and no notes	Not applicable
Analyses						
20	Was an appropriate approach used to analyse the data?	A widely recognized or well justified approach was used	Assumable that the approach was appropriate, but not clearly described	Not clear what approach was used or doubtful whether the approach was appropriate	Approach not appropriate	
21	Were at least two researchers involved in the analysis?	At least two researchers involved in the analysis	Assumable that at least two researchers were involved in the analysis, but not clearly described	Not clear if two researchers were included in the analysis or only one researcher involved in the analysis		

2d. Asking professionals about relevance

Design requirements		very good	adequate	doubtful	inadequate	NA
22	Was an appropriate method used to ask professionals whether each item is relevant for the construct of interest?	Widely recognized or well justified method used	Only quantitative (survey) method(s) used or assumable that the method was appropriate but not clearly described	Not clear if professionals were asked whether each item is relevant or doubtful whether the method was appropriate	Method used not appropriate or professionals not asked about the relevance of all items	
23	Were professionals from all relevant disciplines included?	Professionals from all required disciplines were included	Assumable that professionals from all required disciplines were included, but not clearly described	Doubtful whether professionals from all required disciplines were included or relevant professionals were not included		
24	Was each item tested in an appropriate number of professionals?					
	For qualitative studies	≥7	4-6	<4 or not clear		
	For quantitative (survey) studies	≥50	≥30	<30 or not clear		
Analyses						
25	Was an appropriate approach used to analyse the data?	A widely recognized or well justified approach was used	Assumable that the approach was appropriate, but not clearly described	Not clear what approach was used or doubtful whether the approach was appropriate	Approach not appropriate	

26	Were at least two researchers involved in the analysis?	At least two researchers involved in the analysis	Assumable that at least two researchers were involved in the analysis, but not clearly described	Not clear if two researchers were included in the analysis or only one researcher involved in the analysis	

2e. Asking professionals about comprehensiveness

Design requirement		very good	adequate	doubtful	inadequate	NA
27	Was an appropriate method used for assessing the <u>comprehensiveness</u> of the PROM?	Widely recognized or well justified method used	Only quantitative (survey) method(s) used or assumable that the method was appropriate but not clearly described	Doubtful whether the method was appropriate	Method used not appropriate	
28	Were professionals from all relevant disciplines included?	Professionals from all required disciplines were included	Assumable that professionals from all required disciplines were included, but not clearly described	Doubtful whether professionals from all required disciplines were included or relevant professionals were not included		
29	Was each item tested in an appropriate number of professionals?<br>For qualitative studies<br>For quantitative (survey) studies	 ≥7<br>≥50	 4-6<br>≥30	 <4 or not clear<br><30 or not clear		
Analyses						
30	Was an appropriate approach used to analyse the data?	A widely recognized or well justified approach was used	Assumable that the approach was appropriate, but not clearly described	Not clear what approach was used or doubtful whether the approach was appropriate	Approach not appropriate	

31	Were at least two researchers involved in the analysis?	At least two researchers involved in the analysis	Assumable that at least two researchers were involved in the analysis, but not clearly described	Not clear if two researchers were included in the analysis or only one researcher involved in the analysis	

Box 3. Structural validity

Does the scale consist of effect indicators, i.e. is it based on a reflective model? [1] yes / no

Does the study concern unidimensionality or structural validity? [2] unidimensionality / structural validity

Statistical methods	very good	adequate	doubtful	inadequate	NA
1 For CTT: Was exploratory or confirmatory factor analysis performed?	Confirmatory factor analysis performed	Exploratory factor analysis performed		No exploratory or confirmatory factor analysis performed	Not applicable
2 For IRT/Rasch: does the chosen model fit to the research question?	Chosen model fits well to the research question	Assumable that the chosen model fits well to the research question	Doubtful if the chosen model fits well to the research question	Chosen model does not fit to the research question	Not applicable
3 Was the sample size included in the analysis adequate?	FA: 7 times the number of items and ≥100	FA: at least 5 times the number of items and ≥100; OR at least 6 times number of items but <100	FA: 5 times the number of items but <100	FA: < 5 times the number of items	
	Rasch/1PL models: ≥ 200 subjects	Rasch/1PL models: 100-199 subjects	Rasch/1PL models: 50-99 subjects	Rasch/1PL models: < 50 subjects	
	2PL parametric IRT models OR Mokken scale analysis: ≥ 1000 subjects	2PL parametric IRT models OR Mokken scale analysis: 500-999 subjects	2PL parametric IRT models OR Mokken scale analysis: 250-499 subjects	2PL parametric IRT models OR Mokken scale analysis: < 250 subjects	

Other						
4	Were there any other important flaws in the design or statistical methods of the study?	No other important methodological flaws		Other minor methodological flaws (e.g. rotation method not described)	Other important methodological flaws (e.g. inappropriate rotation method)	

[1] If the scale is not based on a reflective model, unidimensionality or structural validity is not relevant.
[2] In a systematic review, it is helpful to make a distinction between studies where factor analysis is performed on each (sub)scale separately to evaluate whether the (sub)scales are unidimensional (unidimensionality studies) and studies where factor analysis is performed on all items of an instrument to evaluate the (expected) number of subscales in the instrument and the clustering of items within subscales (structural validity studies).

Box 4. Internal consistency

Does the scale consist of effect indicators, i.e. is it based on a reflective model? [1] yes / no

Design requirements	very good	adequate	doubtful	inadequate	NA
1 Was an internal consistency statistic calculated for each unidimensional scale or subscale separately?	Internal consistency statistic calculated for each unidimensional scale or subscale		Unclear whether scale or sub scale is unidimensional	Internal consistency statistic NOT calculated for each unidimensional scale or sub scale	
Statistical methods					
2 For continuous scores: Was Cronbach's alpha or omega calculated?	Cronbach's alpha, or Omega calculated		Only item-total correlations calculated	No Cronbach's alpha and no item-total correlations calculated	Not applicable
3 For dichotomous scores: Was Cronbach's alpha or KR-20 calculated?	Cronbach's alpha or KR-20 calculated		Only item-total correlations calculated	No Cronbach's alpha or KR-20 and no item-total correlations calculated	Not applicable
4 For IRT-based scores: Was standard error of the theta (SE (θ)) or reliability coefficient of estimated latent trait value (index of (subject or item) separation) calculated?	SE(θ) or reliability coefficient calculated			SE(θ) or reliability coefficient NOT calculated	Not applicable
Other					
5 Were there any other important flaws in the design or statistical methods of the study?	No other important methodological flaws		Other minor methodological flaws	Other important methodological flaws	

[1] If the scale is not based on a reflective model, internal consistency is not relevant

Box 5. Cross-cultural validity\Measurement invariance					
Design requirements	**very good**	**adequate**	**doubtful**	**inadequate**	**NA**
1 Were the samples similar for relevant characteristics except for the group variable?	Evidence provided that samples were similar for relevant characteristics except group variable	Stated (but no evidence provided) that samples were similar for relevant characteristics except group variable	Unclear whether samples were similar for relevant characteristics except group variable	Samples were NOT similar for relevant characteristics except group variable	
Statistical methods					
2 Was an appropriate approach used to analyse the data?	A widely recognized or well justified approach was used	Assumable that the approach was appropriate, but not clearly described	Not clear what approach was used or doubtful whether the approach was appropriate	Approach not appropriate	Not applicable
3 Was the sample size included in the analysis adequate?	Regression analyses or IRT/Rasch based analyses: 200 subjects per group	150 subjects per group	100 subjects per group	< 100 subjects per group	
	MGCFA*: 7 times the number of items and ≥100	5 times the number of items and ≥100; OR 5-7 times the number of items but <100	5 times the number of items but <100	<5 times the number of items	
Other					
4 Were there any other important flaws in the design or statistical methods of the study?	No other important methodological flaws		Other minor methodological flaws	Other important methodological flaws	

**MGCFA: multi-group confirmatory factor analyses*

Box 6. Reliability

Design requirements		very good	adequate	doubtful	inadequate	NA
1	Were patients stable in the interim period on the construct to be measured?	Evidence provided that patients were stable	Assumable that patients were stable	Unclear if patients were stable	Patients were NOT stable	
2	Was the time interval appropriate?	Time interval appropriate		Doubtful whether time interval was appropriate or time interval was not stated	Time interval NOT appropriate	
3	Were the test conditions similar for the measurements? e.g. type of administration, environment, instructions	Test conditions were similar (evidence provided)	Assumable that test conditions were similar	Unclear if test conditions were similar	Test conditions were NOT similar	
Statistical methods						
4	For continuous scores: Was an intraclass correlation coefficient (ICC) calculated?	ICC calculated and model or formula of the ICC is described	ICC calculated but model or formula of the ICC not described or not optimal. Pearson or Spearman correlation coefficient calculated with evidence provided that no systematic change has occurred	Pearson or Spearman correlation coefficient calculated WITHOUT evidence provided that no systematic change has occurred or WITH evidence that systematic change has occurred	No ICC or Pearson or Spearman correlations calculated	Not applicable
5	For dichotomous/nominal/ordinal scores: Was kappa calculated?	Kappa calculated			No kappa calculated	Not applicable

6	For ordinal scores: Was a weighted kappa calculated?	Weighted Kappa calculated		Unweighted Kappa calculated or not described		Not applicable
7	For ordinal scores: Was the weighting scheme described? e.g. linear, quadratic	Weighting scheme described	Weighting scheme NOT described			Not applicable
Other						
8	Were there any other important flaws in the design or statistical methods of the study?	No other important methodological flaws		Other minor methodological flaws	Other important methodological flaws	

Box 7. Measurement error

Design requirements		very good	adequate	doubtful	Inadequate	NA
1	Were patients stable in the interim period on the construct to be measured?	Patients were stable (evidence provided)	Assumable that patients were stable	Unclear if patients were stable	Patients were NOT stable	
2	Was the time interval appropriate?	Time interval appropriate		Doubtful whether time interval was appropriate or time interval was not stated	Time interval NOT appropriate	
3	Were the test conditions similar for the measurements? (e.g. type of administration, environment, instructions)	Test conditions were similar (evidence provided)	Assumable that test conditions were similar	Unclear if test conditions were similar	Test conditions were NOT similar	
Statistical methods						
4	For continuous scores: Was the Standard Error of Measurement (SEM), Smallest Detectable Change (SDC) or Limits of Agreement (LoA) calculated?	SEM, SDC, or LoA calculated	Possible to calculate LoA from the data presented		SEM calculated based on Cronbach's alpha, or on SD from another population	Not applicable
5	For dichotomous/nominal/ordinal scores: Was the percentage (positive and negative) agreement calculated?	% positive and negative agreement calculated	% agreement calculated		% agreement not calculated	Not applicable
Other						
6	Were there any other important flaws in the design or statistical methods of the study?	No other important methodological flaws		Other minor methodological flaws	Other important methodological flaws	

Box 8. Criterion validity

		very good	adequate	doubtful	inadequate	NA
Statistical methods						
1	For continuous scores: Were correlations, or the area under the receiver operating curve calculated?	Correlations or AUC calculated			Correlations or AUC NOT calculated	Not applicable
2	For dichotomous scores: Were sensitivity and specificity determined?	Sensitivity and specificity calculated			Sensitivity and specificity NOT calculated	Not applicable
Other						
3	Were there any other important flaws in the design or statistical methods of the study?	No other important methodological flaws		Other minor methodological flaws	Other important methodological flaws	

Box 9. Hypotheses testing for construct validity

9a. Comparison with other outcome measurement instruments (convergent validity)

Design requirements	**very good**	**adequate**	**doubtful**	**inadequate**	**NA**
1 Is it clear what the comparator instrument(s) measure(s)?	Constructs measured by the comparator instrument(s) is clear			Constructs measured by the comparator instrument(s) is not clear	
2 Were the measurement properties of the comparator instrument(s) sufficient?	Sufficient measurement properties of the comparator instrument(s) in a population similar to the study population	Sufficient measurement properties of the comparator instrument(s) but not sure if these apply to the study population	Some information on measurement properties of the comparator instrument(s) in any study population	No information on the measurement properties of the comparator instrument(s), OR evidence for insufficient measurement properties of the comparator instrument(s)	
Statistical methods					
3 Was the statistical method appropriate for the hypotheses to be tested?	Statistical method was appropriate	Assumable that statistical method was appropriate	Statistical method applied NOT optimal	Statistical method applied NOT appropriate	

Other					
4 Were there any other important flaws in the design or statistical methods of the study?	No other important methodological flaws		Other minor methodological flaws (e.g. only data presented on a comparison with an instrument that measures another construct)	Other important methodological flaws	

**9b. Comparison between subgroups (discriminative or known-groups validity)**					
Design requirements	**very good**	**adequate**	**doubtful**	**inadequate**	**NA**
5 Was an adequate description provided of important characteristics of the subgroups?	Adequate description of the important characteristics of the subgroups	Adequate description of most of the important characteristics of the subgroups	Poor of no description of the important characteristics of the subgroups		
Statistical methods					
6 Was the statistical method appropriate for the hypotheses to be tested?	Statistical method was appropriate	Assumable that statistical method was appropriate	Statistical method applied NOT optimal	Statistical method applied NOT appropriate	
Other					
7 Were there any other important flaws in the design or statistical methods of the study?	No other important methodological flaws		Other minor methodological flaws (e.g. only data presented on a comparison with an instrument that measures another construct)	Other important methodological flaws	

Box 10. Responsiveness

10a. Criterion approach (i.e. comparison to a gold standard)

	very good	adequate	doubtful	inadequate	NA
Statistical methods					
1 For continuous scores: Were correlations between change scores, or the area under the Receiver Operator Curve (ROC) curve calculated?	Correlations or Area under the ROC Curve (AUC) calculated			Correlations or AUC NOT calculated	Not applic able
2 For dichotomous scales: Were sensitivity and specificity (changed versus not changed) determined?	Sensitivity and specificity calculated			Sensitivity and specificity NOT calculated	Not applic able
Other					
3 Were there any other important flaws in the design or statistical methods of the study?	No other important methodological flaws		Other minor methodological flaws	Other important methodological flaws	

**10b. Construct approach (i.e. hypotheses testing; comparison with other outcome measurement instruments)**						
Design requirements		**very good**	**adequate**	**doubtful**	**inadequate**	**NA**
4	Is it clear what the comparator instrument(s) measure(s)?	Constructs measured by the comparator instrument(s) is clear			Constructs measured by the comparator instrument(s) is not clear	
5	Were the measurement properties of the comparator instrument(s) sufficient?	Sufficient measurement properties of the comparator instrument(s) in a population similar to the study population	Sufficient measurement properties of the comparator instrument(s) but not sure if these apply to the study population	Some information on measurement properties of the comparator instrument(s) in any study population	NO information on the measurement properties of the comparator instrument(s) OR evidence of poor quality of comparator instrument(s)	
Statistical methods						
6	Was the statistical method appropriate for the hypotheses to be tested?	Statistical method was appropriate	Assumable that statistical method were appropriate	Statistical method applied NOT optimal	Statistical method applied NOT appropriate	
Other						
7	Were there any other important flaws in the design or statistical methods of the study?	No other important methodological flaws		Other minor methodological flaws	Other important methodological flaws	

10c. Construct approach: (i.e. hypotheses testing: comparison between subgroups)

Design requirements	very good	adequate	doubtful	inadequate	NA
8 Was an adequate description provided of important characteristics of the subgroups?	Adequate description of the important characteristics of the subgroups	Adequate description of most of the important characteristics of the subgroups	Poor or no description of the important characteristics of the subgroups		
Statistical methods					
9 Was the statistical method appropriate for the hypotheses to be tested?	Statistical method was appropriate	Assumable that statistical method was appropriate	Statistical method applied NOT optimal	Statistical method applied NOT appropriate	
Other					
10 Were there any other important flaws in the design or statistical methods of the study?	No other important methodological flaws		Other minor methodological flaws	Other important methodological flaws	

**10d. Construct approach: (i.e. hypotheses testing: before and after intervention)**					
Design requirements	**very good**	**adequate**	**doubtful**	**inadequate**	**NA**
11 Was an adequate description provided of the intervention given?	Adequate description of the intervention		Poor description of the intervention	NO description of the intervention	
Statistical methods					
12 Was the statistical method appropriate for the hypotheses to be tested?	Statistical method was appropriate	Assumable that statistical method was appropriate	Statistical method applied NOT optimal	Statistical method applied NOT appropriate	
Other					
13 Were there any other important flaws in the design or statistical methods of the study?	No other important methodological flaws		Other minor methodological flaws	Other important methodological flaws	
