# Supplementary material for: Imagery ability assessments: a cross-disciplinary systematic review and quality evaluation of psychometric properties
Source: BMC Med. 2022 May 2;20:166. doi: 10.1186/s12916-022-02295-3 (PMC9059408; doi:10.1186/s12916-022-02295-3)
Supplement: Supplementary file 3 — Additional file 3: Table 1S. Characteristics of the Included Measurement Tools for Motor Imagery. [file 12916_2022_2295_MOESM3_ESM.pdf]

Table 1A. Characteristics of the included measurement tools for motor imagery

Tool	Original language	Available translations	Construct(s)	Test format	Mode of administration	Scale/ Subscales (SS)	No. of items	Scoring	Equipment	Time needed (Min.)	Comments
Florida Praxis Imagery Questionnaire (FPIQ)	English	German	Explicit movement imagery	questionnaire	self-report	4 SS, kinaesthetic, position, action and object	48 (12 for each subscale)	each question has two possible responses	questionnaire	NR	-
Imaprax	English French	German	Motor imagery vividness	Computer and video-based assessment + questionnaire	examiner-administered	visual	6 complex unilateral and bilateral UL gestures or ADL's	7-point scale	Imaprax software, Laptop, chair, questionnaire	20-25	-
Kinaesthetic and Visual Imagery Questionnaire (KVIQ-20)	English	German Brazilian Portuguese Japanese	Vividness kinaesthetic and visual motor imagery	questionnaire	examiner-administered	2 SS, kinaesthetic and visual	20	5-point scale	Questionnaire, one chair with backrest for participant and one chair for the examiner	20-30	Russian, Spanish, Chinese versions of the KVIQ also exist, but no validation studies were available.
Kinaesthetic and Visual Imagery Questionnaire (KVIQ-10)	English	German Japanese French	Vividness kinaesthetic and visual motor imagery	questionnaire	examiner-administered	2 SS, kinaesthetic and visual	10	5-point scale	Questionnaire, one chair with backrest for participant and one chair for the examiner	15-20	
Movement Imagery Questionnaire (MIQ)	English	-	Vividness kinaesthetic and visual motor imagery	questionnaire	self-report	2 SS, kinaesthetic and visual	18	7-point scale	Questionnaire, calm room, one chair	NR	-
Revised Movement Imagery Questionnaire (MIQ-R)	English	-	Vividness kinaesthetic and visual motor imagery	questionnaire	self-report	2 SS, kinaesthetic and visual	8	7-point scale	Questionnaire, calm room, one chair	10-15	-
Movement Imagery Questionnaire-Revised second version (MIQ-RS)	English	French	Vividness kinaesthetic and visual motor imagery	questionnaire	self-report	2 SS, kinaesthetic and visual	14	7-point scale	Questionnaire, calm room, one chair	20-25	-
Movement Imagery Questionnaire-3 (MIQ-3)	English	Slovene Polish French Spanish Turkish	Vividness of external + internal visual and kinaesthetic motor imagery	questionnaire	self-report	3 SS, external visual, internal visual and kinaesthetic imagery	12	7-point scale	Questionnaire, calm room	20-30	-
Movement Imagery Questionnaire for Children (MIQ-C)	English	-	Vividness of external + internal visual and kinaesthetic motor imagery	questionnaire	self-report	3 SS, external visual, internal visual and kinaesthetic imagery	12	7-point scale	Questionnaire, calm room	30	-

Test of Ability in Movement Imagery (TAMI)	English	-	Ability to imagine a series of movement imagery	questionnaire	self-report	No SS	10 questions	Only correct answers will be scored 0-10	Questionnaire, calm room	10	TAMI-weighted (TAMIw): new scoring method was introduced. More difficult questions were weighted more heavily than relatively easier questions. TAMIw Max. Score is 24.
Test of Ability in Movement Imagery with Hands (TAMI-H)	English	-	Ability to imagine hand movements	questionnaire	self-report	2 SS, Functionally-involved movement and Isolated movement	44 questions	Only correct answers in a multiple-choice format will be scored	Questionnaire, calm room, one chair	NR	The questionnaire was divided into 4 booklets: 2 tested the imagined movements of the right hand and the other 2 tested imagined movements of the left hand.
Vividness of Movement Imagery Questionnaire (VMIQ)	English	-	Vividness kinaesthetic and visual movement imagery	questionnaire	self-report	2 SS, kinaesthetic and visual	24	5-point scale	Questionnaire, calm room	NR	-
Vividness of Haptic Movement Imagery Questionnaire (VHMIQ)	English	-	Vividness of hapic visual imagery	questionnaire	self-report	Only visual imagery	24	5-point scale	Questionnaire, calm room	NR	Campos et al. 1998 provided an English version of VHMIQ as Appendix. But it is not clear whether a Spanish version is also available.
Revised Vividness of Movement Imagery Questionnaire-2 (VMIQ-2)	English	German	Vividness kinaesthetic and visual movement imagery	questionnaire	self-report	3 SS, external, internal, kinaesthetic	12	5-point scale	Questionnaire, calm room	NR	-
Wheelchair Imagery Ability Questionnaire (WIAQ)	English	-	Imagery ability by athletes with a disability	questionnaire	self-report	3 SS, external, internal, kinaesthetic	24	5-point scale	Questionnaire, calm room	NR	-

Abbreviations: Min.=minutes; NR=not reported; UL=upper limb; ADL=activities of daily living; external=external perspective subscale; internal=internal perspective subscale; Functionally-involved movement=imagery of hand movements interacting with objects; Isolated movement=imagery of hand movements in the absence of objects;
