# Supplementary material for: Imagery ability assessments: a cross-disciplinary systematic review and quality evaluation of psychometric properties
Source: BMC Med. 2022 May 2;20:166. doi: 10.1186/s12916-022-02295-3 (PMC9059408; doi:10.1186/s12916-022-02295-3)
Supplement: Supplementary file 4 — Additional file 4: Table 2S. Motor imagery: Summary of Findings using modified GRADE. [file 12916_2022_2295_MOESM4_ESM.pdf]

Table 2A. Motor imagery assessments: Summary of Findings using modified GRADE

Structural validity	Summary of results	Overall rating of each measurement property	Quality of evidence	Comments
**MIQ-R (Monsma et al. 2009, Williams et al. 2012)**	Bifactorial structure (kinaesthetic and visual) confirmed. Total sample size 725 athletes.	sufficient	High: both studies with very good methodological quality and consistent results.	Results exist only for athletes with a mean age of 20.0 years.<br>Further studies are needed including older populations.
**MIQ-3 (Williams et al. 2012, Budnik-Przybylska et al. 2016)**	The three-factor structure (external, internal and kinaesthetic) confirmed. Total sample size 646 athletes.	sufficient	High: both studies with very good methodological quality and consistent results.	Results were pooled only for English version.<br>The results for Turkish version seem to be identical.<br>Slovenian, French and Spanish versions indicate insufficient structural validity.<br>Further studies are needed including other populations.
**VMIQ-2 (Roberts et al. 2008 1 +2)**	The three-factor structure (external, internal and kinaesthetic) confirmed. Total sample size 706 athletes.	sufficient	High: both studies with very good methodological quality and consistent results.	Results of three separate studies were reported in this paper, which allows the summary of results from two studies.<br>The German version was evaluated with students and indicated a sufficent structural validity.
**Internal consistency**	**Summary of results**	**Overall rating of each measurement property**	**Quality of evidence**	**Comments**
**MIQ (Hall et al. 1985,<br>Atienza & Balaguer 1994)**	Cronbach alpha 0.88-0.91 kinaesthetic and 0.87-0.89 visual. Total sample size 190 students.	indeterminate	High: both studies with very good methodological quality for this research question and consistent results.	Lack of evidence for sufficient structural validity. Results exist only for students.<br>Further studies are needed including other populations and population with large age range.
**MIQ-R (Monsma et al. 2009, Williams et al. 2012)**	Cronbach=0.88 respectively CR 0.82 kinaesthetic, Cronbach=0.84 respectively CR=0.88 visual. Total sample size 725 athletes.	sufficient	High: both studies with very good methodological quality for this research question and consistent results.	Results exist only for athletes with a mean age of 20.0 years. Further studies are needed including older population.
**MIQ-3 (Williams et al. 2012, Budnik-Przybylska et al. 2016)**	Cronbach=0.81 respectively CR=0.85 and CR= 0.89 kinaesthetic, Cronbach=0.78 respectively CR=0.79 and CR=0.81 internal, Cronbach=0.75 respectively CR=0.83 and CR=0.89. Total sample size 743 athletes.	sufficient	High: all studies with very good methodological quality for this research question and consistent results.	Results exist only for athletes with a mean age of 20.0 years and only for English version of MIQ-3. Further studies are needed including older populations.
**VMIQ-2 (Williams et al. 2012 1+2 and Roberts et al. 2008[3])**	Cronbach=0.93 respectively CR= 0.93 for kinaesthetic subscale, Cronbach=0.95 respectively CR=0.93 and CR=0.92 for internal subscale, Cronbach=0.95 respectively CR=0.94 and CR=0.93 for external subscale. Total sample size 538 athletes.	sufficient	High: all studies are methodological very good.	Williams et al. and Roberts et al. reported results of several studies. Results exist only for athletes.<br>Further studies are needed including other populations.
**Reliability- test-retest**	**Summary of results**	**Overall rating of each measurement property**	**Quality of evidence**	**Comments**

**KVIQ-20 (Malouin 2007, Schuster et al. 2012, Randhawa et al. 2010, Tabrizzi et al. 2013)**	ICC range 0.75-0.91 for kinaesthetic and 0.77-0.84 for visual. Total 77 stroke patients.<br><br>ICC 0.82- 0.95 for kinaesthetic and 0.68-0.82 for visual. Total 19 PD patients.<br><br>ICC 0.93- 0.95 for kinaesthetic and 0.43-0.85 for visual. Total 22 MS patients.	sufficient<br><br>indeterminate<br><br>indeterminate	Low: three studies are of doubtful quality and one study of inadequate quality. Two studies with small sample size (≤15).	Different subgroups of patients with neurological but also othopedical diseases were evaluated.<br>ICC for visual scale was low (<0.70) for MS and PD group in one study and indicated test-retest insufficient of KVIQ for this population.<br>However, this study (Schuster et al. 2012) had very low sample size (N=7 MS and N=8 PD patients).
**Construct validity- Hypotheses testing**	**Summary of results**	**Overall rating of each measurement property**	**Quality of evidence**	**Comments**
**VMIQ (Isaac et al. 1986, Eton et al.1998, Lequerica et al. 2002)**	No hypothesis defined. Insufficient information about comparator instrument.	indeterminate	Very low: two studies with inadequate and one with doubtful methodological quality.	Insufficient information about comparator instrument.
**TAMI (Madan & Singhal 2013, Madan & Singhal 2014)**	No hypothesis defined. Insufficient information about comparator instrument.	indeterminate	Very low: both studies inadequate methodological quality.	Insufficient information about comparator instrument.
**Abbrevations**: MIQ=Movement Imagery Questionnaire; MIQ-R=Revised Movement Imagery Questionnaire, MIQ-3=Movement Imagery Questionnaire-3; VMIQ=Vividness of Movement Imagery Questionnaire; VMIQ-2=Revised Vividness of Movement Imagery Questionnaire-2; TAMI=Test of Ability in Movement Imagery; CR=composite reliability; PD=Parkinson disease; MS=Multiple sclerosis;				
