# Supplementary material for: Imagery ability assessments: a cross-disciplinary systematic review and quality evaluation of psychometric properties
Source: BMC Med. 2022 May 2;20:166. doi: 10.1186/s12916-022-02295-3 (PMC9059408; doi:10.1186/s12916-022-02295-3)
Supplement: Supplementary file 5 — Additional file 5: Table 3S. Characteristics of the Included Measurement Tools for Mental Imagery. [file 12916_2022_2295_MOESM5_ESM.pdf]

Table 3A. Characteristics of the included measurement tools for mental imagery

a. General mental imagery assessment (measuring imagery in any sensorial modality)											
**Tool**	**Original language**	**Available translations**	**Construct(s)**	**Test format**	**Mode of administration**	**Scale/ Subscale (SS)**	**No. of items/ Questions**	**Scoring**	**Equipment**	**Time needed (Min.)**	**Comments**
Auditory Imagery Scale (AIS)	English	Spanish	Vividness of auditory imagery	questionnaire	self-administered	1 SS	7	4-point scale	questionnaire	NR	-
Auditory Imagery Questionnaire (AIQ)	English	Spanish	Vividness of auditory imagery	questionnaire	self-administered	1 SS	12	5-point scale	questionnaire	20-30 Min.	-
Bucknell Auditory Imagery Scale (BAIS)	English	none	Vividness and control of auditory imagery	questionnaire	self-administered	2 SS, control and vividness	14	7-point scale	questionnaire	NR	-
Betts Questionnaire Upon Mental Imagery (original QUMI)	English	none	Vividness of mental imagery in any sensorial modality	questionnaire	self-administered	7 SS, visual, auditory, cutaneous, kinaesthetic, gustatory, olfactory, organic	150	7-point scale	questionnaire	NR	Betts developed QUMI but did not evaluate psychometric properties.
Betts Questionnaire Upon Mental Imagery (shorted, SQMI)	English	Spanish Italian	Vividness of mental imagery in any sensorial modality	questionnaire	self-administered	7 SS, visual, auditory, cutaneous, kinaesthetic, gustatory, olfactory, organic	35	7-point scale	questionnaire	55 Min.	Evaluated with students only.
Clarity of Auditory Imagery Scale (CAIS)	English, Swedish	Spanish Polish	Clarity of auditory imagery	questionnaire	self-administered	1 SS	16	5-point scale	questionnaire	NR	-
Gordon Test of Visual imagery control (GTVIC)	English	Spanish French	Controllability of visual imagery	questionnaire	self-administered	No SS	12	3-point scale	questionnaire	NR	-
Imaging Ability Questionnaire (IAQ)	English	none	Ability to image and to experience those images	questionnaire	self-administered	2 SS, absorption and image generation	54	5-point scale	questionnaire	NR	Short version consists of 34 items, scoring 0-4. Time needed 10 Min.
Imagery Questionnaire by Lane	English	none	Control of imagery	questionnaire	self-administered	7 SS, visual, auditory, cutaneous, kinaesthetic, gustatory, olfactory, organic	35	5-point scale	questionnaire	NR	-
Kids Imaging Ability Questionnaire (KIAQ)	English	none	Ability to image and to experience those images	questionnaire	self-administered	2 SS, absorption and image generation	17	3-point scale	questionnaire	10 Min.	-
Mental imagery Scale (MIS)	Italian	none	Process of generation and qualities of a mental imagery	questionnaire	self-administered	6 SS, Image Formation Speed, Permanence/Stability, Dimensions, Level of Detail and Grain, Distance, Depth of Field or Perspective	33	5-point scale	questionnaire and separate page with text	NR	-

Plymoth sensory imagery Questionnaire (Psi-Q)	English	none	Vividness of mental imagery across sensory modalities	questionnaire	self-administered	7 SS, visual, auditory, olfactory, taste, touch, bodily sensation, emotional feelings	35	11-point scale	questionnaire	NR	-
Sport Imagery Ability Measure (SIAM)	English	none	Five imagery dimensions: vividness, control, ease, speed, duration	questionnaire	self-administered	6 SS, visual, auditory, kinaesthetic, olfactory, gustatory, tactile, emotion	72	10-cm analogue scales	questionnaire	NR	-
Revised Sport Imagery Ability Measure (SIAM)	English	none	Five imagery dimensions: vividness, control, ease, speed, duration	questionnaire	self-administered	6 SS, visual, auditory, kinaesthetic, olfactory, gustatory, tactile, emotion	48	10-cm analogue scales	questionnaire	NR	-
Sport Imagery Ability Questionnaire (SAIQ)	English	none	Athletes ease and vividness of imaging on different content: cognitive specific, motivational specific, general imagery,	questionnaire	self-administered	4 SS, skill, strategy, goal and affect	20	7-point scale	questionnaire	NR	First version 35 items. Short version with 12 items and a modified version with 15 items available. Scoring and subscales are the same for each version.
Survey of mental imagery	English	French	Vividness and controllability of mental imagery	questionnaire, two forms: A and B	self-administered	7 SS, visual, auditory, olfactory, gustatory, tactile, somesthetic, kinaesthetic	86	vividness: 1-5 control: 1-3	questionnaire	20 Min.	-
Visual Elaboration Scale (VES)	English	none	Visual imagery ability- differences in visualisation	questionnaire	examiner-administered	4 scenes	15	0-15 points	questionnaire	NR	-
Vividness of Olfactory Imagery Questionnaire (VOIQ)	English	none	Vividness of olfactory imagery	questionnaire	self-administered	1 SS	16	5-point scale	questionnaire	NR	-
Vividness of Object and Spatial Imagery Questionnaire (VOSI)	NR	English	Vividness object and spatial imagery	questionnaire	self-administered	2 SS, spatial and object imagery	28	5-point scale	questionnaire	NR	Questionnare only avalible in English. No information in what language the VOSI was developed.
Vividness of Visual Imagery Questionnaire (VVIQ)	English	Spanish	Vividness visual imagery	questionnaire	self-administered	No SS, eyes open and closed version	16	5-point scale	questionnaire	NR	-
Revised version Vividness of Visual Imagery Questionnaire (VVIQ-2)	English	Spanish	Vividness visual imagery	questionnaire	self-administered	No SS	32	5-point scale	questionnaire	NR	-

Vividness of Visual Imagery Questionnaire-Revised version (VVIQ-RV)	English	Spanish	Vividness visual imagery	questionnaire	self-administered	No SS	32	7-point scale	questionnaire	NR	-
Vividness of Visual Imagery Questionnaire-Modified (VVIQ-M)	English	none	Vividness visual imagery	questionnaire	self-administered	No SS	16	7-point scale	questionnaire	NR	-
Vividness of Wine Imagery Questionnaire (VWIQ)	English	none	Vividness of mental imagery of wine	questionnaire	self-administered	Six different scenarios: vineyard, restaurant, bistro, a relaxing night at home, and two wine tastings	18	5-point scale	questionnaire	NR	The questionnaire could be a useful measure of individual wine experience for use in the wine industry.
**b. Assessments of spatial imagery- mental rotation**											
Card Rotation Test	English	none	Ability and speed to rotate mental images	questionnaire	self-administered	No SS	10	2 choice correct and incorrect	questionnaire	3 Min.	-
Cube-Cutting Task (CCT)	English	none	Ability of mental manipulation and requires image generation	questionnaire	self-administered	No SS	3	0-3	questionnaire	NR	-
German Test of the Controllability of Motor Imagery (TKBV)	German	none	Ability to manipulate mental imagined one body part	questionnaire	self-administered	2 SS, Recognition and Free recall	20	0-70 points (high scores= good ability)	questionnaire with pictures	60 Min.	-
Hand laterality task	German	none	Ability to rotate mental images and left-right decision	questionnaire	self-administered	No SS	128 trials	0-128	questionnaire with pictures	15 Min.	Four different pictures: a hand, a foot, a face of woman, a car.
Judgement test of foot and trunk laterality	Swedish	none	Ability to rotate mental images and left-right decision	Web-based questionnaire	self-administered	No SS	60 pictures	Max. 60 (range 0-60)	Computer and specific software Recognise Online^{TM}	NR	60 pictures of trunk and foot to determine left or right.
Map Rotation Ability Test (MRAT)	Spanish	English	Ability to rotate a map	questionnaire with pictures	self-administered	Maps rotated either at 0º, 90º, or 180º	30	correct responses minus incorrect responses: 30 to -30	questionnaire with pictures (maps)	2 Min.	-
Mental Paper Folding	English	none	Measure of mental manipulation	paper-and-pencil test	self-administered	No SS	10	Each items was rated as correct or incorrect	questionnaire	45-60 Min.	-

Mental Rotation of Three-Dimensional Objects	English	none	Measure of tree-dimensional spatial visualization ability	Paper-and-pencil test of spatial visualization	self-administered	No SS	20	2 choice correct and two incorrect per item	questionnaire	10Min.	There is another version with 24 items.
Measure of the Ability to Form Spatial Mental imagery (MASMI)	Spanish	none	Ability to form spatial imagery	paper-and-pencil test	self-administered	No SS	23	Each question multiple choices, lowest -46, highest 46	questionnaire	10 Min.	-
Measure of the Ability to Rotate Mental Images (MARMI)	Spanish	none	Ability to rotate mental images (spatial imagery ability)	questionnaire	self-administered	No SS	23	Max. 46 (range: -46-46)	questionnaire	10 Min.	Each question has four options to answer: 2 correct and 2 incorrect.
Shoulder specific left right judgement task (LRJT)	English	none	Ability to rotate mental images and left-right decision	Web-based questionnaire	self-administered	No SS	40 pictures	0-40	Computer and specific software Recognise Online^{TM}	NR	-
Spatial Orientation Skills Test (SOST)	Spanish	English	Ability to orientate oneself on map	Map with a black point, which indicates the location	self-administered	Ten pairs are positioned at 0º, 10 at 90º, and 10 at 180º.	30	correct responses minus incorrect responses: 30 to -30	Map with the mark (black point)	2 Min.	-
**c. Assessments of mental imagery to distinguish between different types of imagers**											
Object-Spatial Imagery Questionnaire (OSIQ)	English	none	Designed to distinguish between: 1) object imagers who prefer to use object imagery 2) spatial imagers who prefer to use a spatial imagery	questionnaire	self-administered	2 SS, spatial and object imagery	30	5-point scale	questionnaire	NR	-
Object-Spatial Imagery and Verbal Questionnaire (OSVIQ)	English	none	Designed to distinguish between: 1) object imagers who prefer to use object imagery 2) spatial imagers who prefer to use a spatial imagery, 3) verbalizers who prefer to use verbal-analytical tools	questionnaire	self-administered	3 SS, spatial, object and verbal	45	5-point scale	questionnaire	NR	-
Pavio's Individual Differences Questionnaire (IDQ, 86-items)	English	none	Verbal and imaginal habits, preferences and abilities	questionnaire	self-administered	2 SS, verbal and imaginal	86	true-falsh	questionnaire	NR	Original version.

Revised Pavio's Individual Differences Questionnaire (IDQ, 34-items)	English	none	Verbal and imaginal habits, preferences and abilities	questionnaire	self-administered	2 SS, verbal and imaginal	34	true-falsh	questionnaire	NR	This version was revised by Kardash 1986.
Pavio's Individual Differences Questionnaire (shorted IDQ, 72-items)	English	none	Verbal and imaginal habits, preferences and abilities	questionnaire	self-administered	2 SS, verbal and imaginal	72	true- falsh	questionnaire	NR	This version was revised by Hiscock 1978.
Sussex Cognitive Styles Questionnaire (SCSQ)	English	none	Visual and verbal processing preferences	questionnaire	self-administered	6 SS, Imagery Ability Technical / Spatial Language & Word Forms Need for Organisation Global Bias Systemising Tendency	84	5-point scale	questionnaire	NR	-
Verbalizer-Visualizer Questionnaire (VVQ)	English	German, Spanish	Distinguish between visualizer and verbalizer	questionnaire	self-administered	2 SS, verbal and visual	15	yes- no	questionnaire	NR	-
**d. Assessments of use of mental imagery**											
Children's Active Play Imagery Questionnaire (CAPIQ)	English	Persian	Frequency of imagination by children	questionnaire	self-administered	No SS	11	5-point scale	questionnaire	NR	-
Exercise Imagery Questionnaire-Aerobic Version EIQ-AV	English	none	Use of exercise imagery	questionnaire	self-administered	3 SS, Appearance, Energy, and Technique	9	9-point scale	questionnaire	10 Min.	First version consisted of 23-items.
Sport Imagery Questionnaire (SIQ)	English	none	Use of mental imagery in relation to cognitive and motivational issues	questionnaire	self-administered	5 SS, cognitive general, cognitive specific, motivational specific, motivational general arousal, motivational general mastery	30	7-point scale	questionnaire	NR	First version consisted of 46-items and 4 scales.
Sport Imagery Questionnaire for Children (SIQ-C)	English	none	Use of mental imagery in relation to cognitive and motivational issues	questionnaire	self-administered	5 SS, cognitive general, cognitive specific, motivational specific, motivational general arousal, motivational general mastery	21	5-point scale	questionnaire	NR	-
Spontaneous Use of Imagery Scale (SUIS)	English	Dutch German	Frequency and likelihood of use of mental imagery in daily life	questionnaire	self-administered	No SS	12	5-point scale	questionnaire	NR	-
**Abbreviations**: Min.=minutes; Max.= Maximum; NR= not reported;											
