# Supplementary material for: Imagery ability assessments: a cross-disciplinary systematic review and quality evaluation of psychometric properties
Source: BMC Med. 2022 May 2;20:166. doi: 10.1186/s12916-022-02295-3 (PMC9059408; doi:10.1186/s12916-022-02295-3)
Supplement: Supplementary file 6 — Additional file 6: Table 4S. Mental imagery Assessments: Summary of Findings using modified GRADE. [file 12916_2022_2295_MOESM6_ESM.pdf]

Table 4A. Mental imagery assessments: Summary of Findings using modified GRADE

Structural validity	Summary of results	Overall rating of each measurement property	Quality of evidence	Comments
**Psi-Q (Andrade et al. 2014[1] + 2014[2])**	Study[1]: 7 factors were identified. Factors loading 0.53-0.87.<br>Study[2]: showed a good model fit with 7 factors. Total sample size was 613 students.	indeterminate	Moderate: only two studies available with very good doubtful methodological quality, respectively.	Results from several studies in this paper reported.<br>The quality of the assessment was rated as indeterminate as no details were reported.
**SQMI (White et al. 1974, Campos and Pérez-Fabello 2005, Barnchok 1995)**	Two studies identified 7 factors (English version of SQMI). Factor loadings 0.20-0.89. One item on visual subscale loaded <0.20. Total sample size 2219 students.	indeterminate	High: all studies with adequate methodological quality for this research question.	Although all studies were rated as adequate, structural validity was rated as indeterminate because no study carried out a confirmatory factor analysis.
**SIAQ (Williams & Cumming 2011[3] + 2011[4])**	Modified version (15 items and 5 subscales) was evaluated. CFA confirmed five-factor model. Factor loadings 0.62-0.88. Total sample size 646 athletes.	sufficient	High: two studies by same author available. Both studies were rated as methodologically very good.	Results of four separate studies were reported in this paper, which allows the summary of results from two studies.<br>However, results exist only for athletes.
**SIQ (Hall et al. 1998, Ruiz et al. 2014)**	30-items version was evaluated. Five-factor structure confirmed: cognitive general, cognitive specific, motivational specific, motivational general arousal, motivational general mastery. Total sample size 632 athletes.	sufficient	Moderate: two studies available with adequate and very good methodological quality.	The quality of assessment was rated as sufficient but only one study conducted CFA and reported model fits.
**SIQ-C (Hall et al. 2009[1] + 2009[2])**	CFA confirmed five-factor model. Total sample size 1056 young athletes.	insufficient	Low: two studies available and both with doubtful methodological quality.	The quality of assessments was rated in one study as insufficient because model fits were at the limit or not acceptable.
**VVIQ (Rossi 1977, Lorenz & Neisser 1985, Kihlstrom 1991, LeBoutillier 2001)**	All studies performed only a PCA. Factors extracted were very different form single factor to four factor. Total sample size 3168 students.	indeterminate	Low: one study with inadequate, two studies with doubtful and one study with adequate methodological quality. Results are inconsistent.	No study performed a confirmatory factor analysis.<br>Results obtained by PCA varied widely.

Internal consistency	Summary of results	Overall rating of each measurement property	Quality of evidence	Comments
**GTVIC (Juhasz 1972, Westcott et al. 1976, Pérez-Fabello 2004)**	Cronbach alpha 0.64-0.88. Total sample size 693 students.	insufficient	Low: one study with very good and two studies with doubtful methodological quality.	Cronbachs alpha only in one study >0.70.<br>Results were summarized for two language versions: Spanish and English.<br>No differences found regarding internal consistency.
**MASMI (Campos 2009, Campos 2013)**	Cronbach alpha 0.93. Total sample size 392 students.	indeterminate	High: Both studies available with very good methodological quality.	The quality of assessment was rated as indeterminate because details regarding structural validity reported in studies were missing.
**Psi-Q (Andrade et al. 2014[1] + 2014[2] + 2014[3])**	Cronbach's alpha 0.93-0.96.<br>Total sample size 825 students.	insufficient	Very low: all studies with inadequate methodological quality.	Cronbach alpha calculated for total score, not for each scale.<br>Results exist only for students.
**SQMI (Juhasz 1972, Westcott et al. 1976, Barnchok 1995, Sacco and reda 1998, Campos and Pérez-Fabello 2005)**	Cronbach alpha total score ranged 0.86-0.95. Auditory 0.65-0.70, kinaesthetic 0.58-0.67, gustatory 0.63-0.76, olfactory 0.64-	insufficient	Low: three studies with inadequate and two studies with very good methodological	Only two studies reported Cronbach's alpha for each subscale.

	0.72, organic 0.67-0.75, cutaneous 0.62-0.64, visual 0.66-0.67. Total sample size 1634 students.		quality for this research question but consistent results.	One study evaluated Spanish version of SQMI. Results exist only for students. Further studies are needed including other populations.
**VVIQ (Campos & Pérez-Fabello 2002, Campos & Pérez-Fabello 2009)**	Cronbach alpha 0.88, respectively 0.91. Total sample size 1129 students.	sufficient	High: two studies available with very good methodological quality.	Results summarised only for Spanish version. Only students evaluated.
**VVIQ-2 (Campos & Pérez-Fabello 2009, Campos 2011)**	Cronbach alpha 0.88, respectively 0.91. Total sample size 485 students.	indeterminate	High: two studies available with very good methodological quality.	Results summarised only for Spanish version. The quality of assessment was rated indeterminate because details regarding structural validity were missing.
**SIAQ (Williams & Cumming 2011[3] + 2011[4])**	15-items version evaluated. CR ranged 0.76-0.86. Total sample size 646 athletes.	sufficient	High: two studies available with very good methodological quality.	CR in both studies > 0.70. However, results exist only for athletes.
**Construct validity- Hypotheses testing**	**Summary of results**	**Overall rating of each measurement property**	**Quality of evidence**	**Comments**
**GTVIC (Kihlstrom 1991, Lequerica 2002)**	Corr. between GTVIC and VMIQ visual subscale was r=0.72), GTVIC and SQMI was r=0.25, GTVIC and VVIQ r=45 and GTVIC and MIQ r=0.45. Total sample size 2885 students.	indeterminate	Low: only two studies available, one adequate and another inadequate methodological quality.	Study with higher sample size rated as inadequate regarding of methodological quality. No hypotheses defined and no psychometric properties of comparator instrument reported.
**VVIQ-2 (Campos & Pérez-Fabello 2009, Campos 2011)**	High corr. (r= 0.51-0.55) was found between VVIQ-2 and assessments of object imagery. Low corr. between VVIQ-2 and verbal or spatial imagery (r =0.7-0.23). Total sample size 485 students.	indeterminate	Moderate: only two studies, one adequate and another doubtful methodological quality.	In one study with higher sample size no hypotheses was defined and the quality of the assessment was rated as indeterminate. Results exist only for students. Further studies are needed including other populations.
**Abbrevations**: SQMI=Betts Questionnaire Upon Mental Imagery (shorted 35-items); Psi-Q=Plymouth sensory imagery Questionnaire; VVIQ=Vividness of Visual Imagery Questionnaire; VVIQ-2=Revised version Vividness of Visual Imagery Questionnaire; MASMI=Measure of the Ability to Form Spatial Mental Imagery; GTVIC=Gordon Test of Visual Imagery Control; SIAM=Sport Imagery Ability Measure; SIAQ=Sport Imagery Ability Questionnaire; SIQ=Sport Imagery Questionnaire; SIQ-C=Sport Imagery Questionnaire for Children; VMIQ=Vividness of Movement Imagery Questionnaire; MIQ=Movement Imagery Questionnaire; PCA=Principal Component Analysis; CFA=Confirmatory Factor Analysis;				
